# Supplementary material for: Design, synthesis, and biological activity of novel halogenated sulfite compounds
Source: PLoS One. 2025 Jul 2;20(7):e0327587. doi: 10.1371/journal.pone.0327587 (PMC12220988; doi:10.1371/journal.pone.0327587)
Supplement: S1 File — (DOCX) [file pone.0327587.s001.docx]

**S1: The ^1^H NMR, ^13^C NMR Spectra, and HRMS of compounds 5.01-5.36**


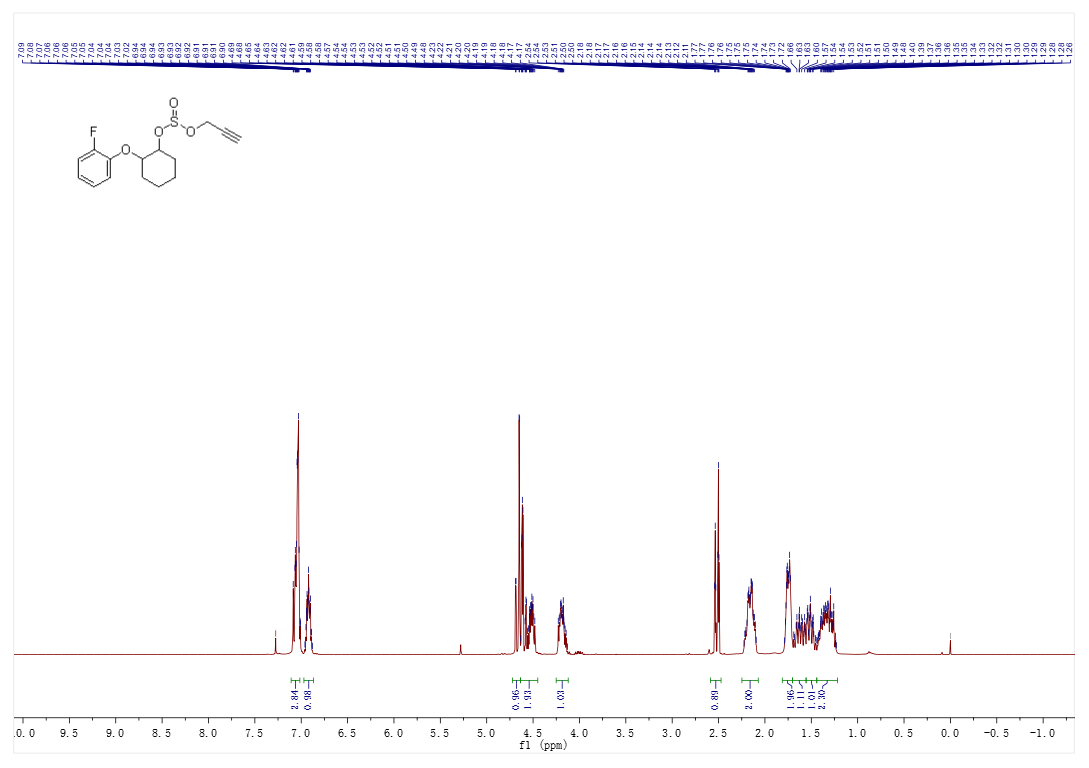


**Figure S1**. ^1^H NMR spectrum of compound *2-(2-fluorophenoxy)cyclohexyl prop-2-yn-1-yl sulfite* (**5.01**)


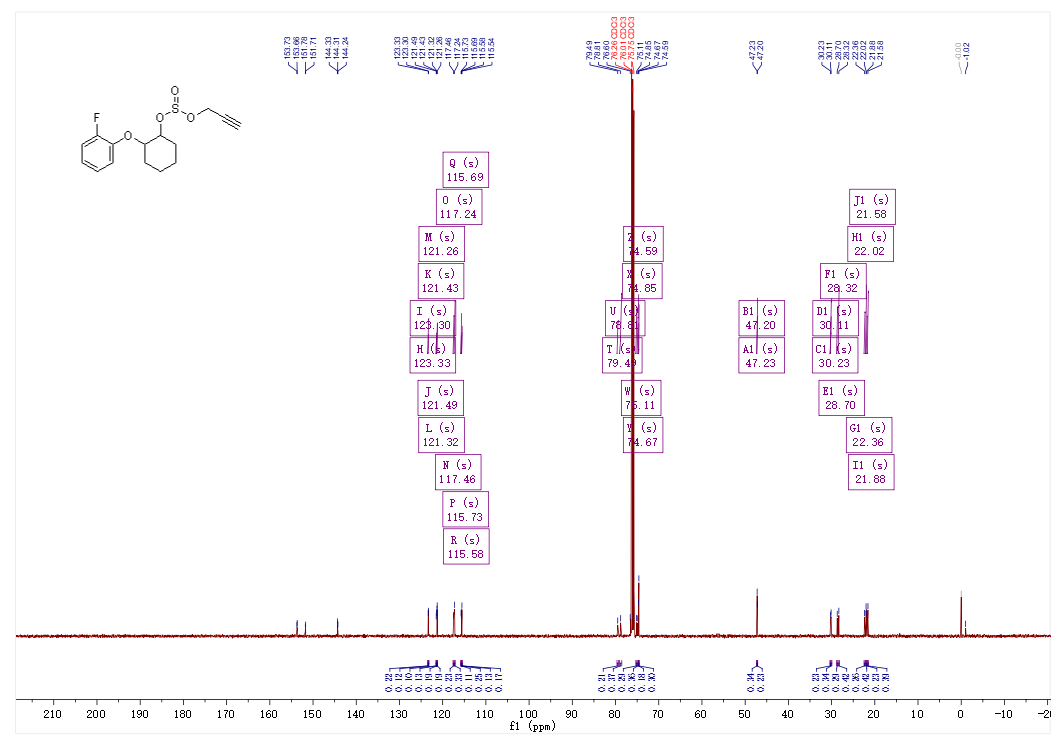


**Figure S2**. ^13^C NMR spectrum of compound *2-(2-fluorophenoxy)cyclohexyl prop-2-yn-1-yl sulfite* (**5.01**)


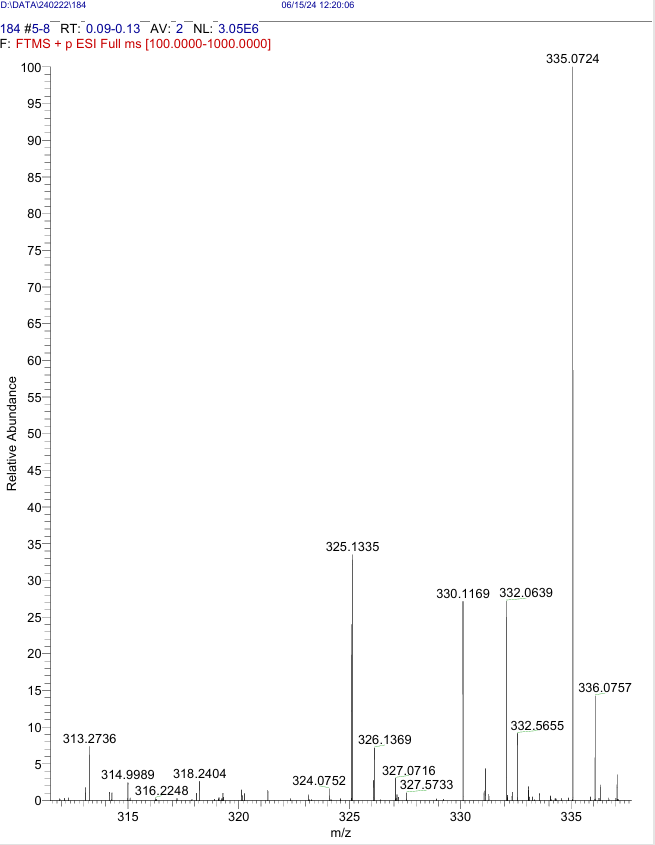


**Figure S3**. HRMS Spectrum of compound *2-(2-fluorophenoxy)cyclohexyl prop-2-yn-1-yl sulfite* (**5.01**)


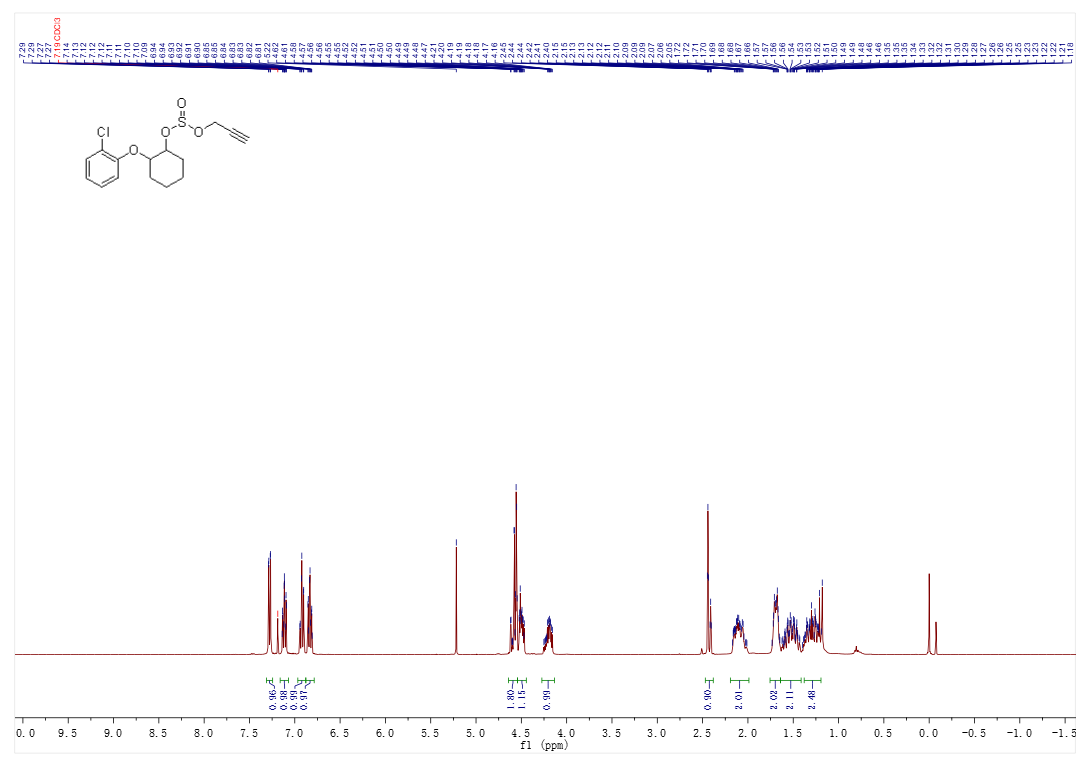


**Figure S4**. The ^1^H NMR spectrum of compound *2-(2-chlorophenoxy)cyclohexyl prop-2-yn-1-yl sulfite* (**5.02**)


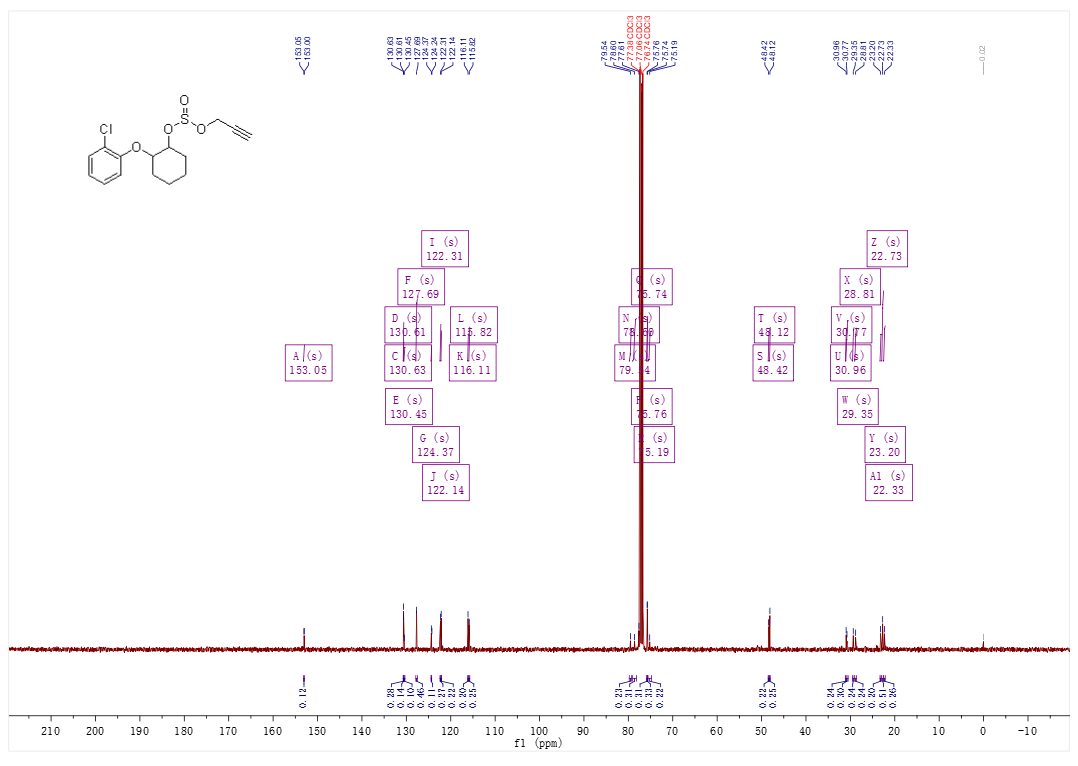


**Figure S5.** The ^13^C NMR spectrum of compound *2-(2-chlorophenoxy)cyclohexyl prop-2-yn-1-yl sulfite* (**5.02**)


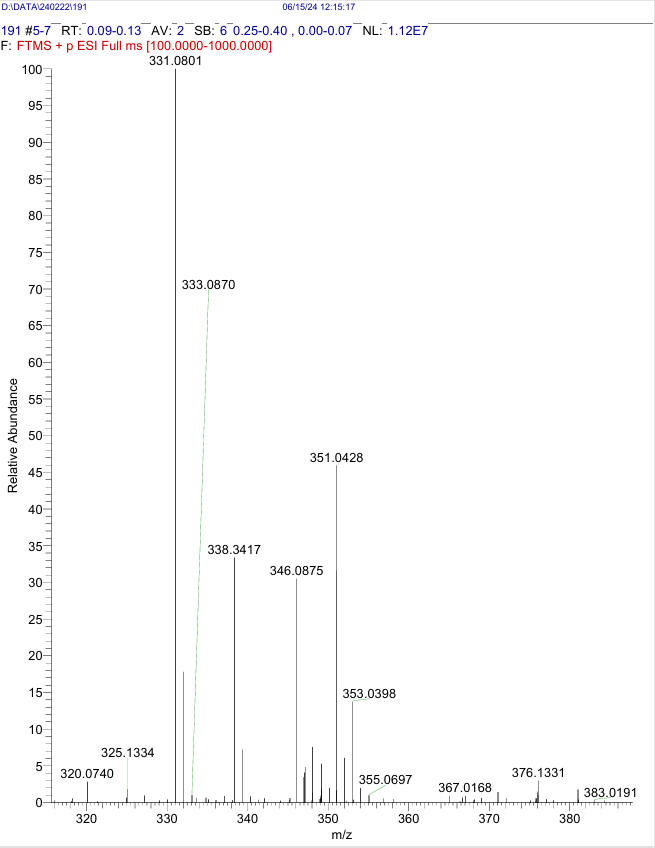


**Figure S6**. HRMS Spectrum of compound *2-(2-chlorophenoxy)cyclohexyl prop-2-yn-1-yl sulfite* (**5.02**)
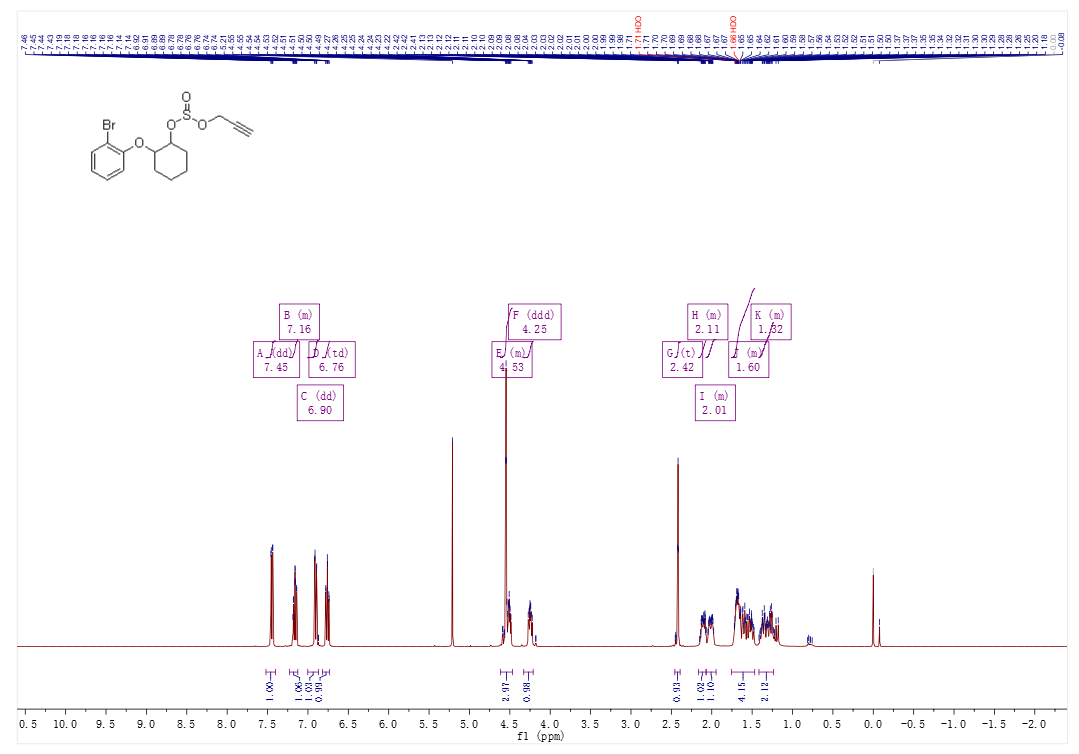


**Figure S7**. The ^1^H NMR spectrum of compound *2-(2-bromophenoxy)cyclohexyl prop-2-yn-1-yl sulfite* (**5.03**)


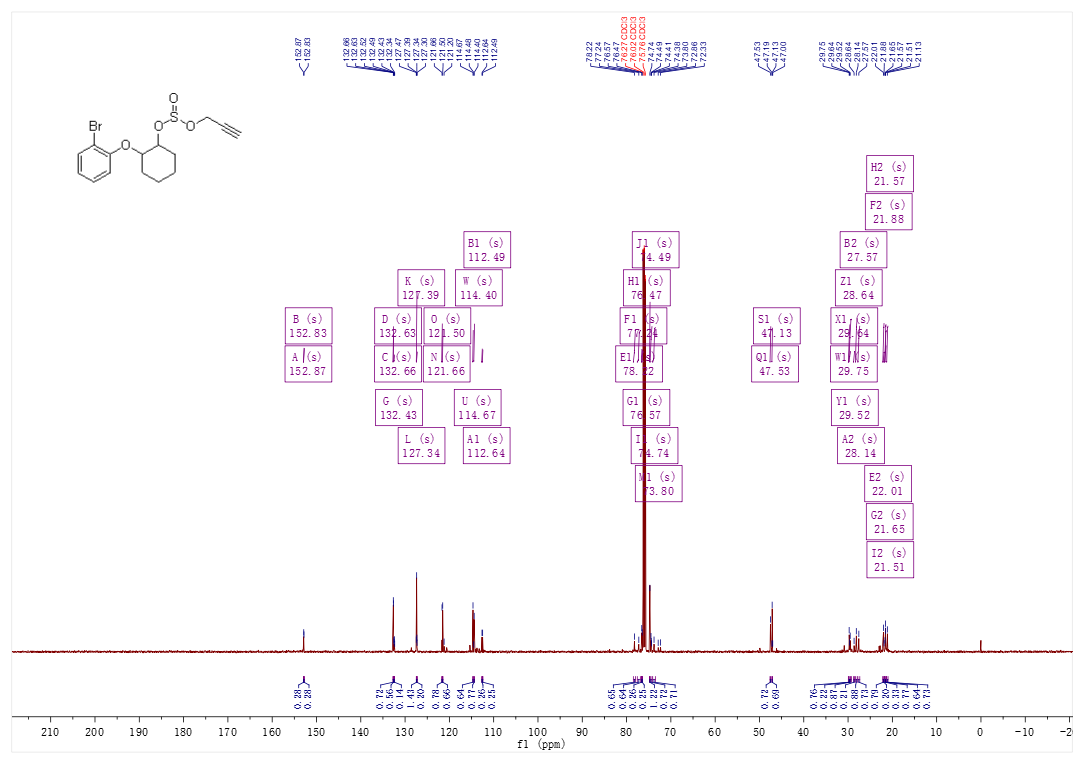


**Figure S8**. The ^13^C NMR spectrum of compound *2-(2-bromophenoxy)cyclohexyl prop-2-yn-1-yl sulfite* (**5.03**)


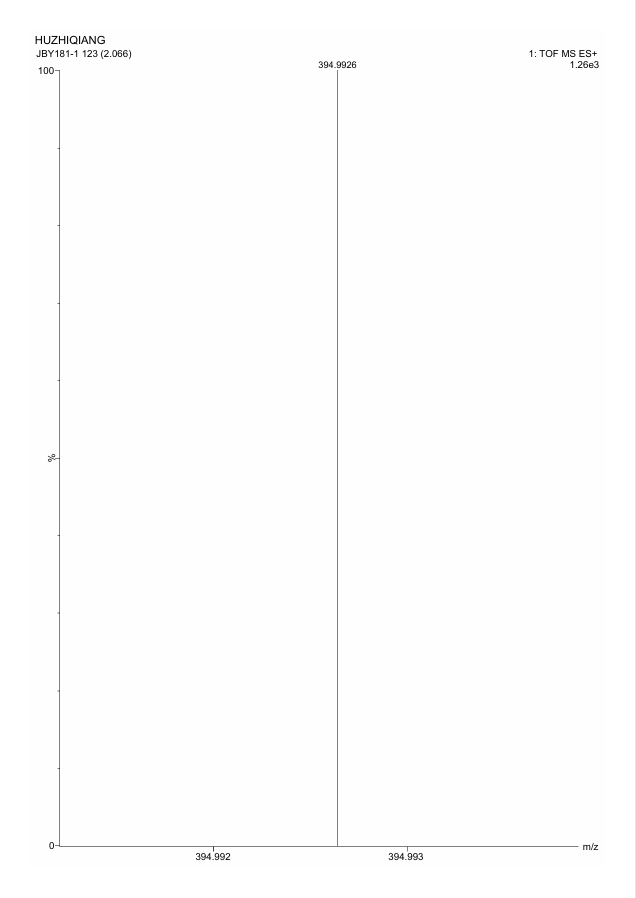


**Figure S9**. HRMS Spectrum of compound *2-(2-bromophenoxy)cyclohexyl prop-2-yn-1-yl sulfite* (**5.03**)
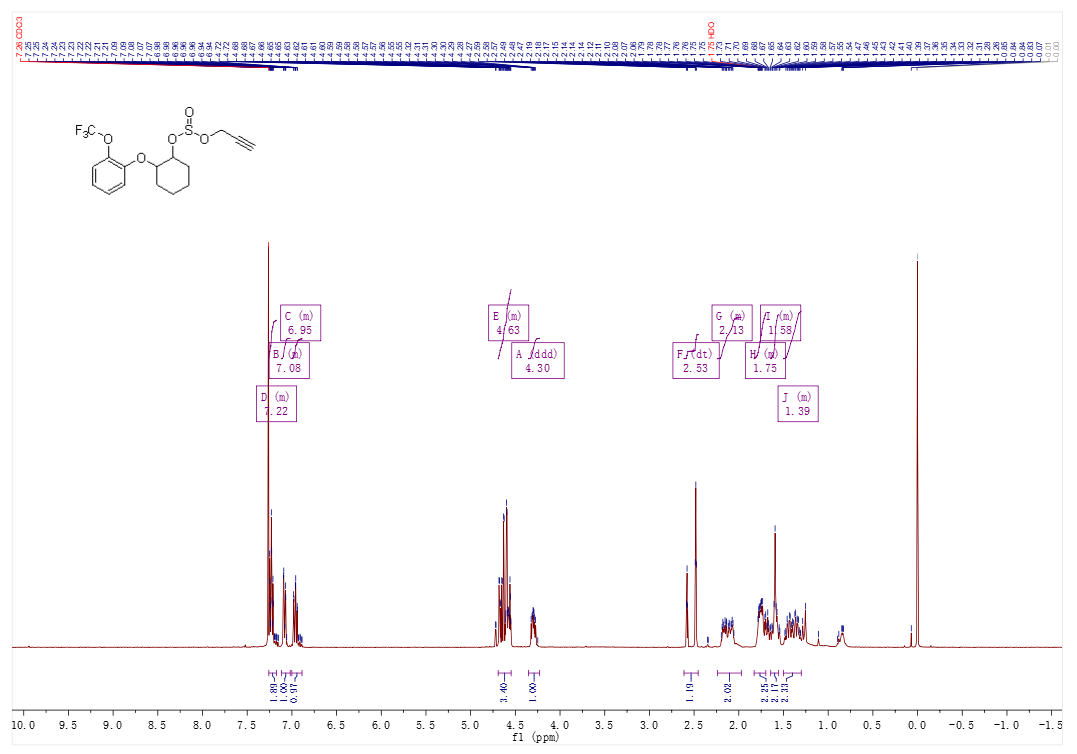


**Figure S10**. The ^1^H NMR spectrum of compound *prop-2-yn-1-yl (2-(2-(trifluoromethoxy)phenoxy)cyclohexyl) sulfite* (**5.04**)


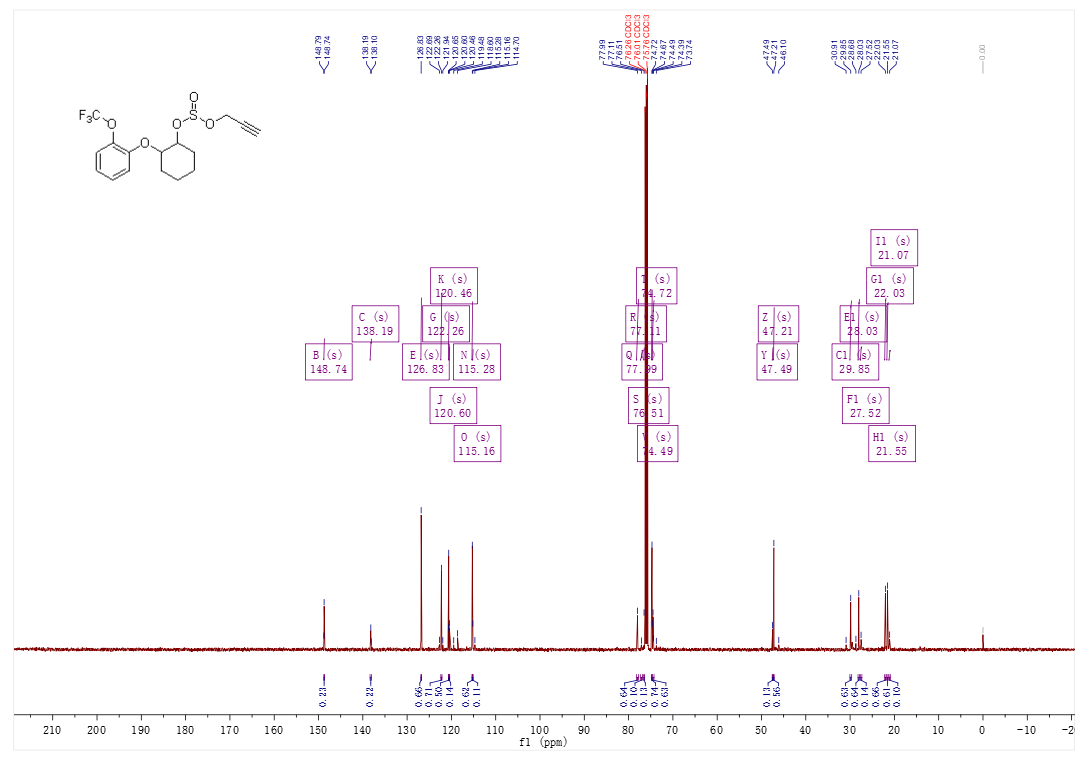


**Figure S11**. The ^13^C NMR spectrum of compound *prop-2-yn-1-yl (2-(2-(trifluoromethoxy)phenoxy)cyclohexyl) sulfite* (**5.04**)


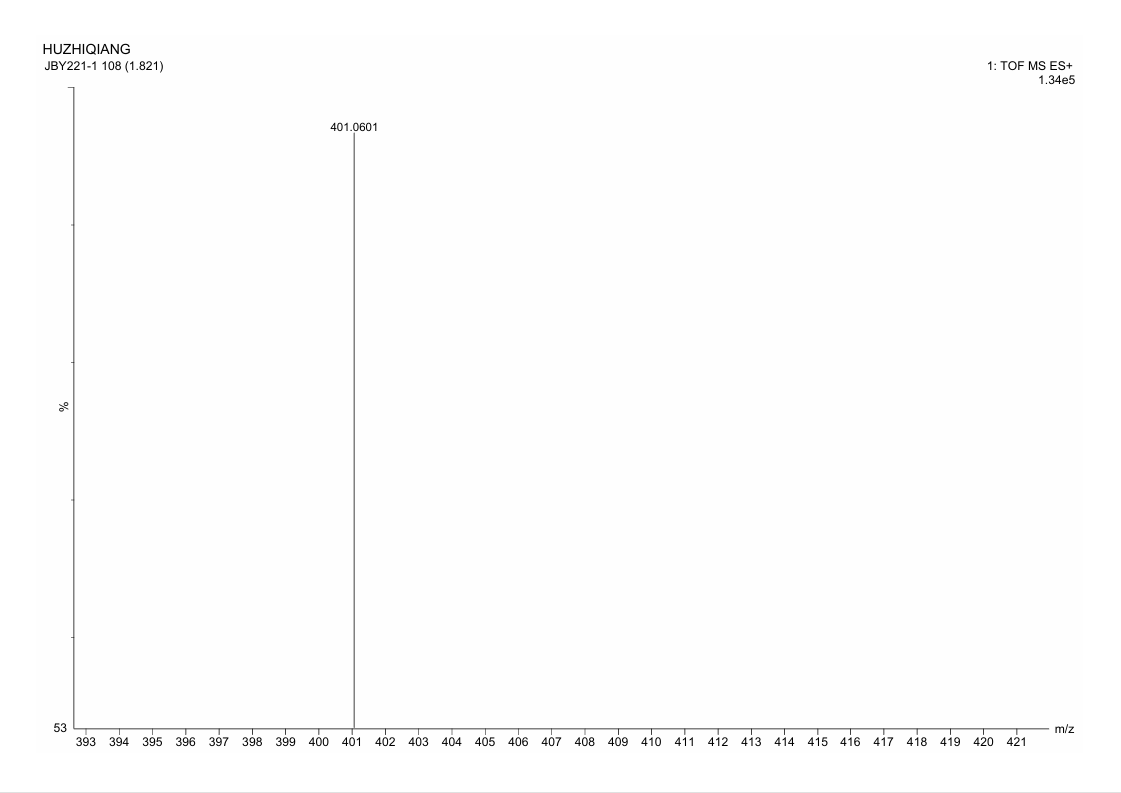


**Figure S12**. HRMS Spectrum of compound *prop-2-yn-1-yl (2-(2-(trifluoromethoxy)phenoxy)cyclohexyl) sulfite* (**5.04**)


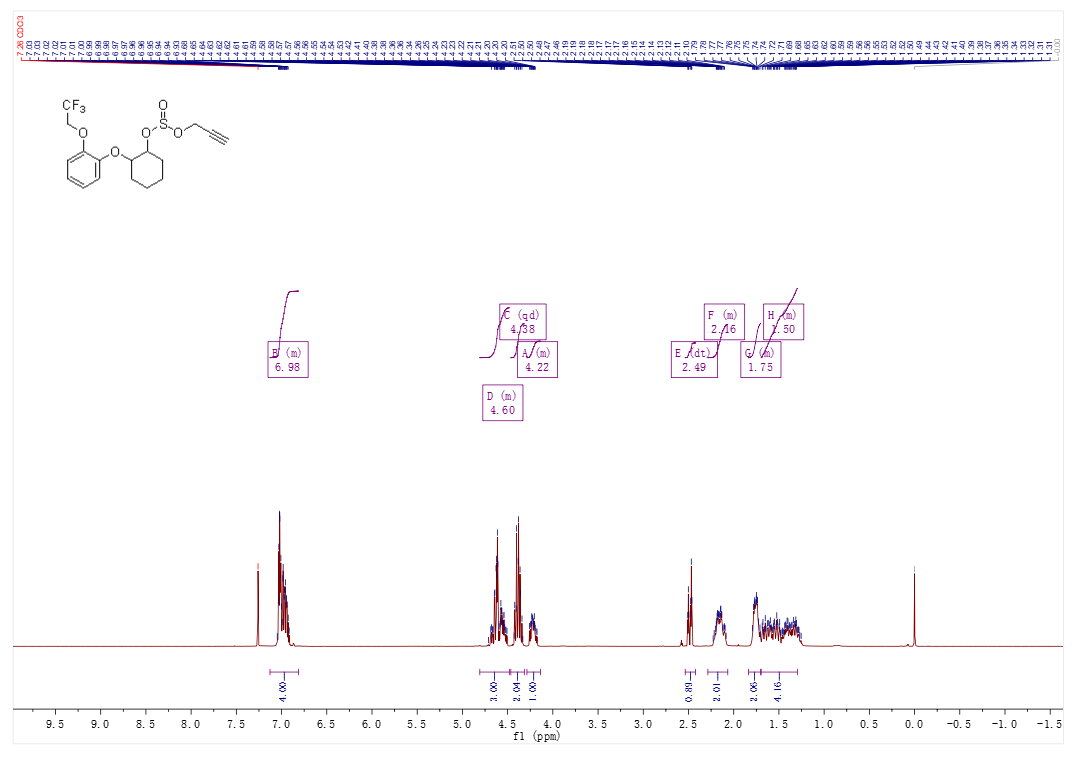


**Figure S13**. The ^1^H NMR spectrum of compound *prop-2-yn-1-yl (2-(2-(2,2,2-trifluoroethoxy)phenoxy)cyclohexyl) sulfite* (**5.05**)


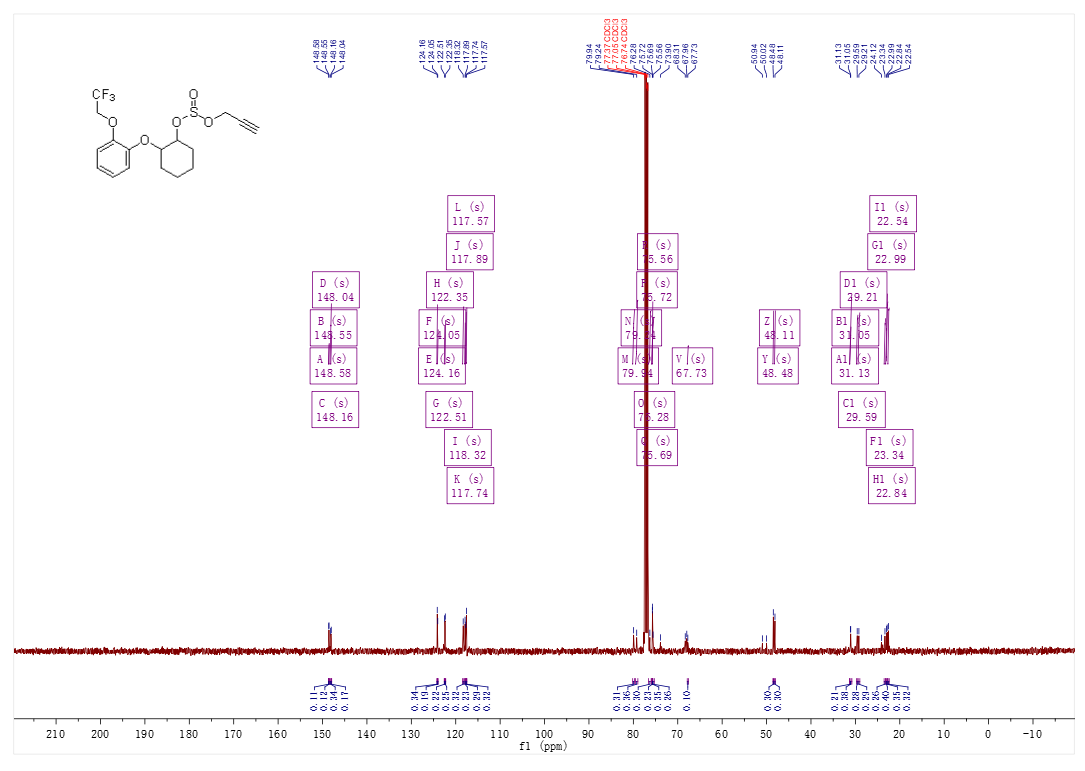


**Figure S14**. The ^13^C NMR spectrum of compound *prop-2-yn-1-yl (2-(2-(2,2,2-trifluoroethoxy)phenoxy)cyclohexyl) sulfite* (**5.05**)


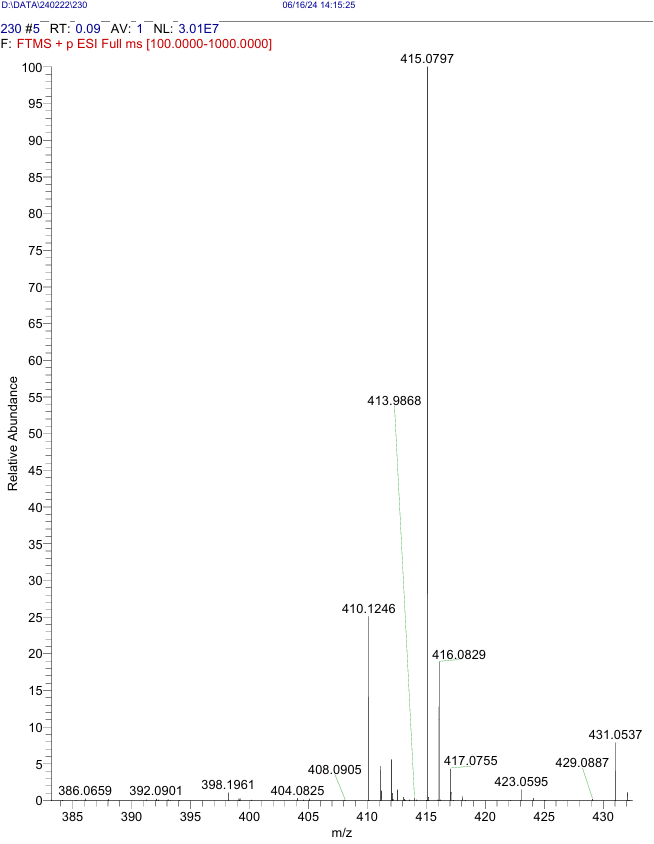


**Figure S15**. HRMS Spectrum of compound *prop-2-yn-1-yl (2-(2-(2,2,2-trifluoroethoxy)phenoxy)cyclohexyl) sulfite* (**5.05**)
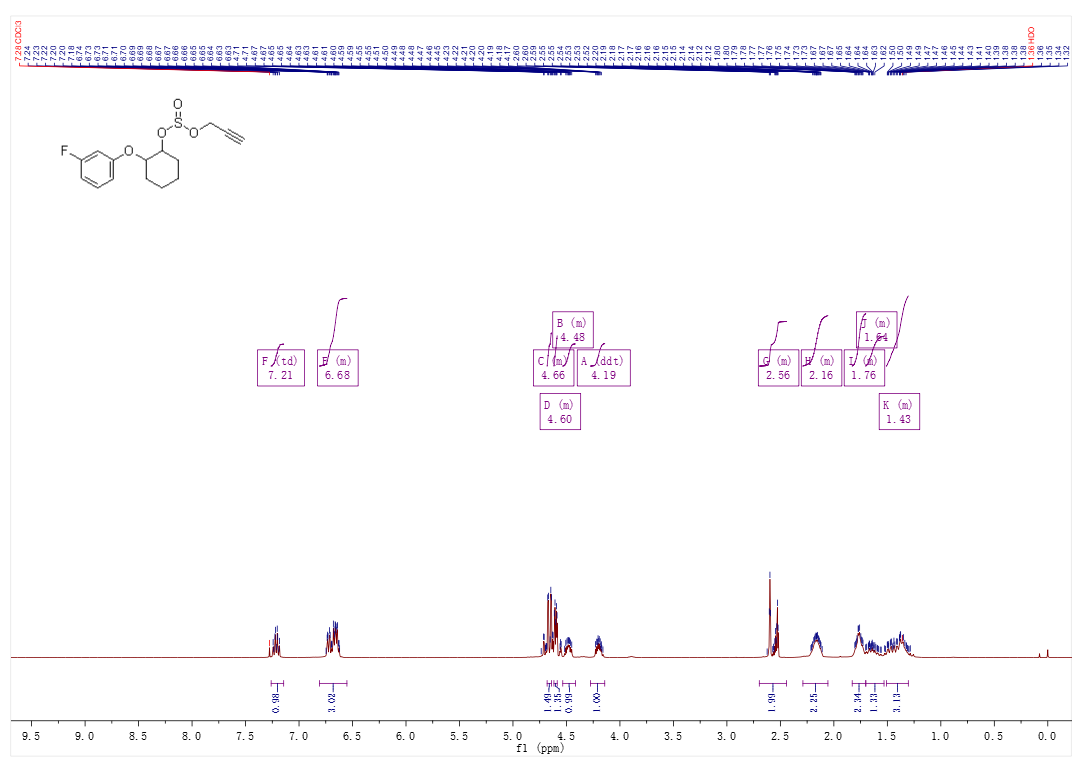


**Figure S16**. The ^1^H NMR spectrum of compound *2-(3-fluorophenoxy)cyclohexyl prop-2-yn-1-yl sulfite* (**5.06**)


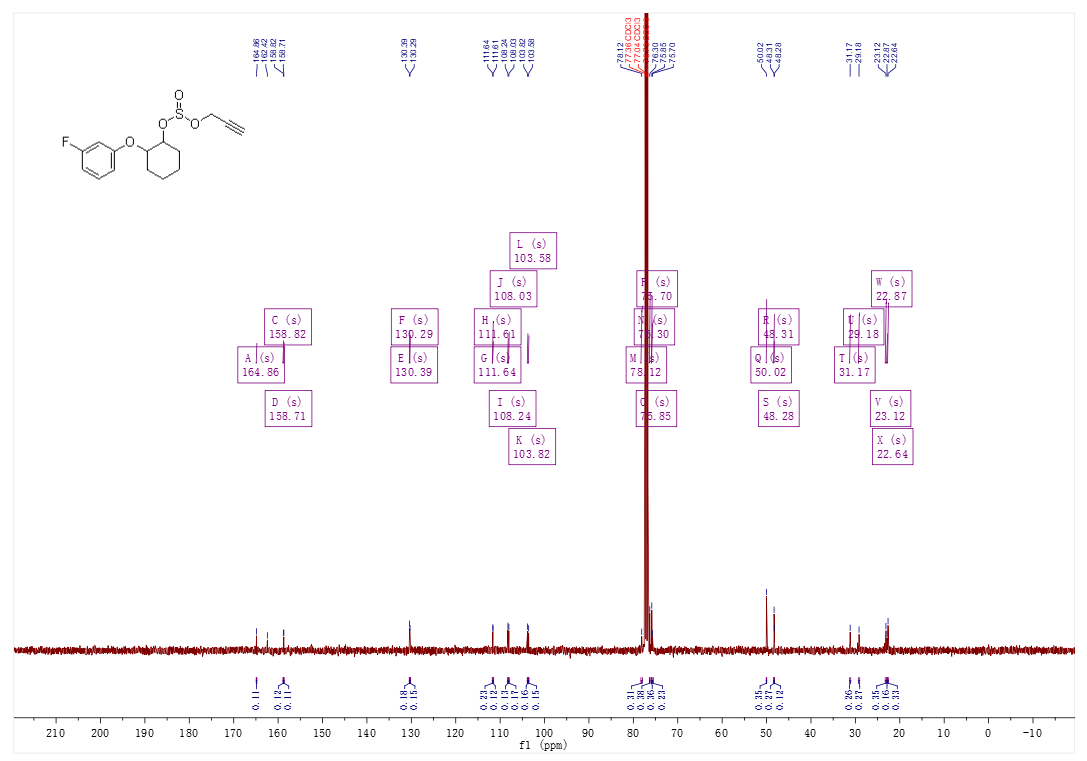


**Figure S17**. The ^13^C NMR spectrum of compound *2-(3-fluorophenoxy)cyclohexyl prop-2-yn-1-yl sulfite* (**5.06**)


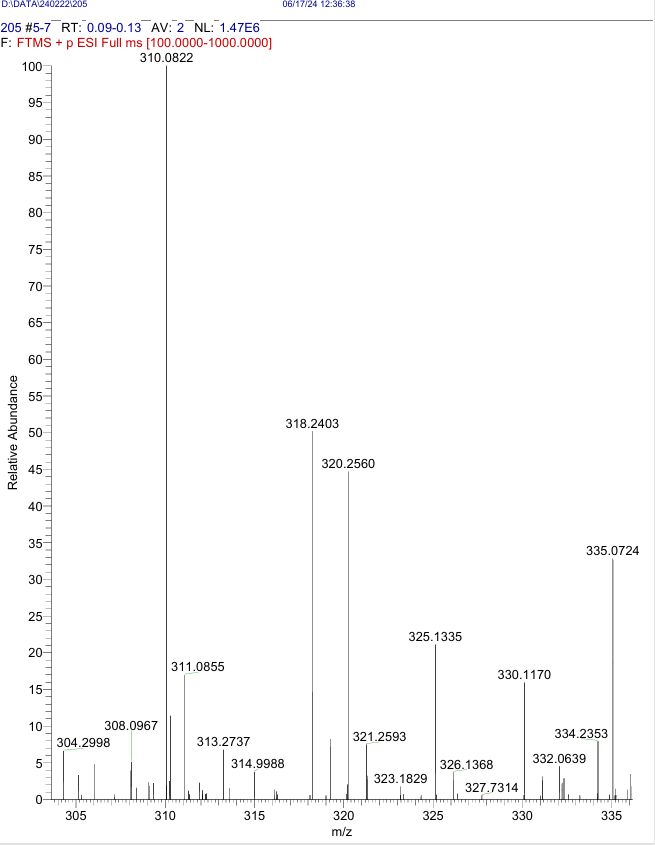


**Figure S18**. HRMS Spectrum of compound *2-(3-fluorophenoxy)cyclohexyl prop-2-yn-1-yl sulfite* (**5.06**)
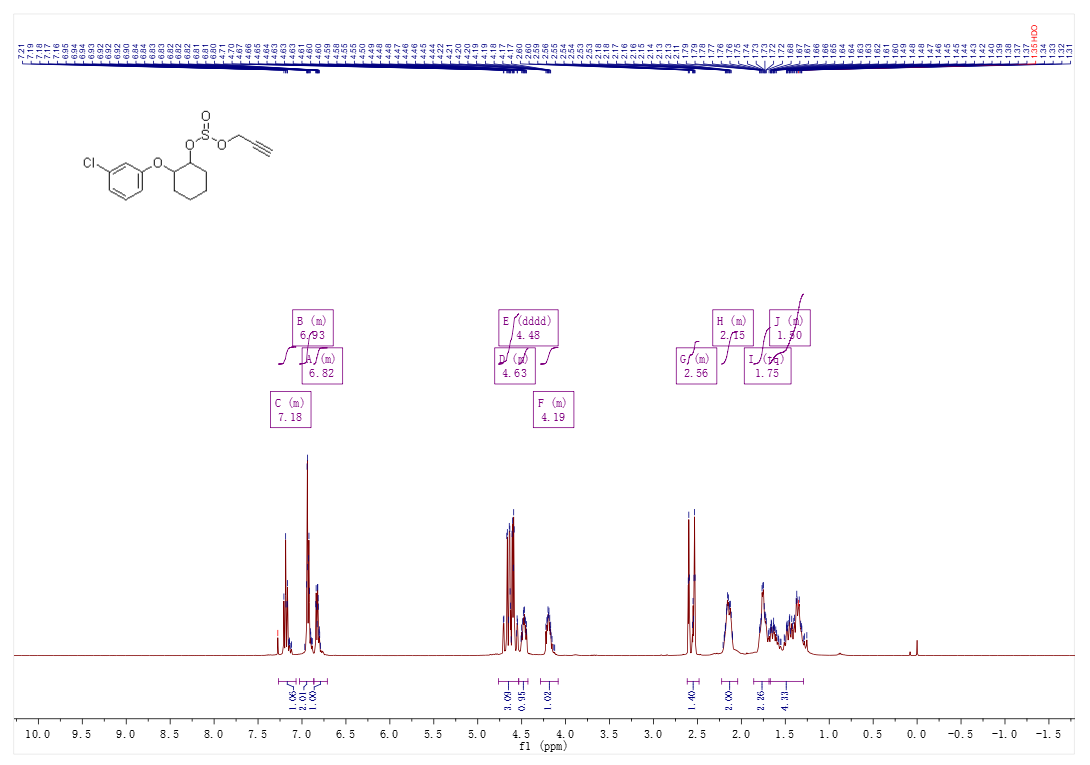


**Figure S19**. The ^1^H NMR spectrum of compound *2-(3-chlorophenoxy)cyclohexyl prop-2-yn-1-yl sulfite* (**5.07**)


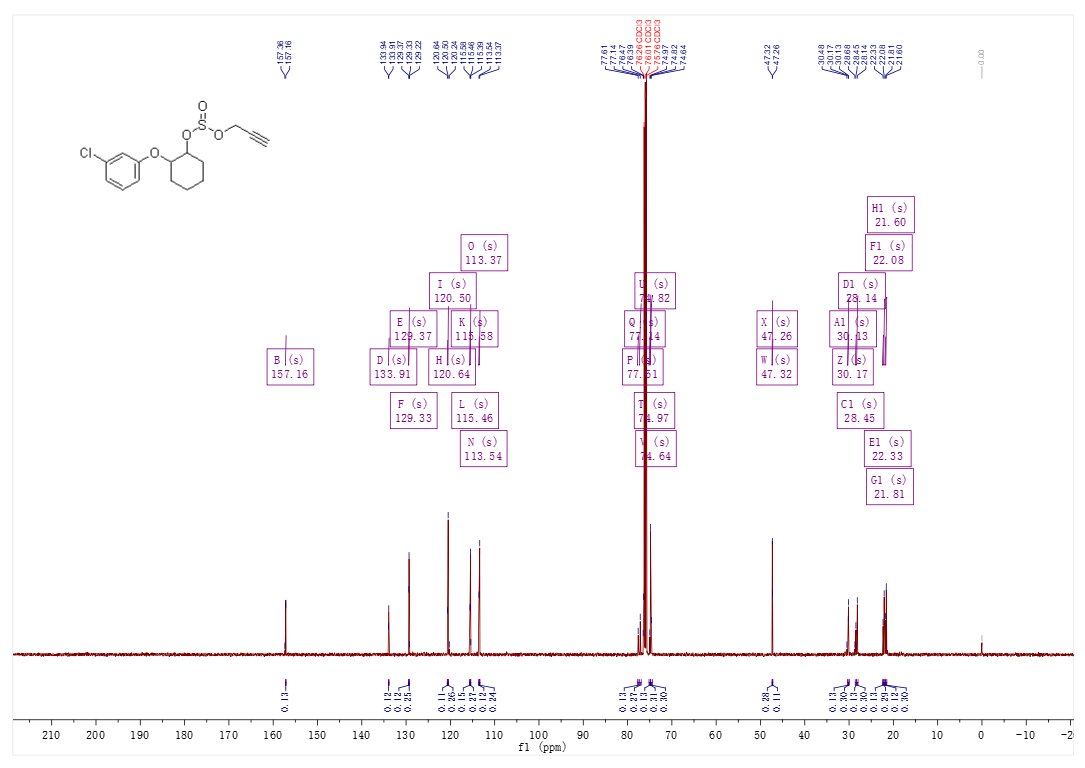


**Figure S20**. The ^13^C NMR spectrum of compound *2-(3-chlorophenoxy)cyclohexyl prop-2-yn-1-yl sulfite* (**5.07**)


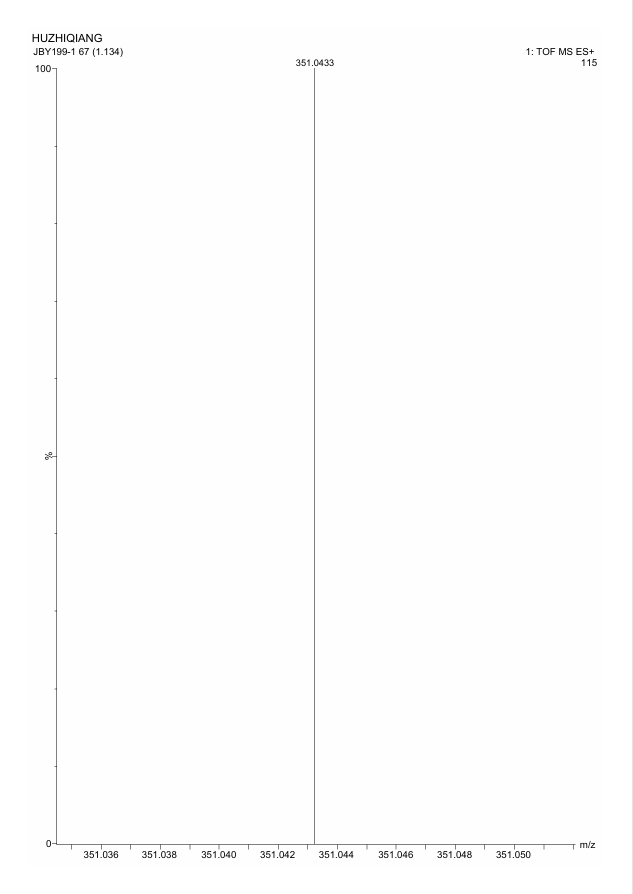


**Figure S21**. HRMS Spectrum of compound *2-(3-chlorophenoxy)cyclohexyl prop-2-yn-1-yl sulfite* (**5.07**)
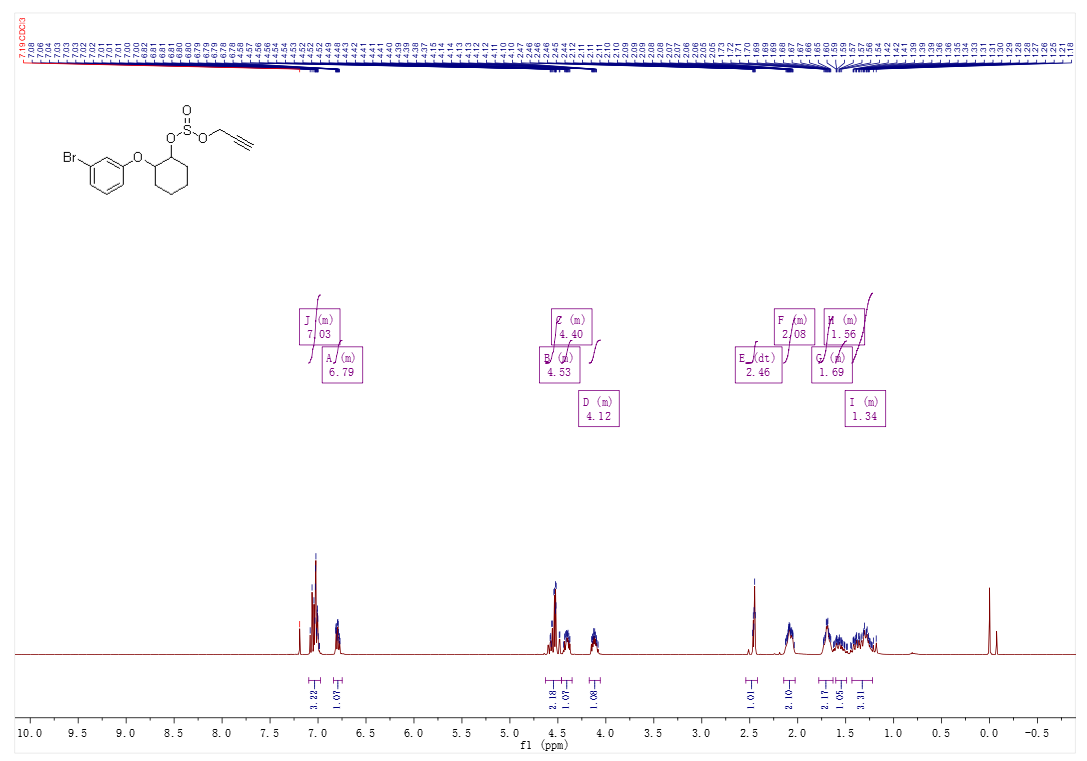


**Figure S22**. The ^1^H NMR spectrum of compound *2-(3-bromophenoxy)cyclohexyl prop-2-yn-1-yl sulfite* (**5.08**)


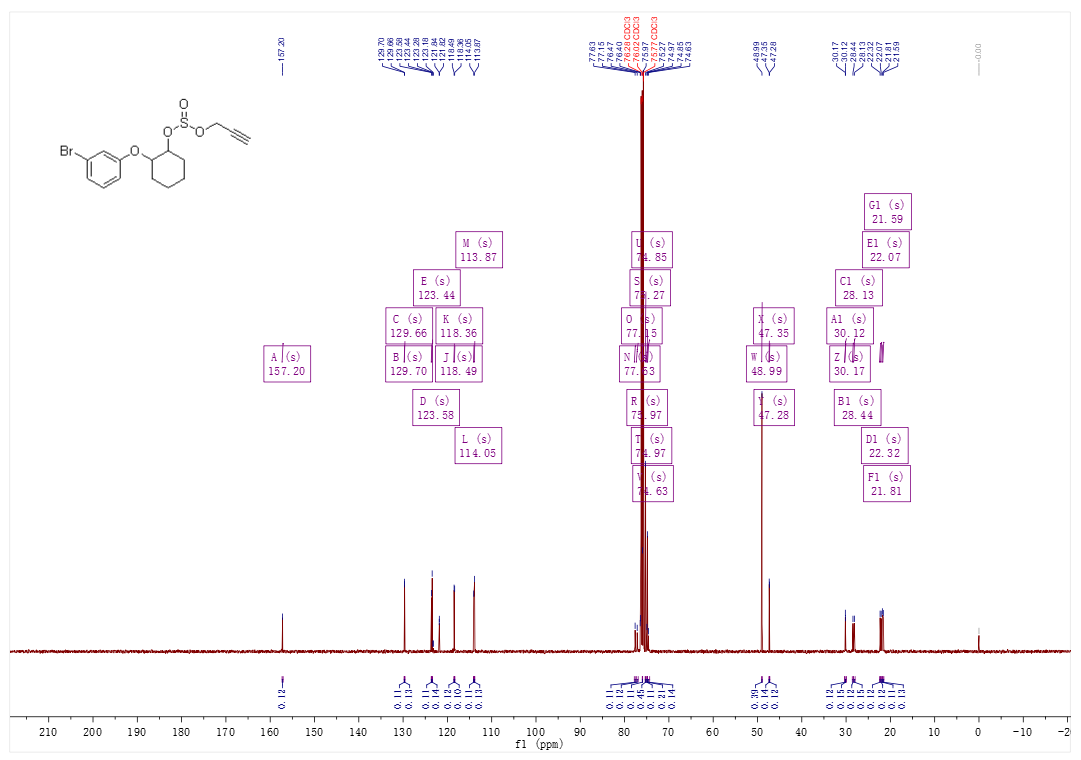


**Figure S23**. The ^13^C NMR spectrum of compound *2-(3-bromophenoxy)cyclohexyl prop-2-yn-1-yl sulfite* (**5.08**)


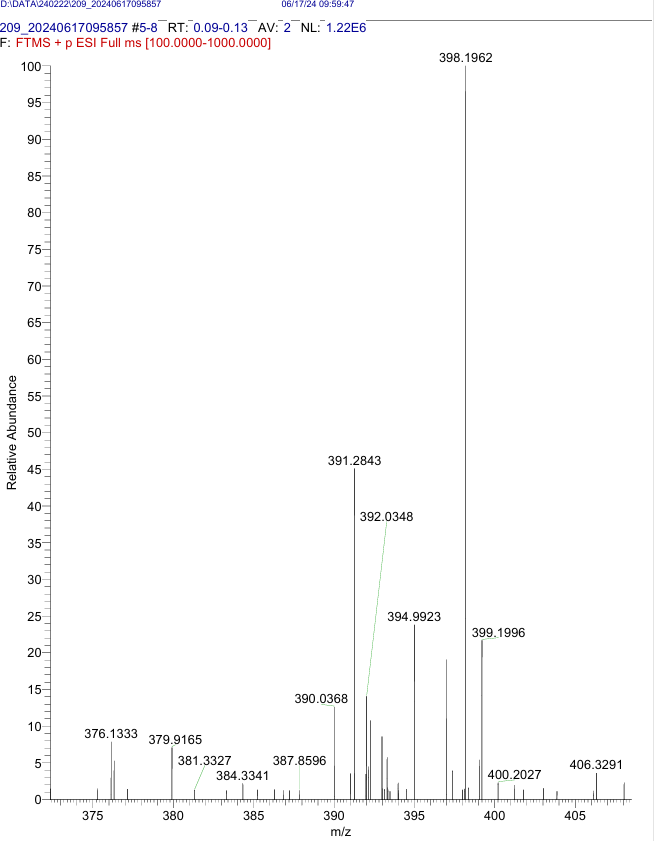


**Figure S24**. HRMS Spectrum of compound *2-(3-bromophenoxy)cyclohexyl prop-2-yn-1-yl sulfite* (**5.08**)
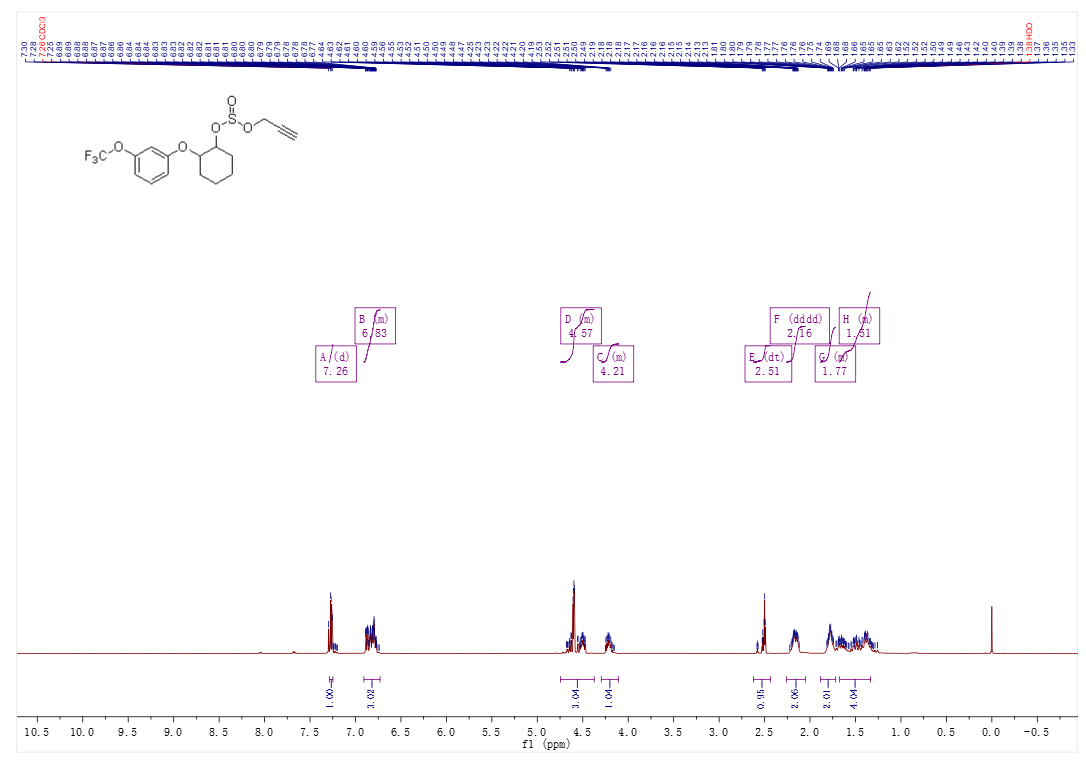


**Figure S25**. The ^1^H NMR spectrum of compound *prop-2-yn-1-yl (2-(3-(trifluoromethoxy)phenoxy)cyclohexyl) sulfite* (**5.09**)


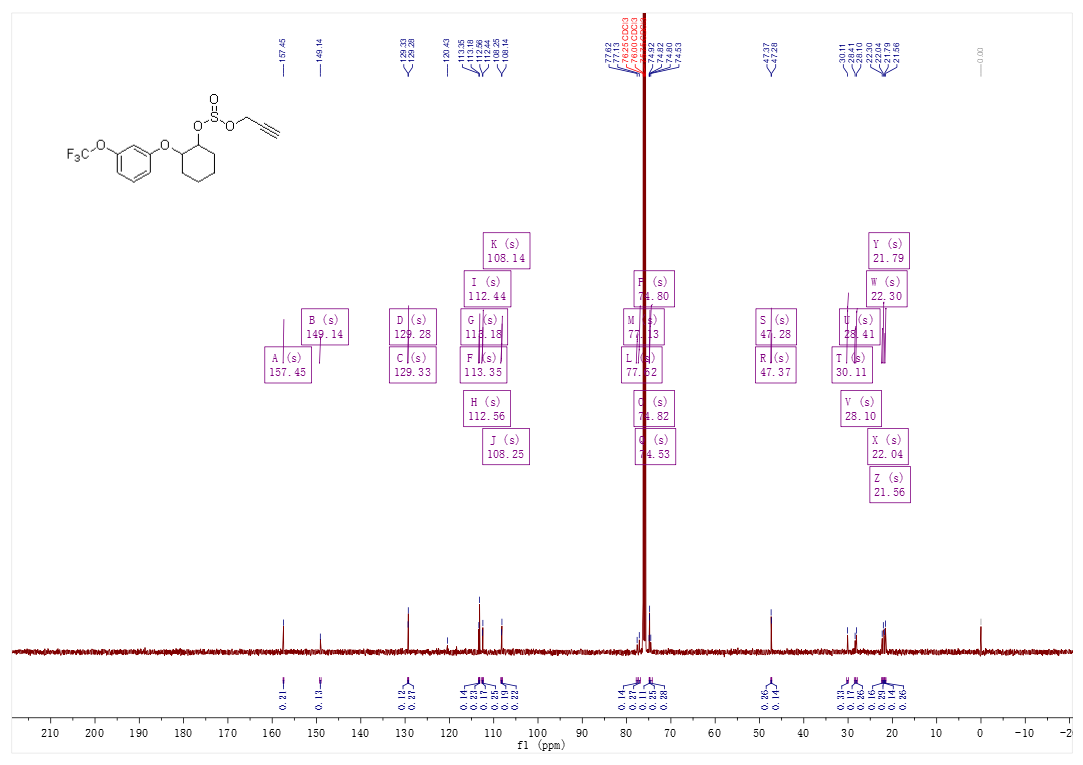


**Figure S26**. The ^13^C NMR spectrum of compound *prop-2-yn-1-yl (2-(3-(trifluoromethoxy)phenoxy)cyclohexyl) sulfite* (**5.09**)


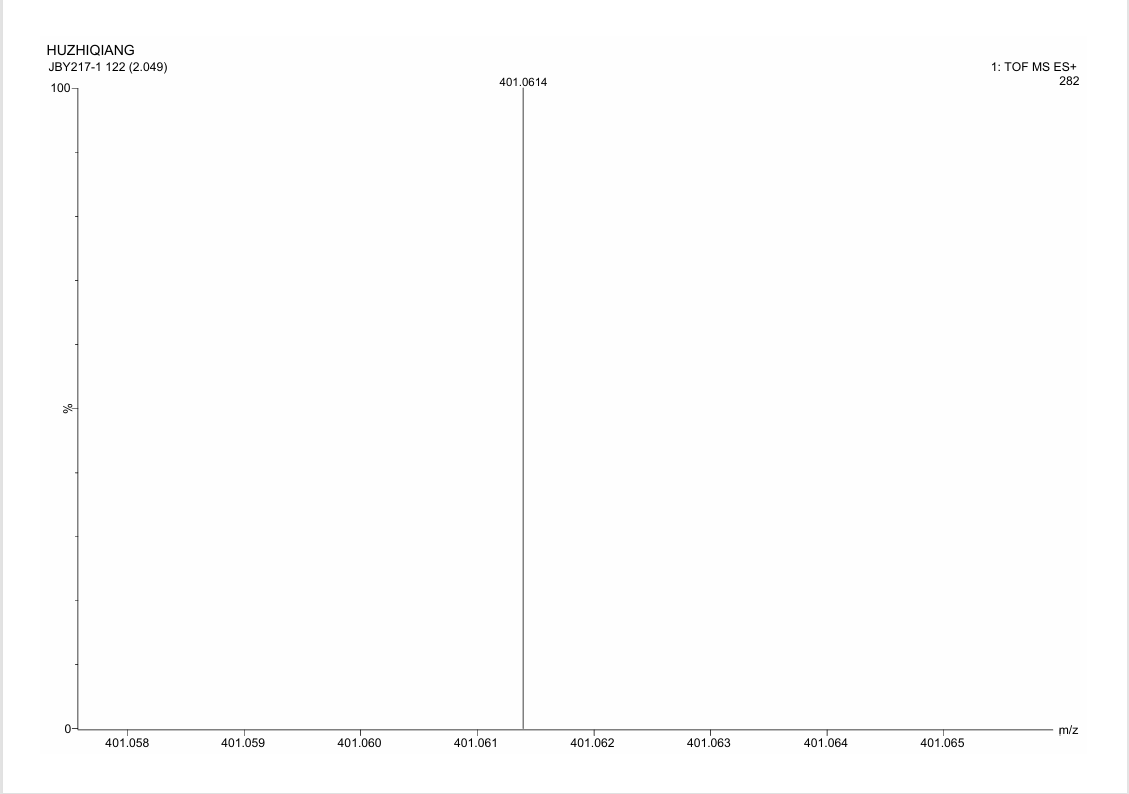


**Figure S27**. HRMS Spectrum of compound *prop-2-yn-1-yl (2-(3-(trifluoromethoxy)phenoxy)cyclohexyl) sulfite* (**5.09**)


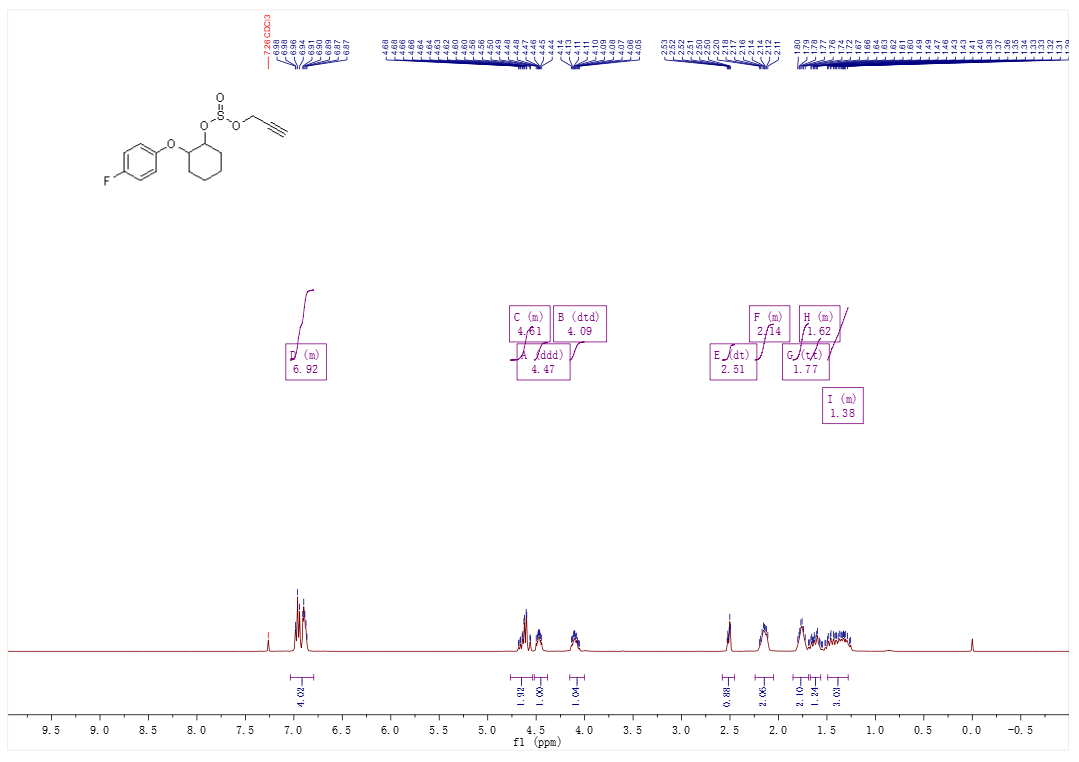


**Figure S28**. The ^1^H NMR spectrum of compound *2-(4-fluorophenoxy)cyclohexyl prop-2-yn-1-yl sulfite* (**5.10**)


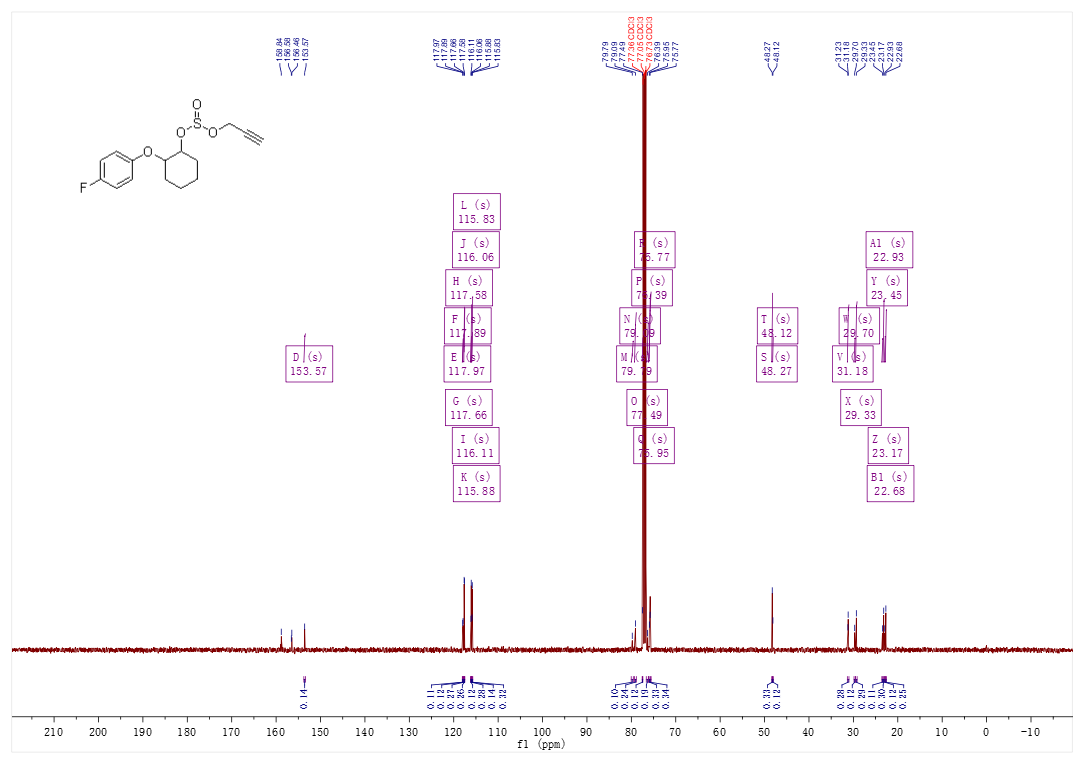


**Figure S29**. The ^13^C NMR spectrum of compound *2-(4-fluorophenoxy)cyclohexyl prop-2-yn-1-yl sulfite* (**5.10**)


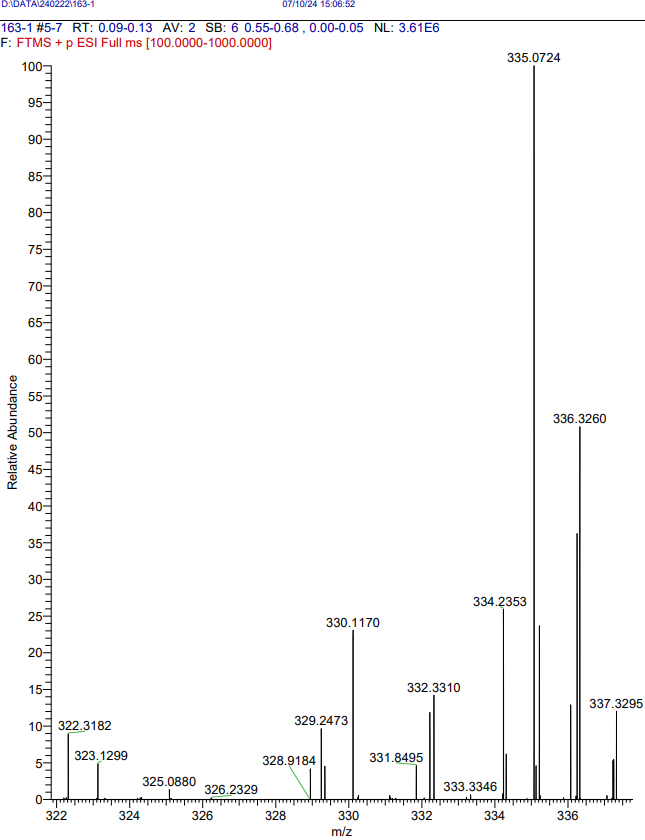


**Figure S30**. HRMS Spectrum of compound *2-(4-fluorophenoxy)cyclohexyl prop-2-yn-1-yl sulfite* (**5.10**)


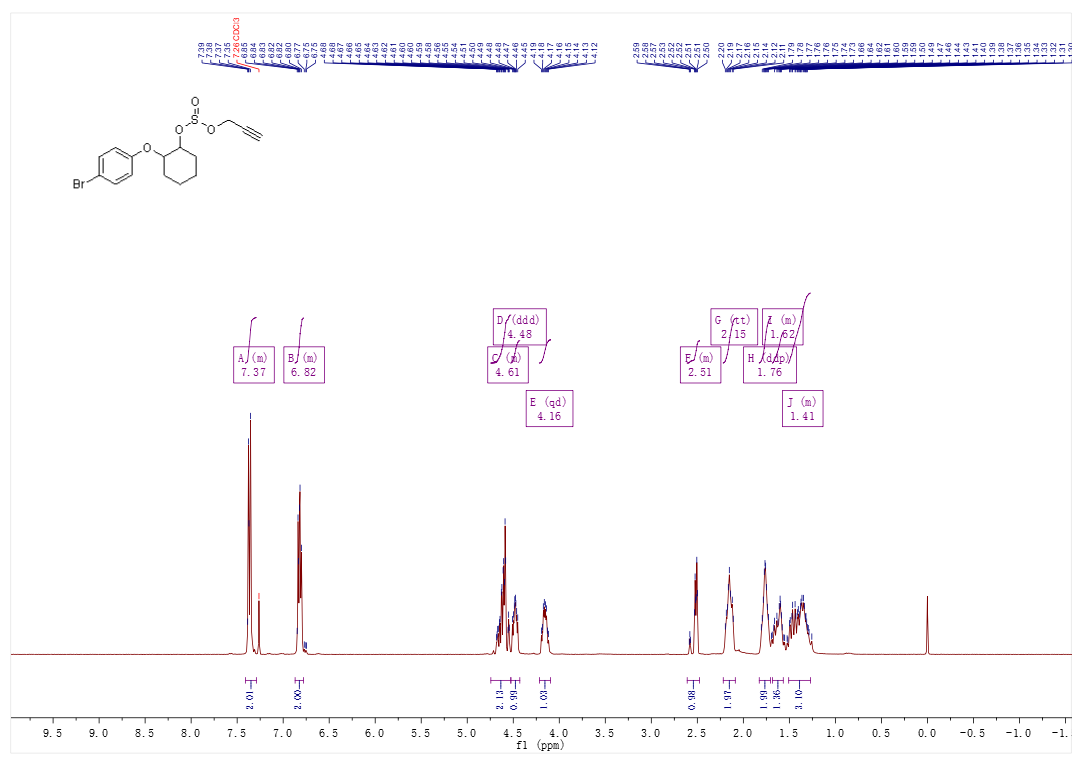


**Figure S31**. The ^1^H NMR spectrum of compound *2-(4-bromophenoxy)cyclohexyl prop-2-yn-1-yl sulfite* (**5.11**)


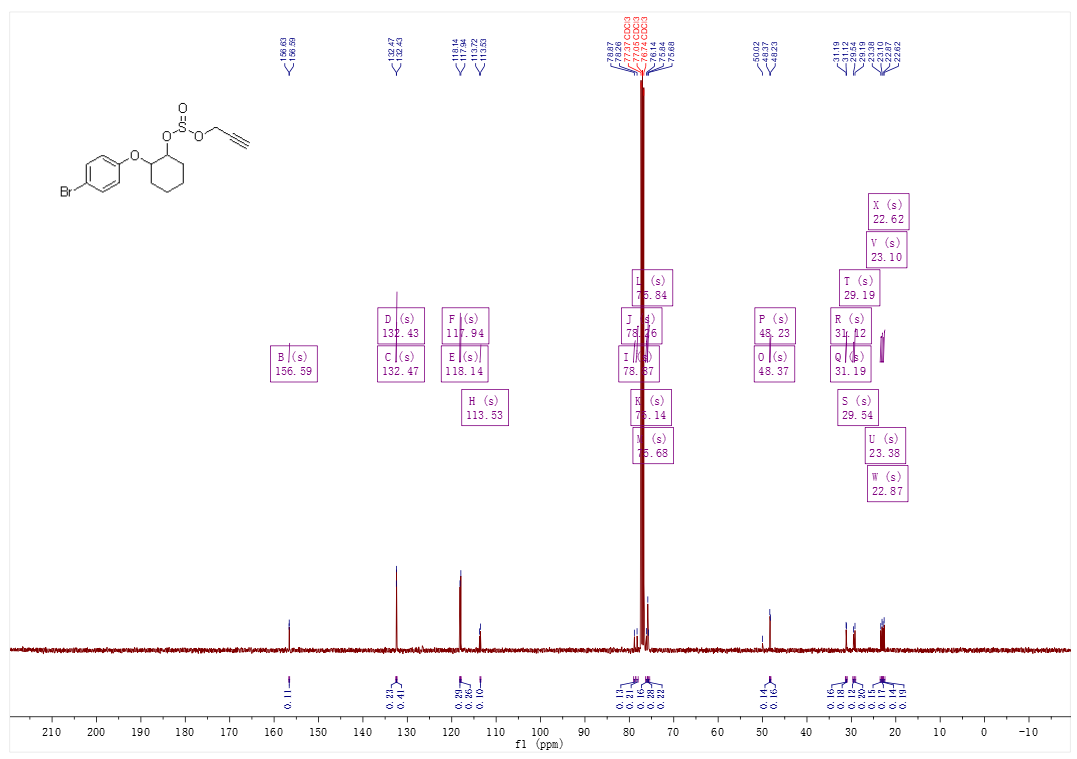


**Figure S32**. The ^13^C NMR spectrum of compound *2-(4-bromophenoxy)cyclohexyl prop-2-yn-1-yl sulfite* (**5.11**)


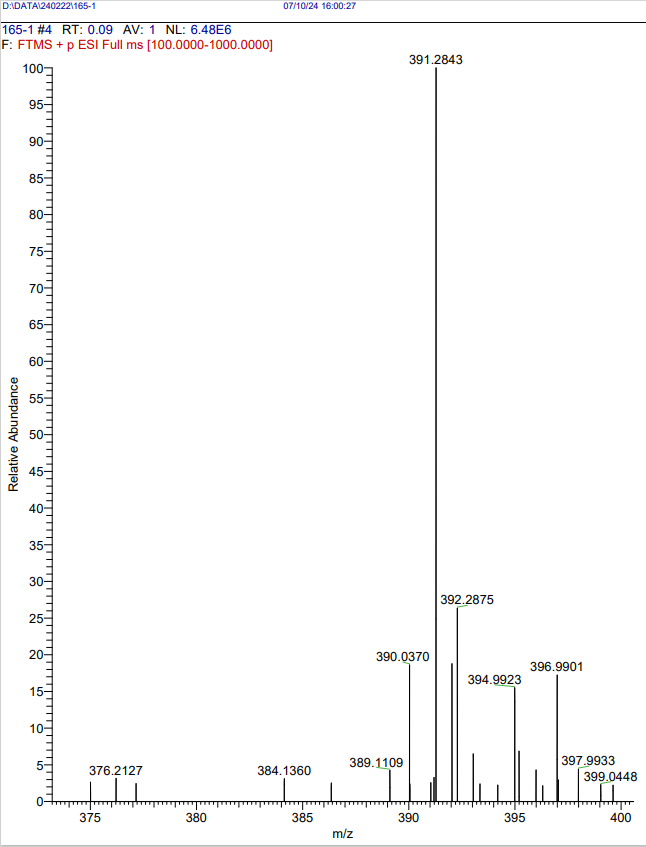


**Figure S33**. HRMS Spectrum of compound *2-(4-bromophenoxy)cyclohexyl prop-2-yn-1-yl sulfite* (**5.11**)


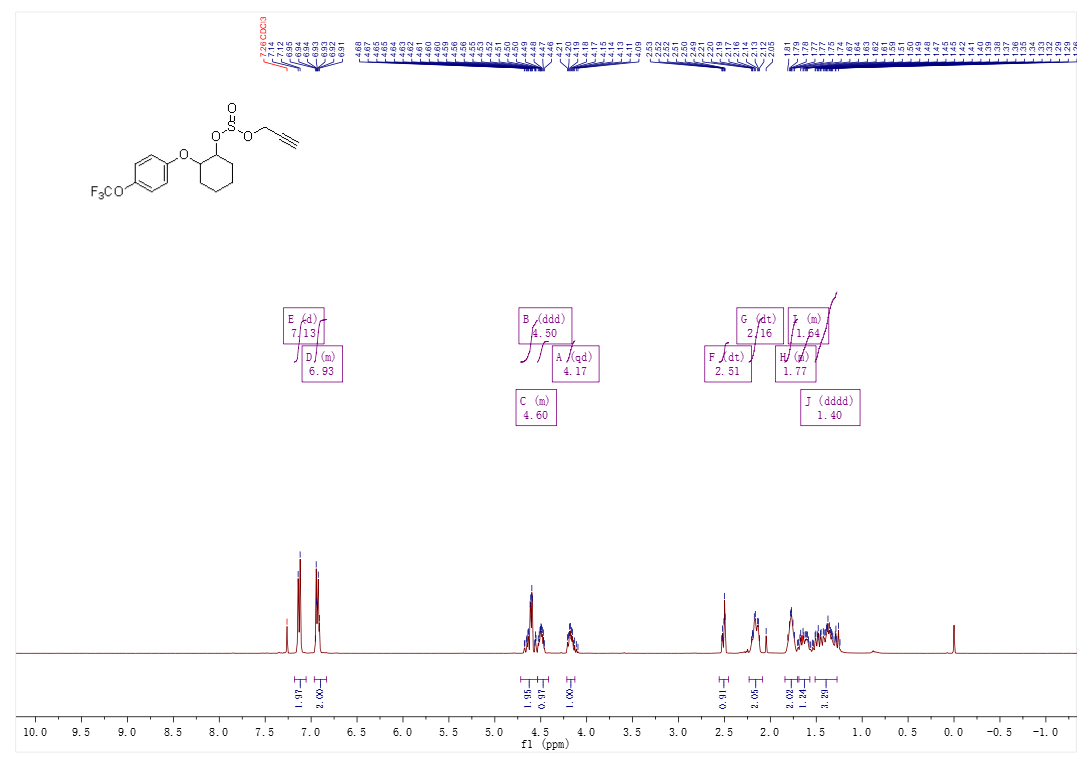


**Figure S34**. The ^1^H NMR spectrum of compound *prop-2-yn-1-yl (2-(4-(trifluoromethoxy)phenoxy)cyclohexyl) sulfite* (**5.12**)


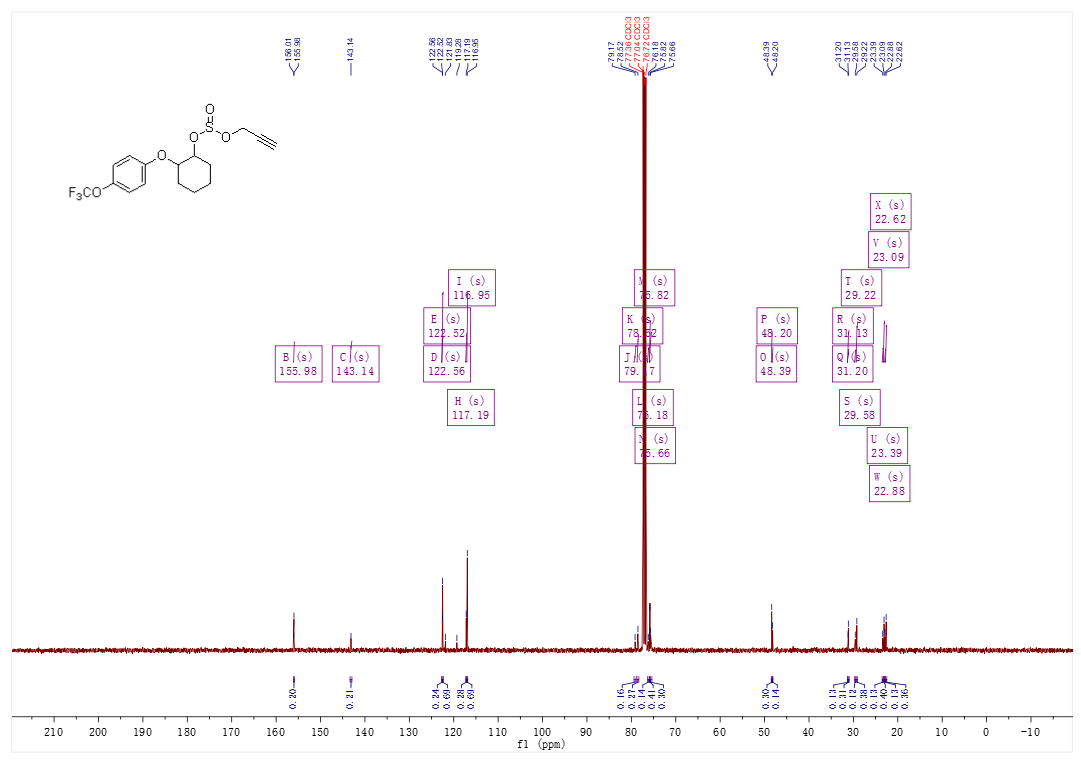


**Figure S35**. The ^13^C NMR spectrum of compound *prop-2-yn-1-yl (2-(4-(trifluoromethoxy)phenoxy)cyclohexyl) sulfite* (**5.12**)


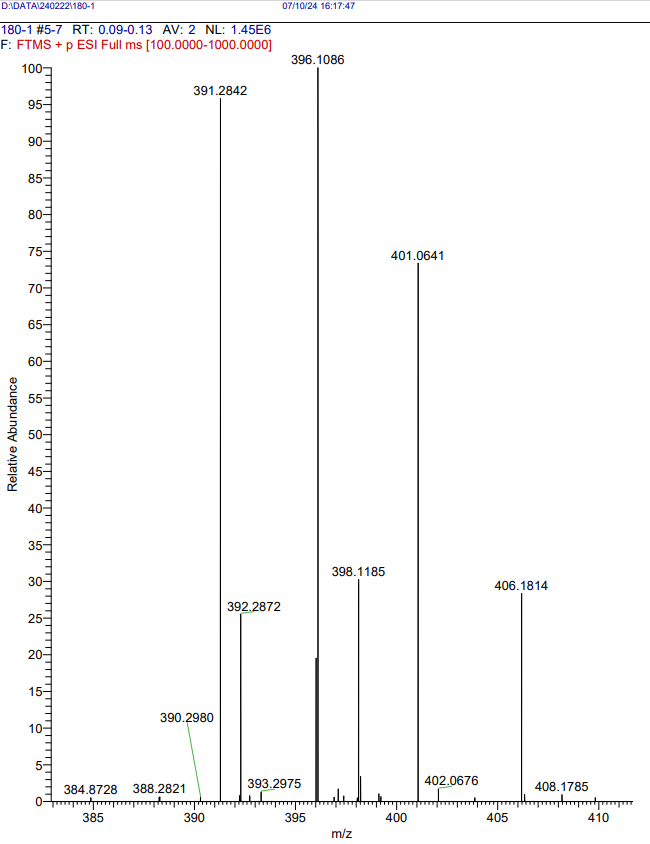


**Figure S36**. HRMS Spectrum of compound *prop-2-yn-1-yl (2-(4-(trifluoromethoxy)phenoxy)cyclohexyl) sulfite* (**5.12**)
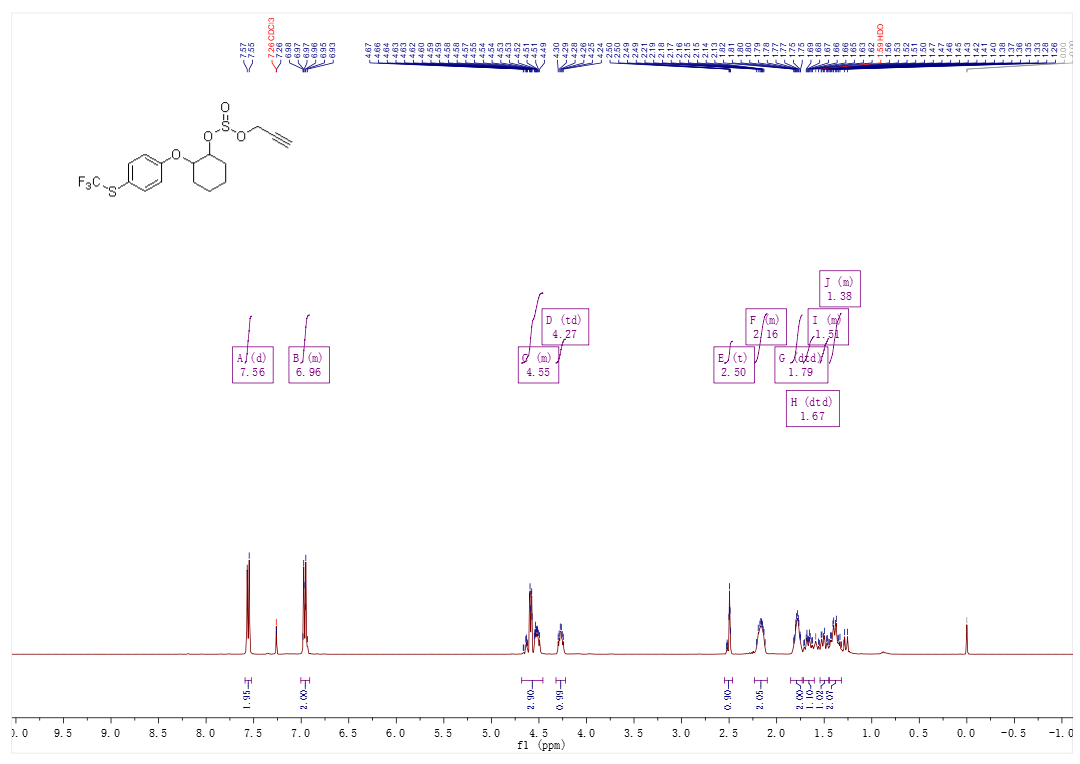


**Figure S37**. The ^1^H NMR spectrum of compound *prop-2-yn-1-yl (2-(4-((trifluoromethyl)thio)phenoxy)cyclohexyl) sulfite* (**5.13**)


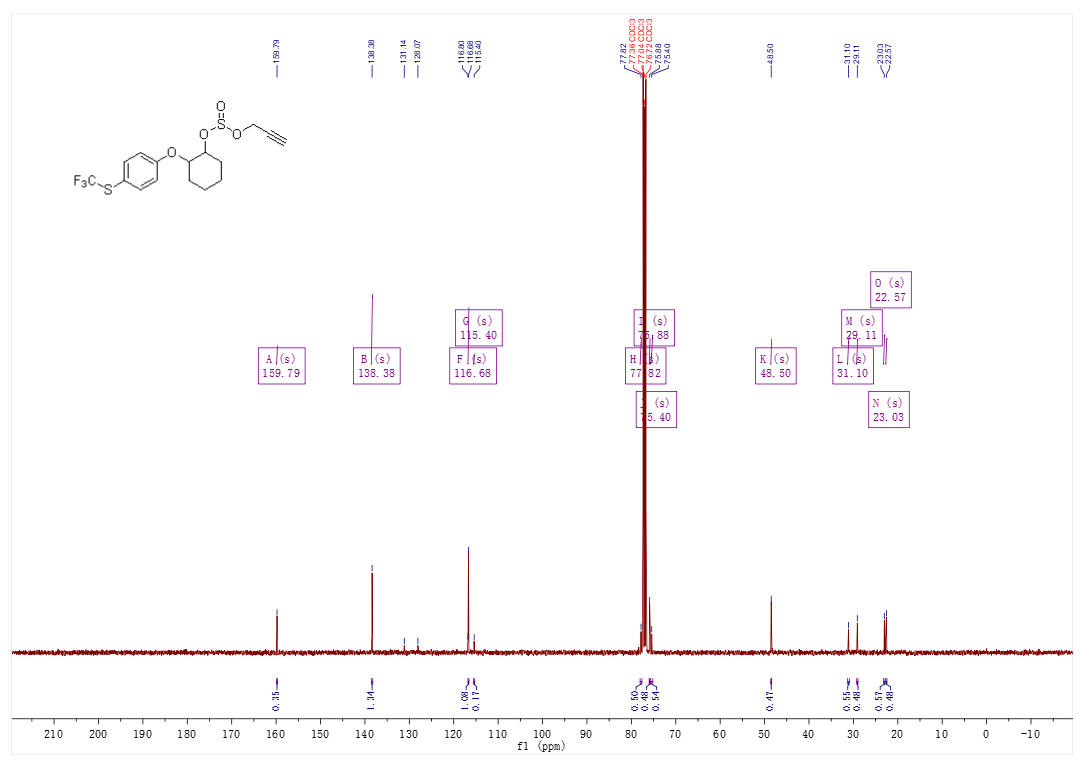


**Figure S38**. The ^13^C NMR spectrum of compound *prop-2-yn-1-yl (2-(4-((trifluoromethyl)thio)phenoxy)cyclohexyl) sulfite* (**5.13**)


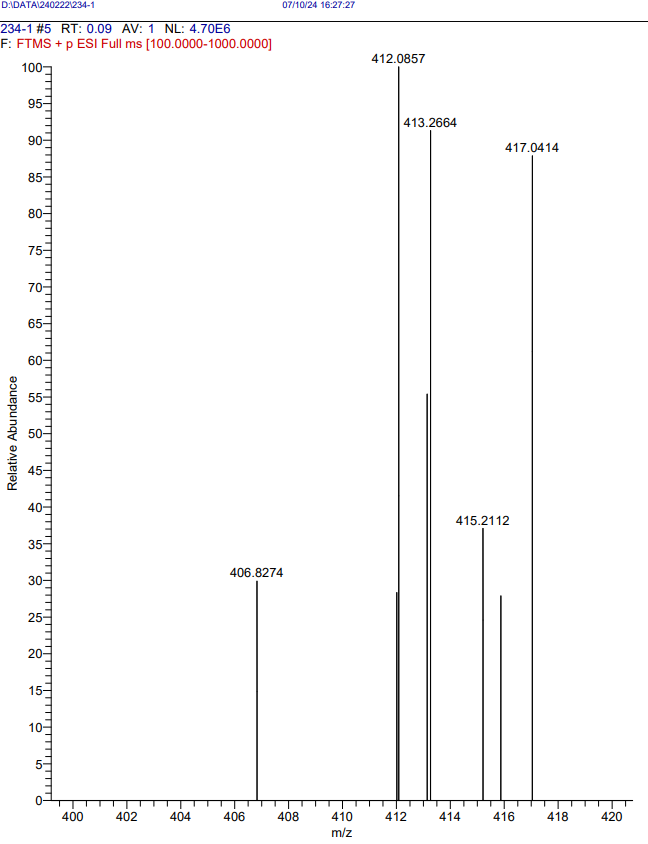


**Figure S39**. HRMS Spectrum of compound *prop-2-yn-1-yl (2-(4-((trifluoromethyl)thio)phenoxy)cyclohexyl) sulfite* (**5.13**)


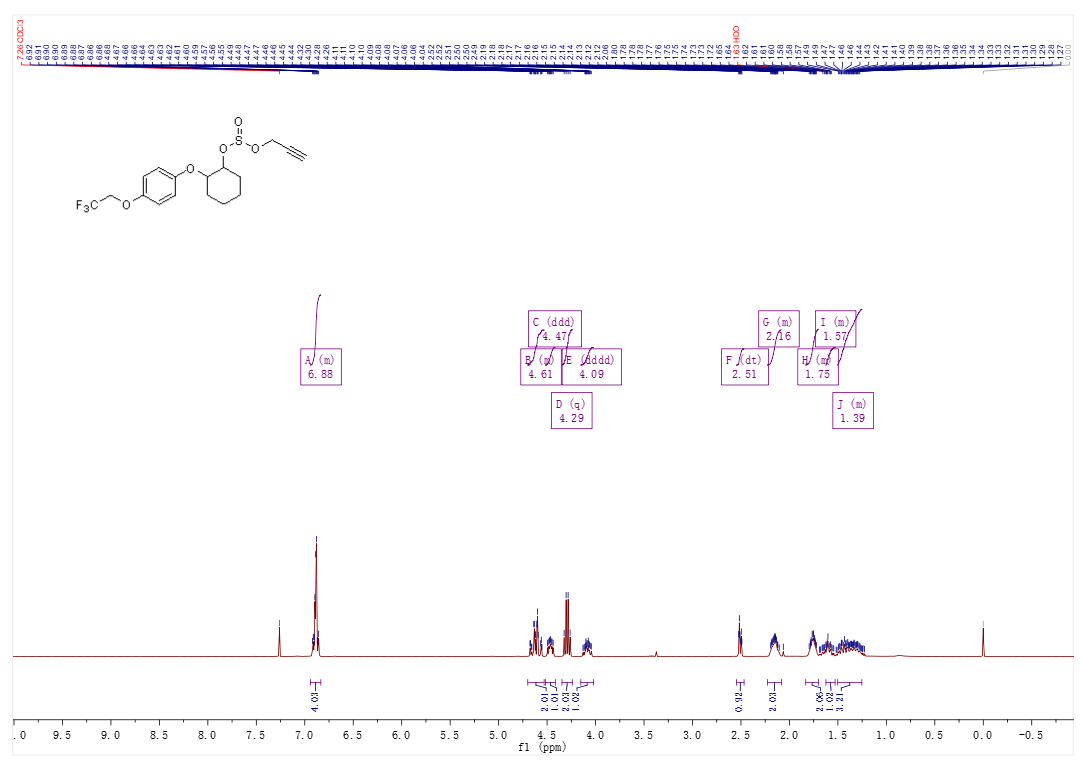


**Figure S40**. The ^1^H NMR spectrum of compound *prop-2-yn-1-yl (2-(4-(2,2,2-trifluoroethoxy)phenoxy)cyclohexyl) sulfite* (**5.14**)


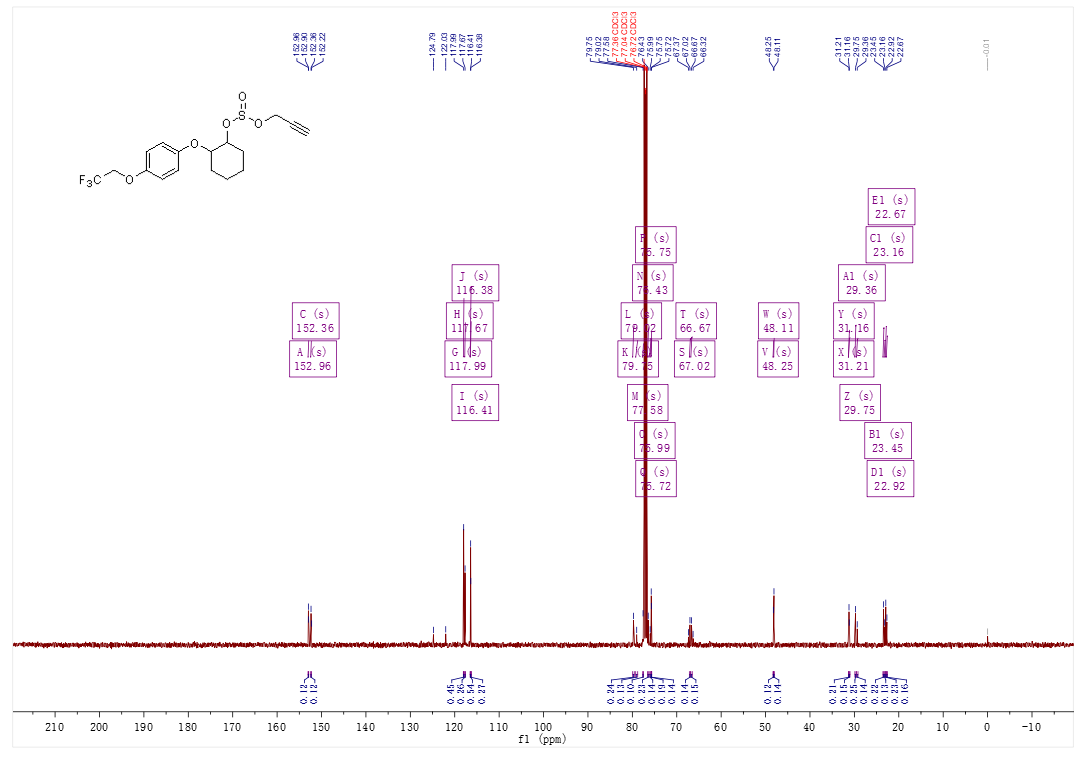


**Figure S41**. The ^13^C NMR spectrum of compound *prop-2-yn-1-yl (2-(4-(2,2,2-trifluoroethoxy)phenoxy)cyclohexyl) sulfite* (**5.14**)


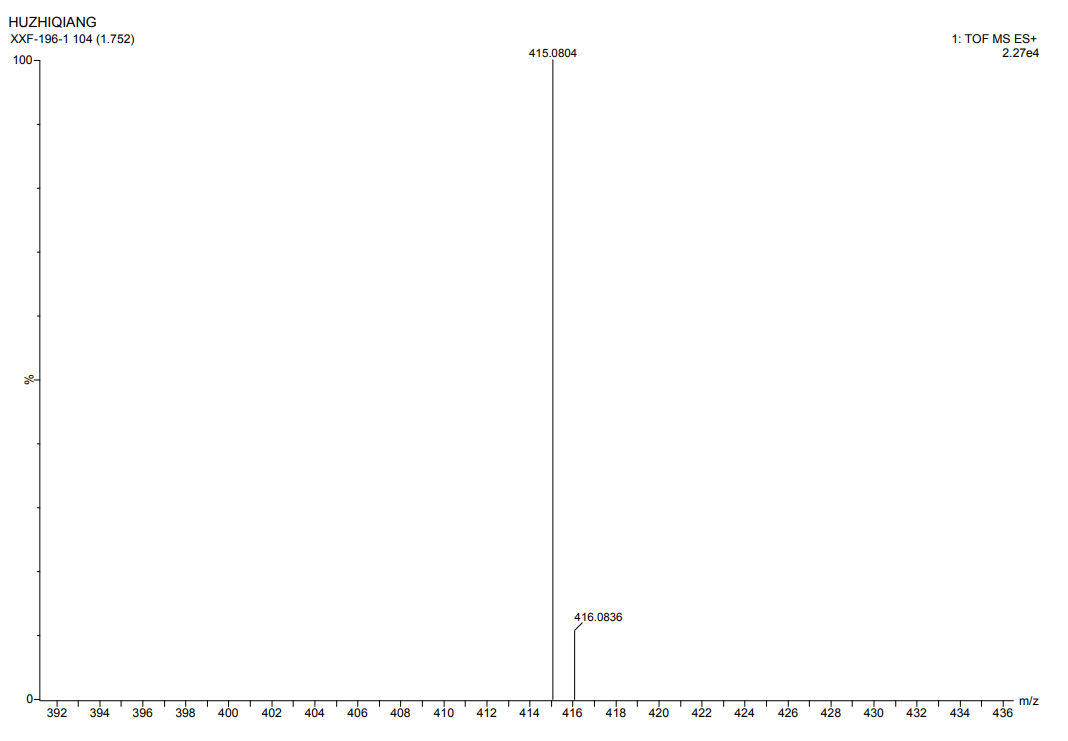


**Figure S42**. HRMS Spectrum of compound *prop-2-yn-1-yl (2-(4-(2,2,2-trifluoroethoxy)phenoxy)cyclohexyl) sulfite* (**5.14**)


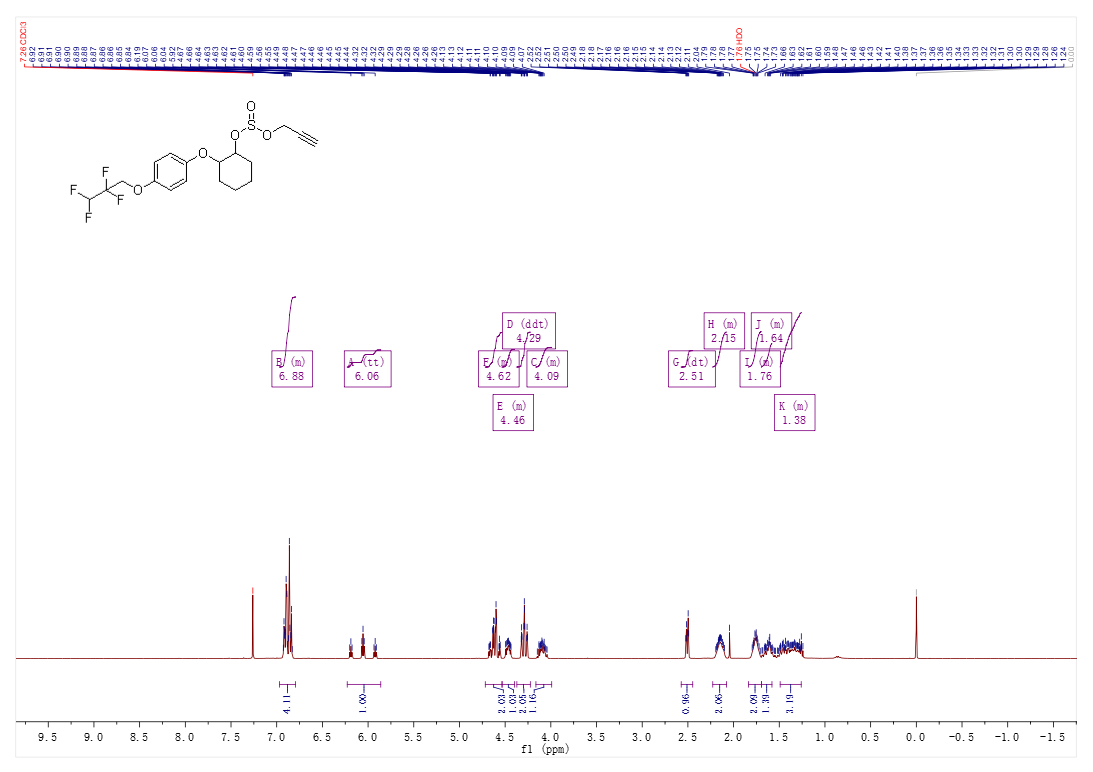


**Figure S43**. The ^1^H NMR spectrum of compound *prop-2-yn-1-yl (2-(4-(2,2,3,3-tetrafluoropropoxy)phenoxy)cyclohexyl) sulfite* (**5.15**)


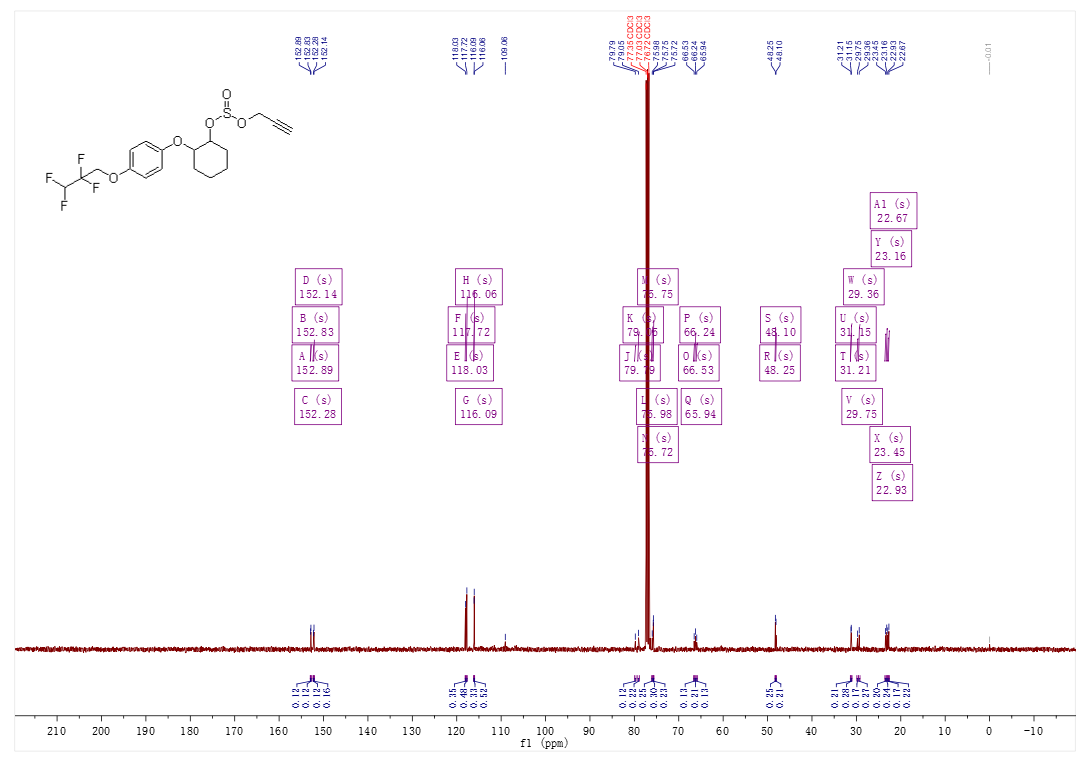


**Figure S44**. The ^13^C NMR spectrum of compound *prop-2-yn-1-yl (2-(4-(2,2,3,3-tetrafluoropropoxy)phenoxy)cyclohexyl) sulfite* (**5.15**)


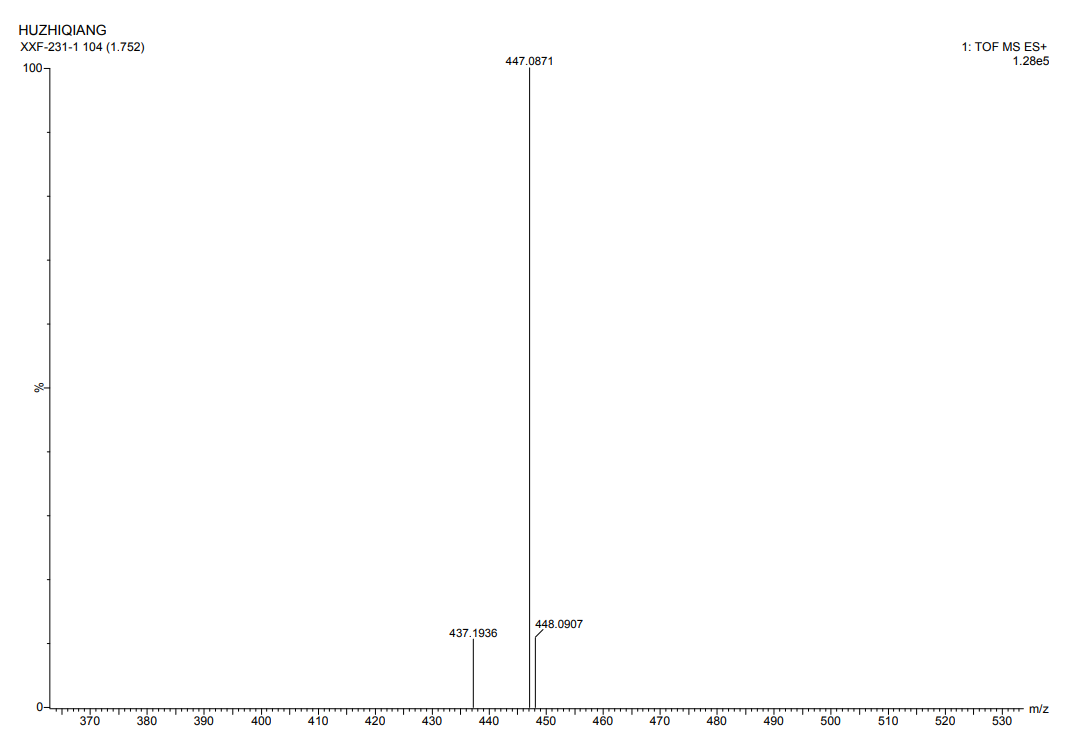


**Figure S45**. HRMS Spectrum of compound *prop-2-yn-1-yl (2-(4-(2,2,3,3-tetrafluoropropoxy)phenoxy)cyclohexyl) sulfite* (**5.15**)


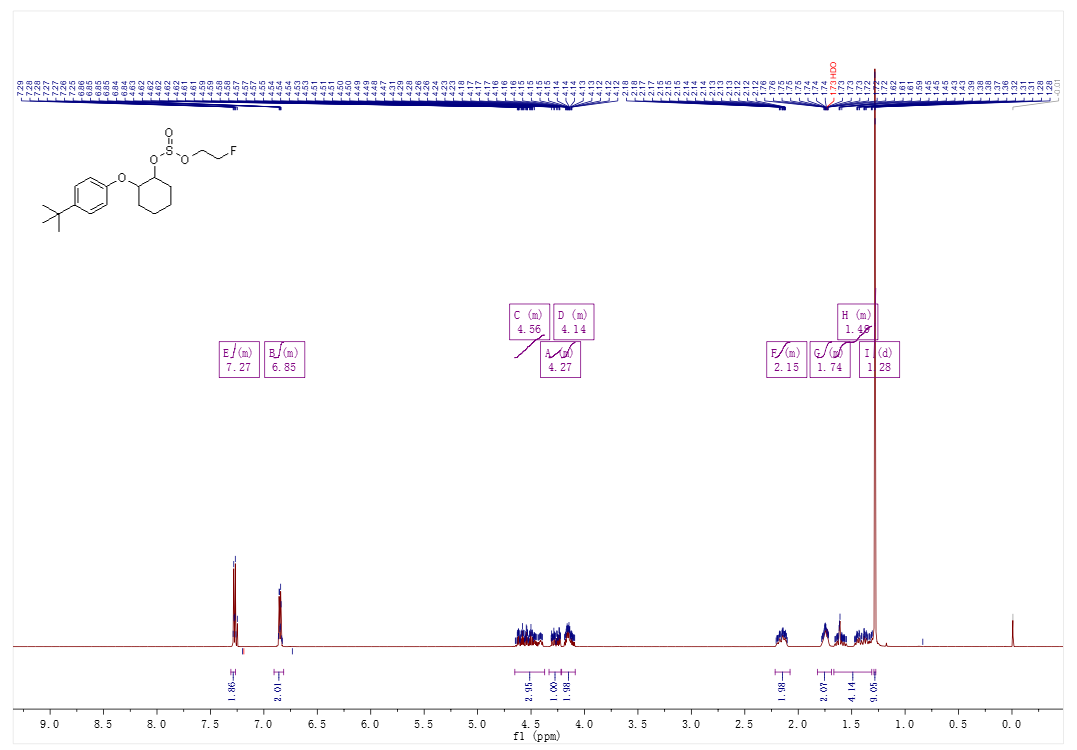


**Figure S46**. The ^1^H NMR spectrum of compound *2-(4-(tert-butyl)phenoxy)cyclohexyl (2-fluoroethyl) sulfite* (**5.16**)


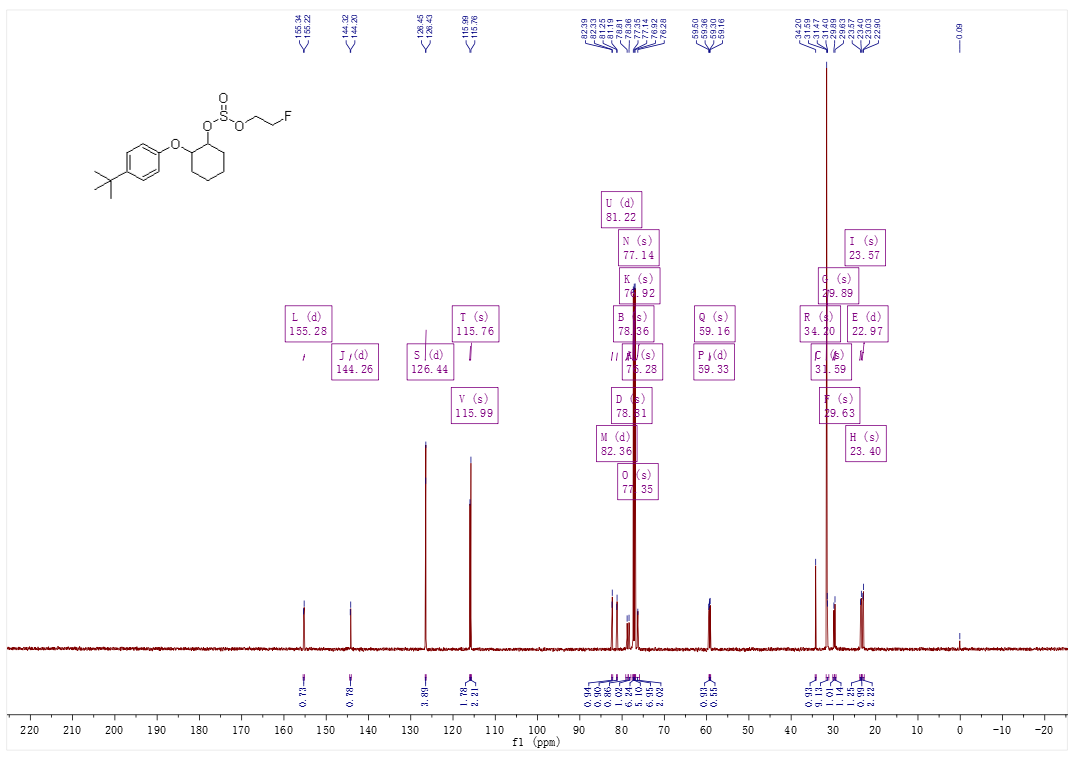


**Figure S47**. The ^13^C NMR spectrum of compound *2-(4-(tert-butyl)phenoxy)cyclohexyl (2-fluoroethyl) sulfite* (**5.16**)


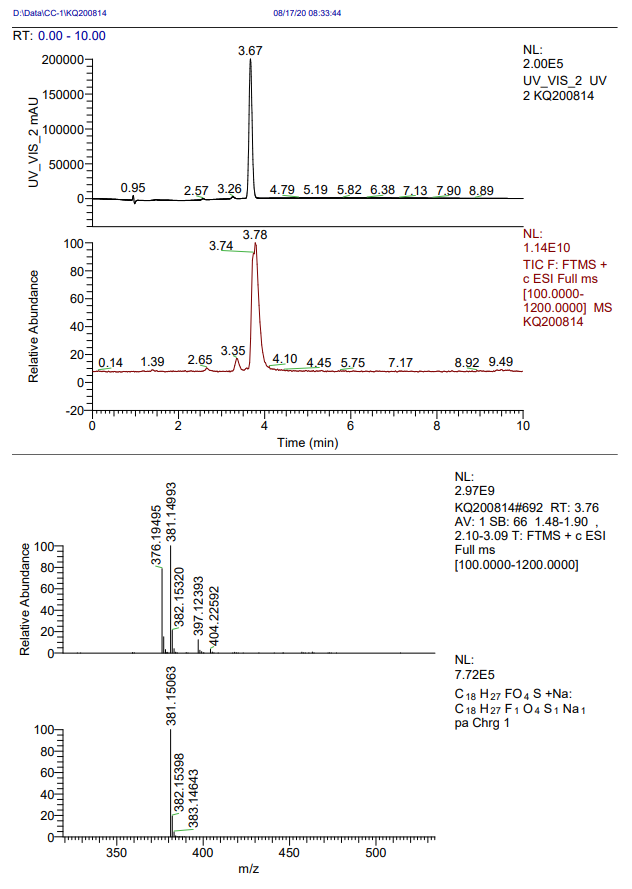


**Figure S48**. HRMS Spectrum of compound *2-(4-(tert-butyl)phenoxy)cyclohexyl (2-fluoroethyl) sulfite* (**5.16**)


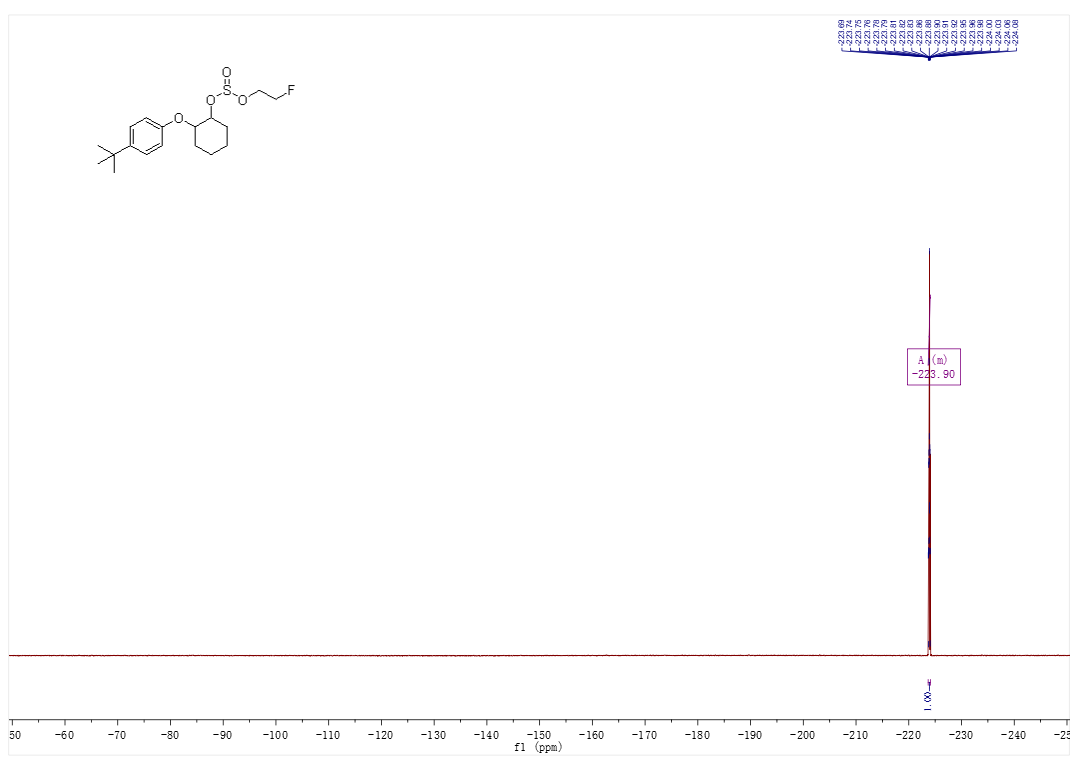


**Figure S49**. The F NMR spectrum of compound *2-(4-(tert-butyl)phenoxy)cyclohexyl (2-fluoroethyl) sulfite* (**5.16**)


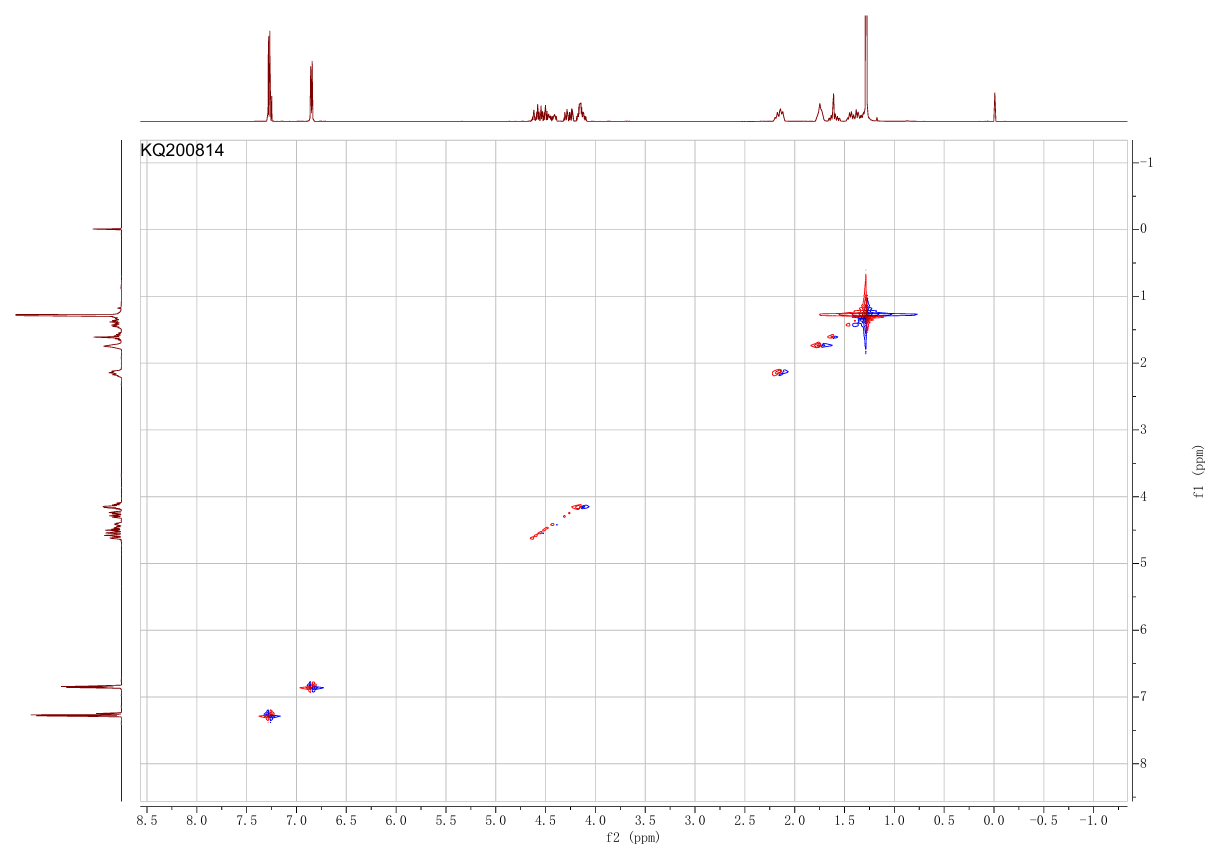


**Figure S50**. The NOESY spectrum of compound *2-(4-(tert-butyl)phenoxy)cyclohexyl (2-fluoroethyl) sulfite* (**5.16**)


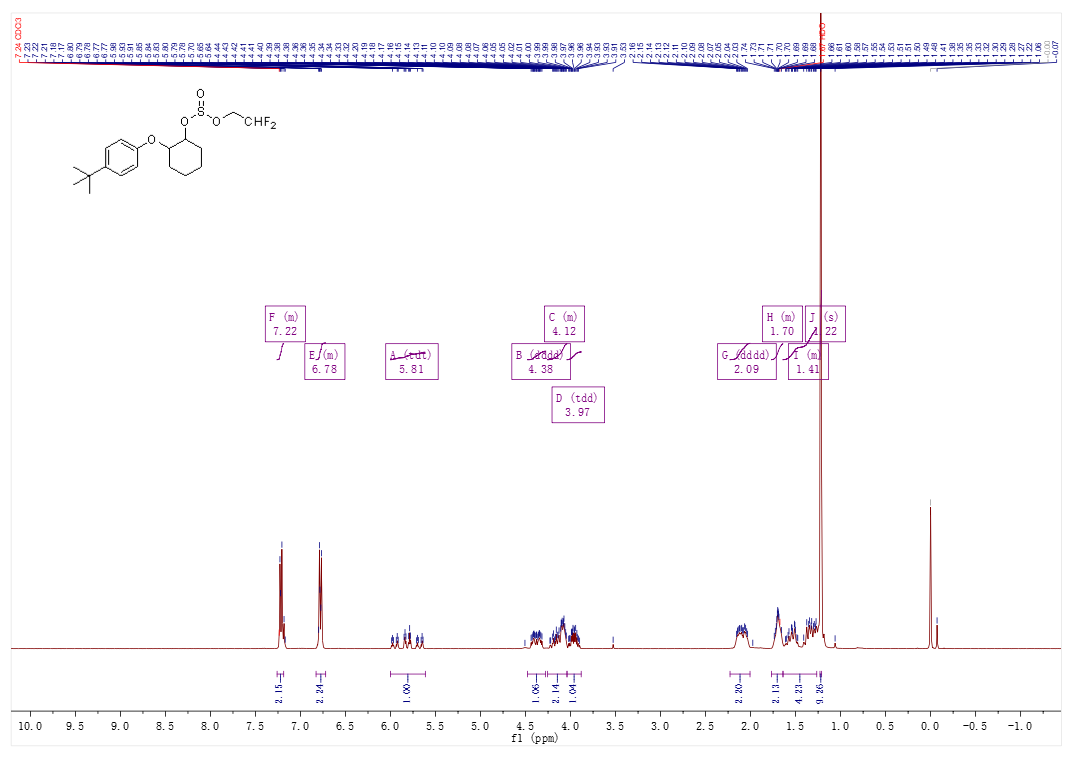


**Figure S51**. The ^1^H NMR spectrum of compound *2-(4-(tert-butyl)phenoxy)cyclohexyl (2,2-difluoroethyl) sulfite* (**5.17**)


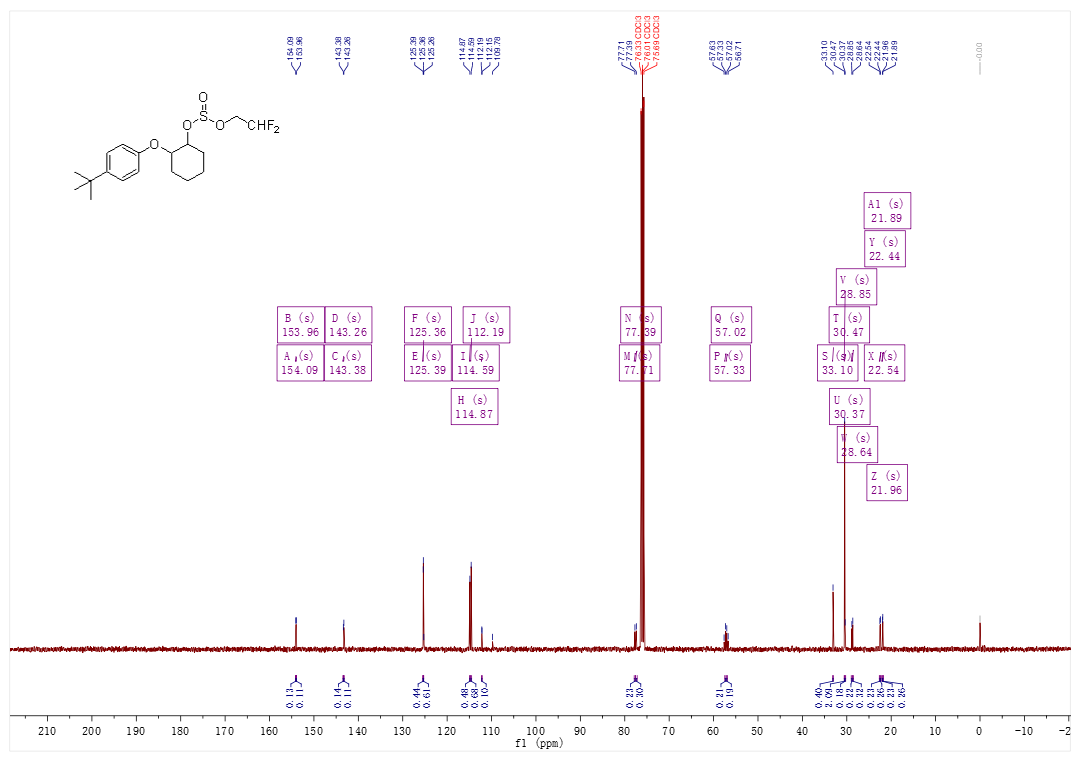


**Figure S52**. The ^13^C NMR spectrum of compound *2-(4-(tert-butyl)phenoxy)cyclohexyl (2,2-difluoroethyl) sulfite* (**5.17**)


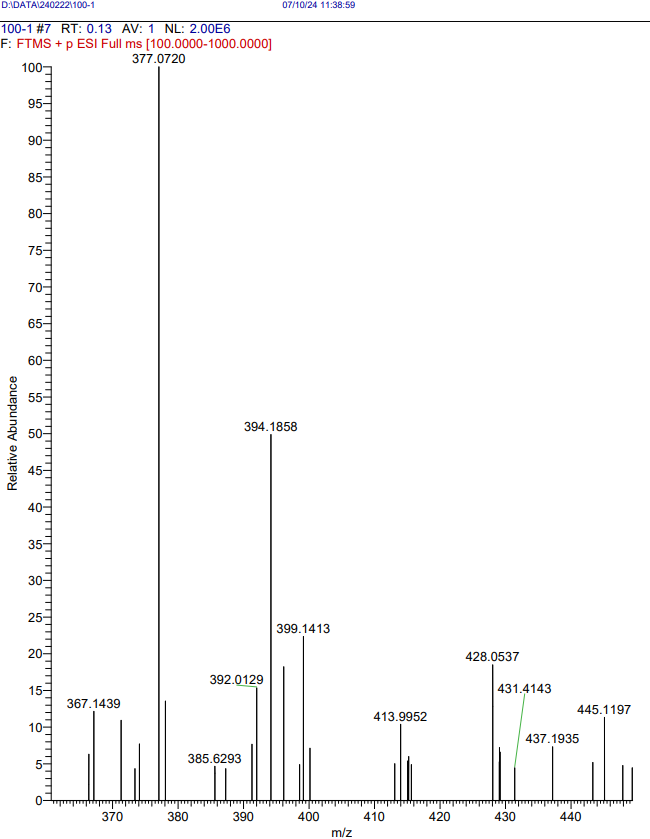


**Figure S53**. HRMS Spectrum of compound *2-(4-(tert-butyl)phenoxy)cyclohexyl (2,2-difluoroethyl) sulfite* (**5.17**)
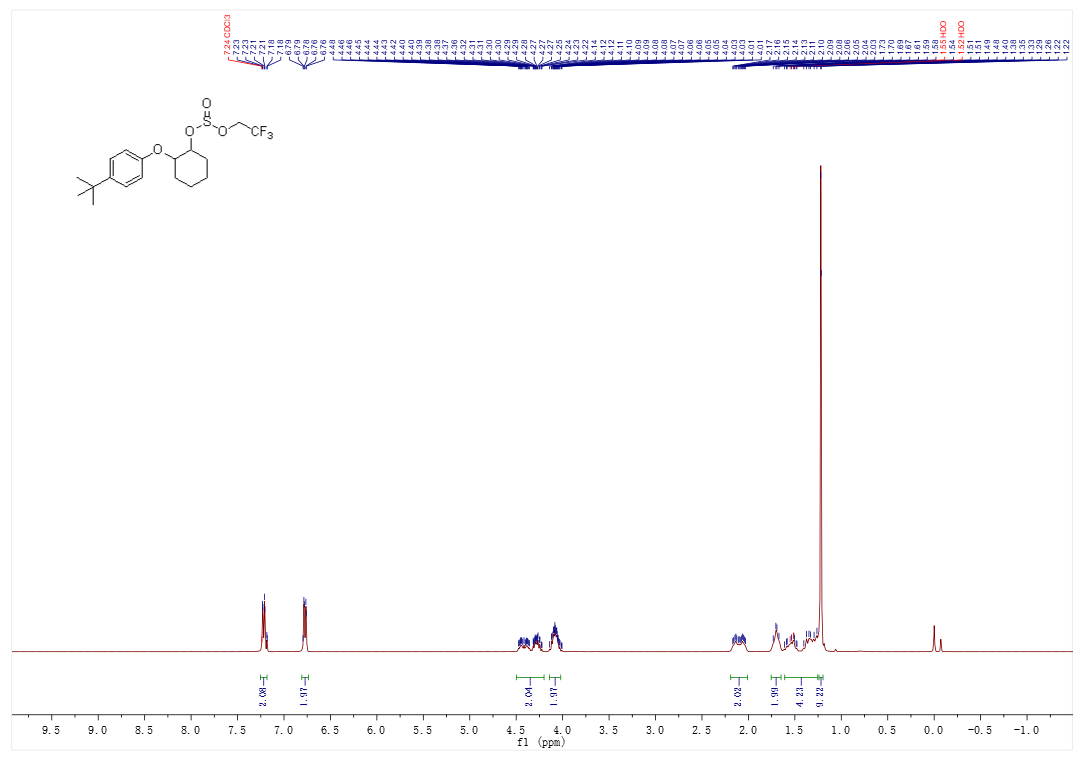


**Figure 54**. The ^1^H NMR spectrum of compound *2-(4-(tert-butyl)phenoxy)cyclohexyl (2,2,2-trifluoroethyl) sulfite* (**5.18**)


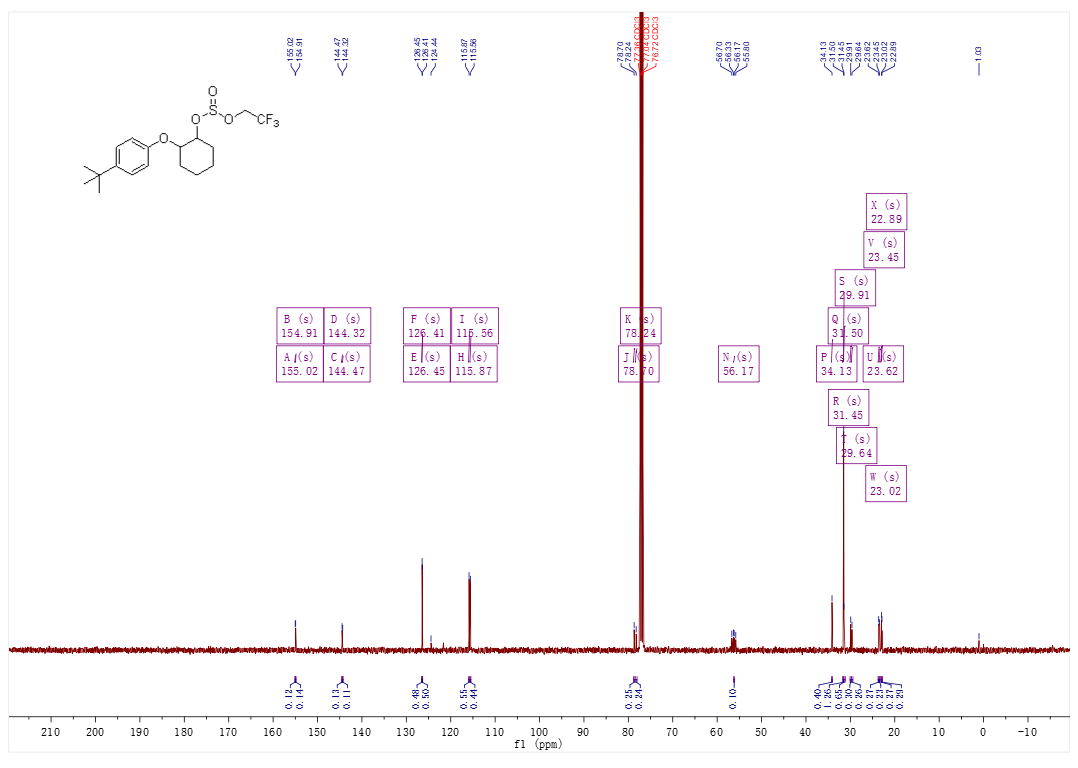


**Figure S55**. The ^13^C NMR spectrum of compound *2-(4-(tert-butyl)phenoxy)cyclohexyl (2,2,2-trifluoroethyl) sulfite* (**5.18**)


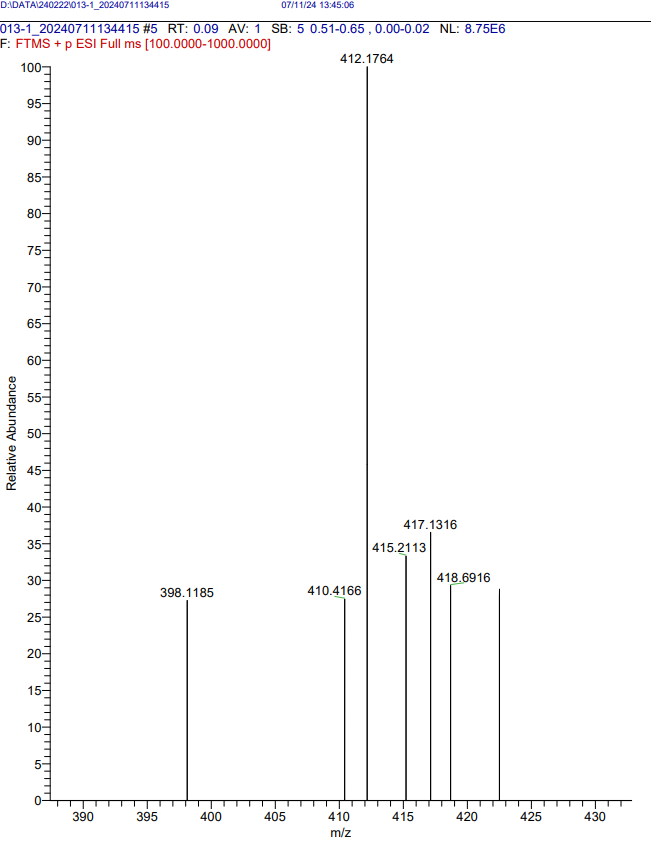


**Figure S56**. HRMS Spectrum of compound *2-(4-(tert-butyl)phenoxy)cyclohexyl (2,2,2-trifluoroethyl) sulfite* (**5.18**)
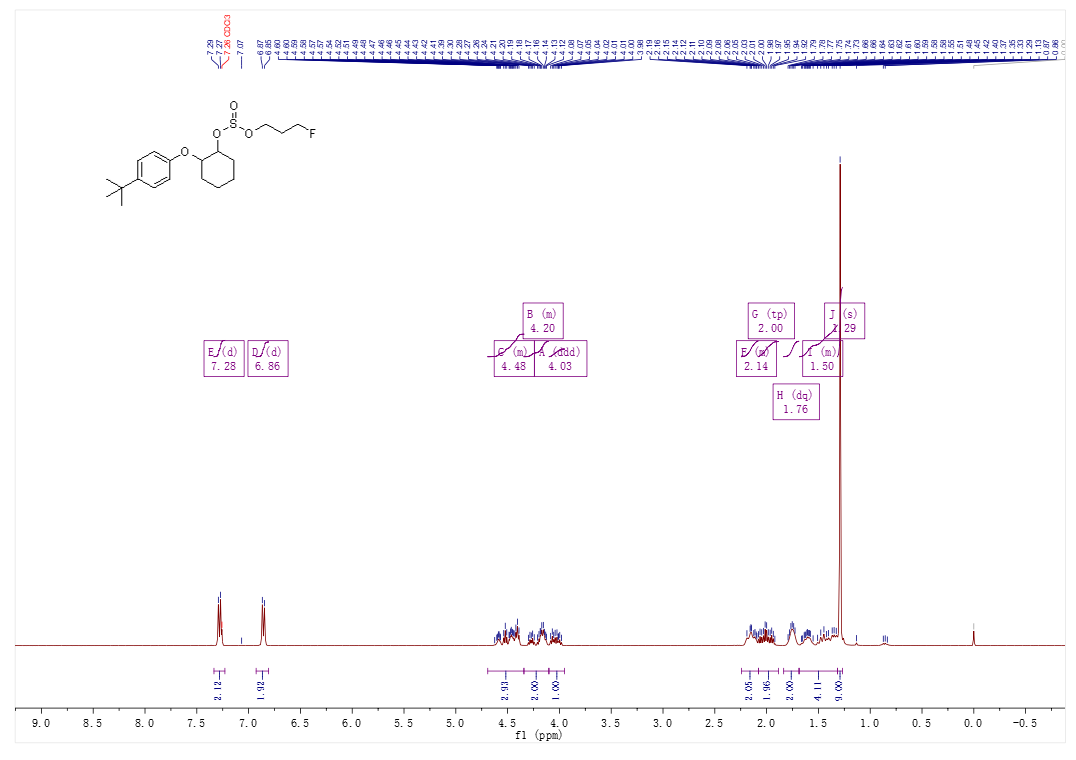


**Figure S57**. The ^1^H NMR spectrum of compound *2-(4-(tert-butyl)phenoxy)cyclohexyl (3-fluoropropyl) sulfite* (**5.19**)


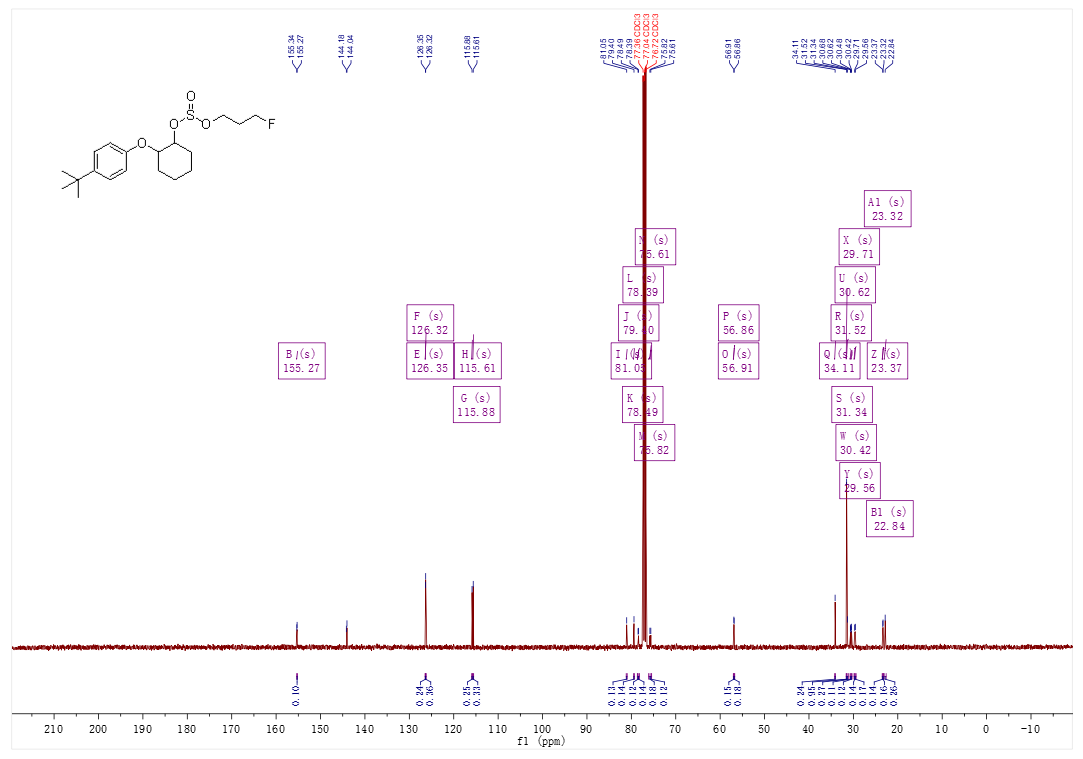


**Figure S58**. The ^13^C NMR spectrum of compound *2-(4-(tert-butyl)phenoxy)cyclohexyl (3-fluoropropyl) sulfite* (**5.19**)


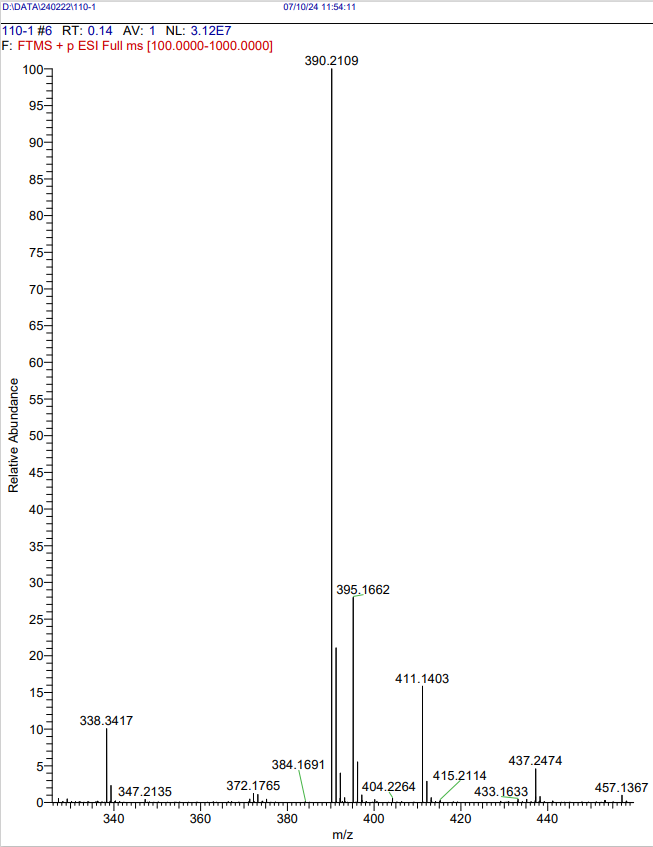


**Figure S59**. HRMS Spectrum of compound *2-(4-(tert-butyl)phenoxy)cyclohexyl (3-fluoropropyl) sulfite* (**5.19**)
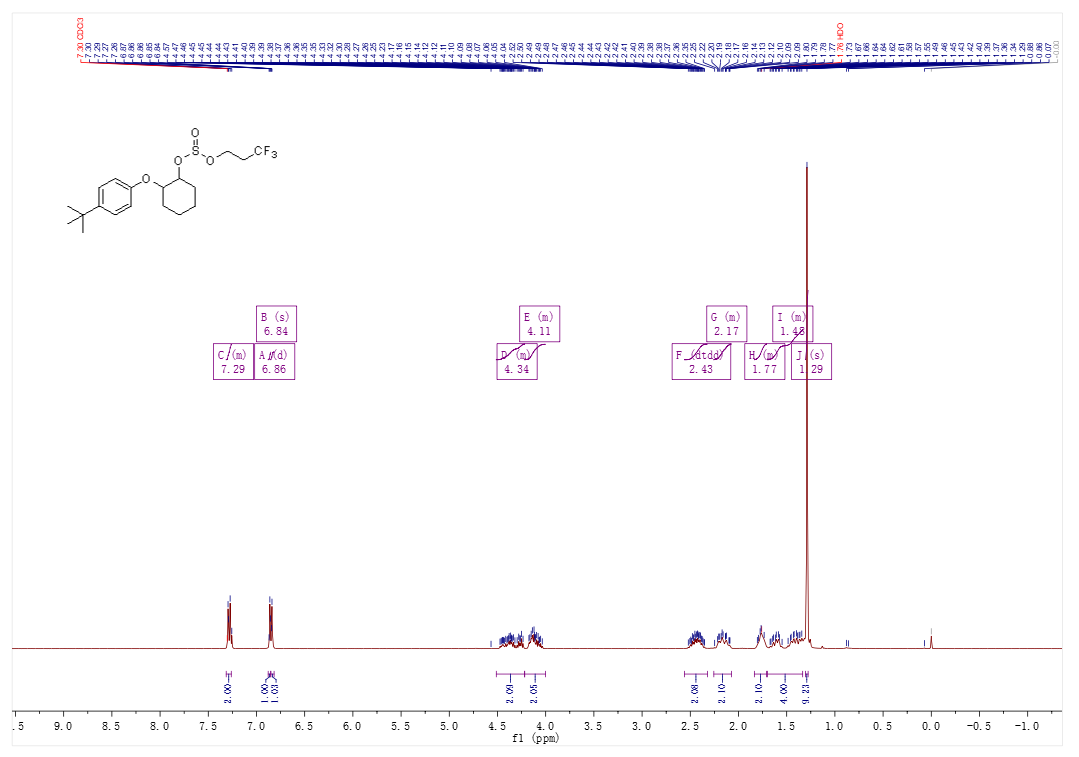


**Figure S60**. The ^1^H NMR spectrum of compound *2-(4-(tert-butyl)phenoxy)cyclohexyl (3,3,3-trifluoropropyl) sulfite* (**5.20**)


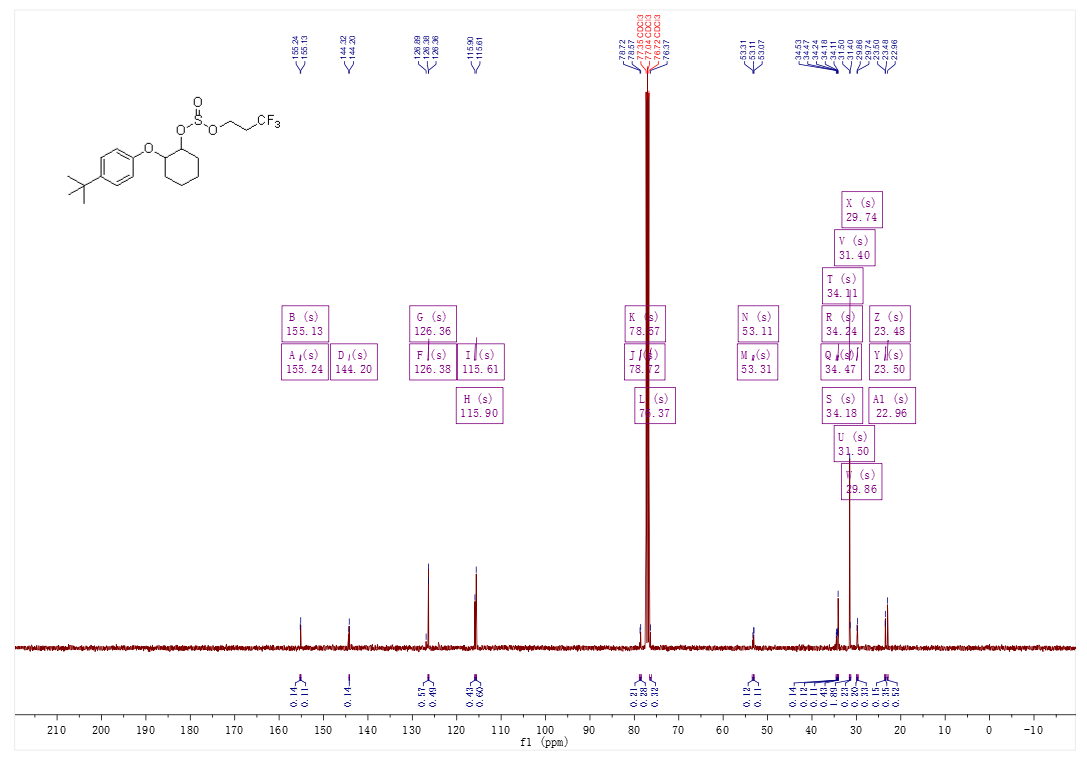


**Figure S61**. The ^13^C NMR spectrum of compound *2-(4-(tert-butyl)phenoxy)cyclohexyl (3,3,3-trifluoropropyl) sulfite* (**5.20**)
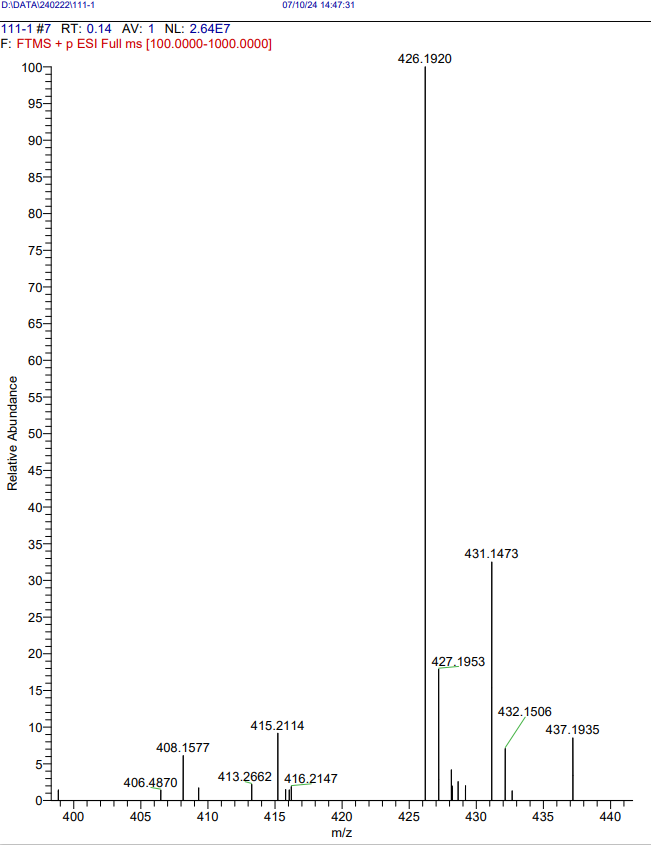


**Figure S62**. HRMS Spectrum of compound *2-(4-(tert-butyl)phenoxy)cyclohexyl (3,3,3-trifluoropropyl) sulfite* (**5.20**)
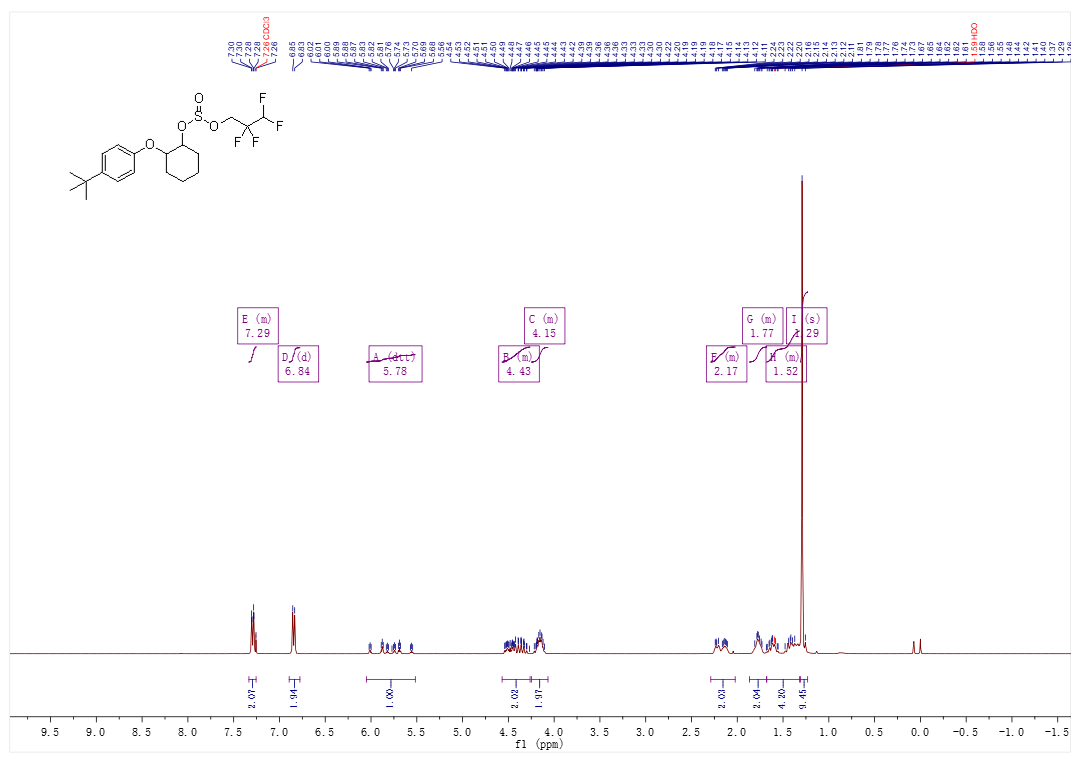


**Figure S63**. The ^1^H NMR spectrum of compound *2-(4-(tert-butyl)phenoxy)cyclohexyl (2,2,3,3-tetrafluoropropyl) sulfite* (**5.21**)


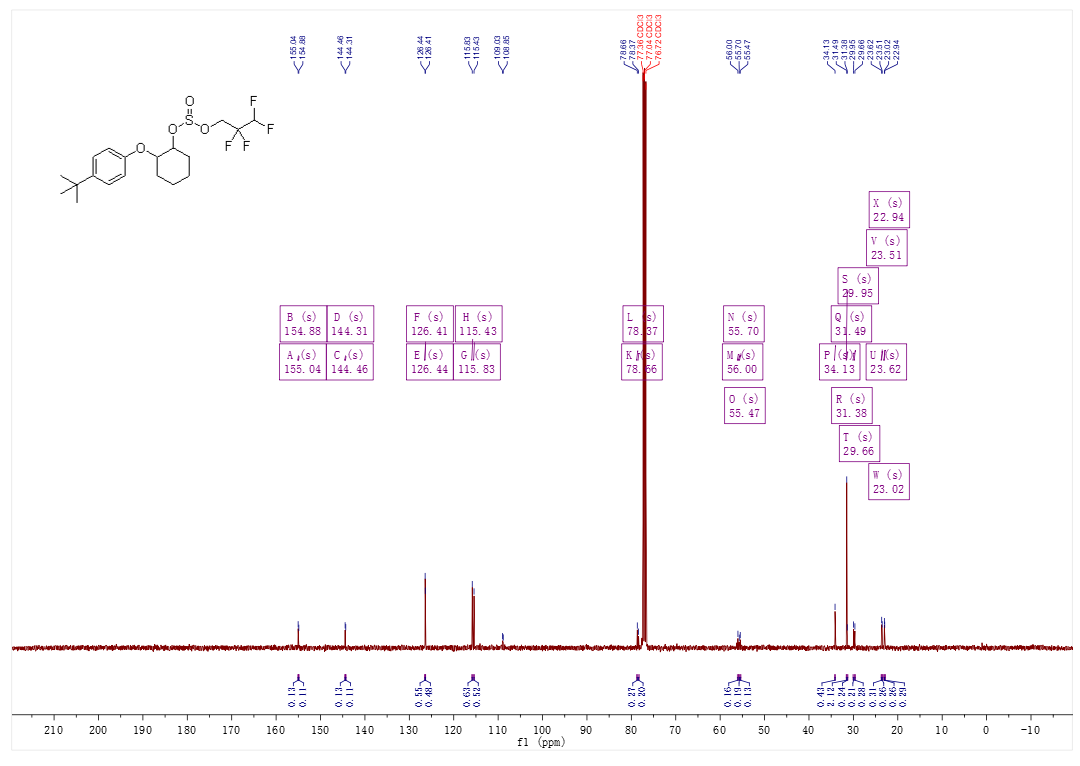


**Figure S64**. The ^13^C NMR spectrum of compound *2-(4-(tert-butyl)phenoxy)cyclohexyl (2,2,3,3-tetrafluoropropyl) sulfite* (**5.21**)


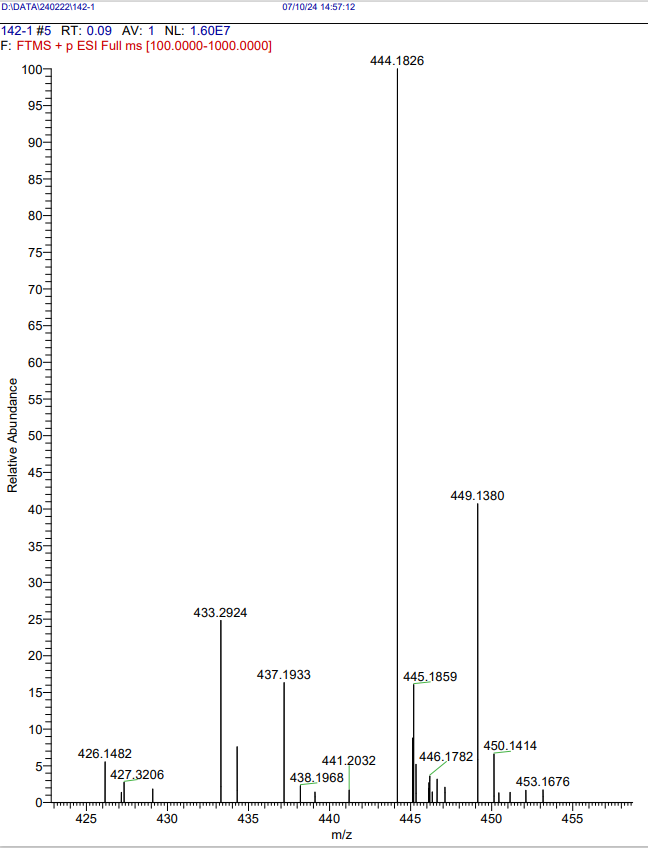


**Figure S65**. HRMS Spectrum of compound *2-(4-(tert-butyl)phenoxy)cyclohexyl (2,2,3,3-tetrafluoropropyl) sulfite* (**5.21**)


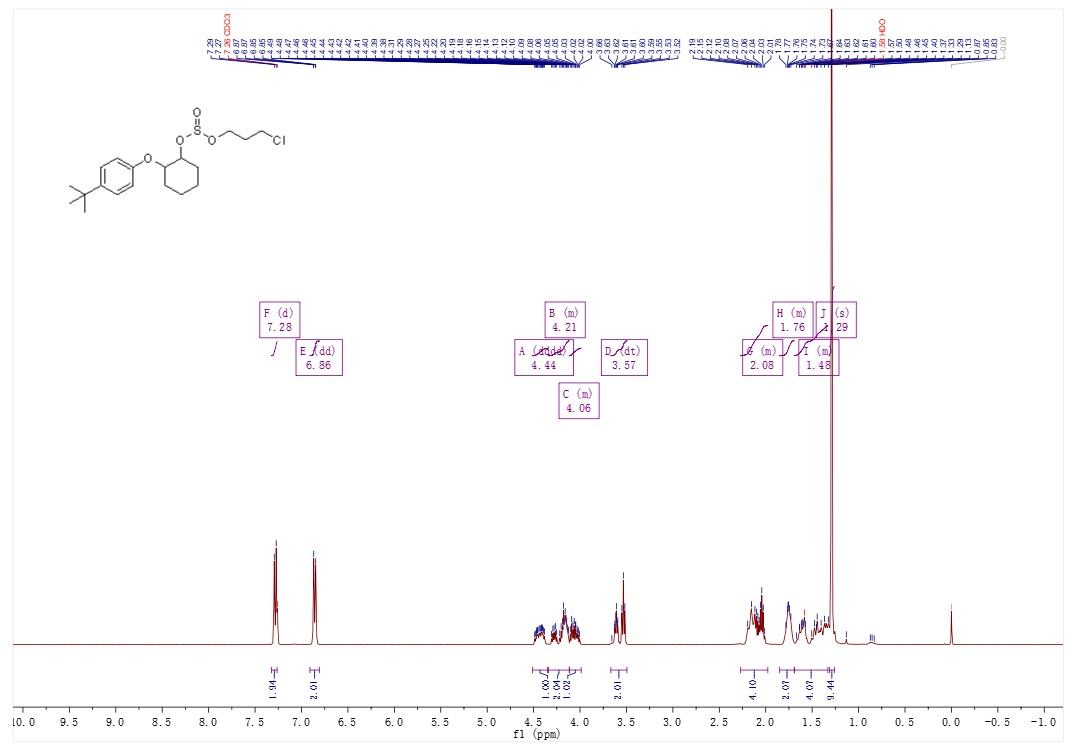


**Figure S66**. The ^1^H NMR spectrum of compound *2-(4-(tert-butyl)phenoxy)cyclohexyl (3-chloropropyl) sulfite* (**5.22**)


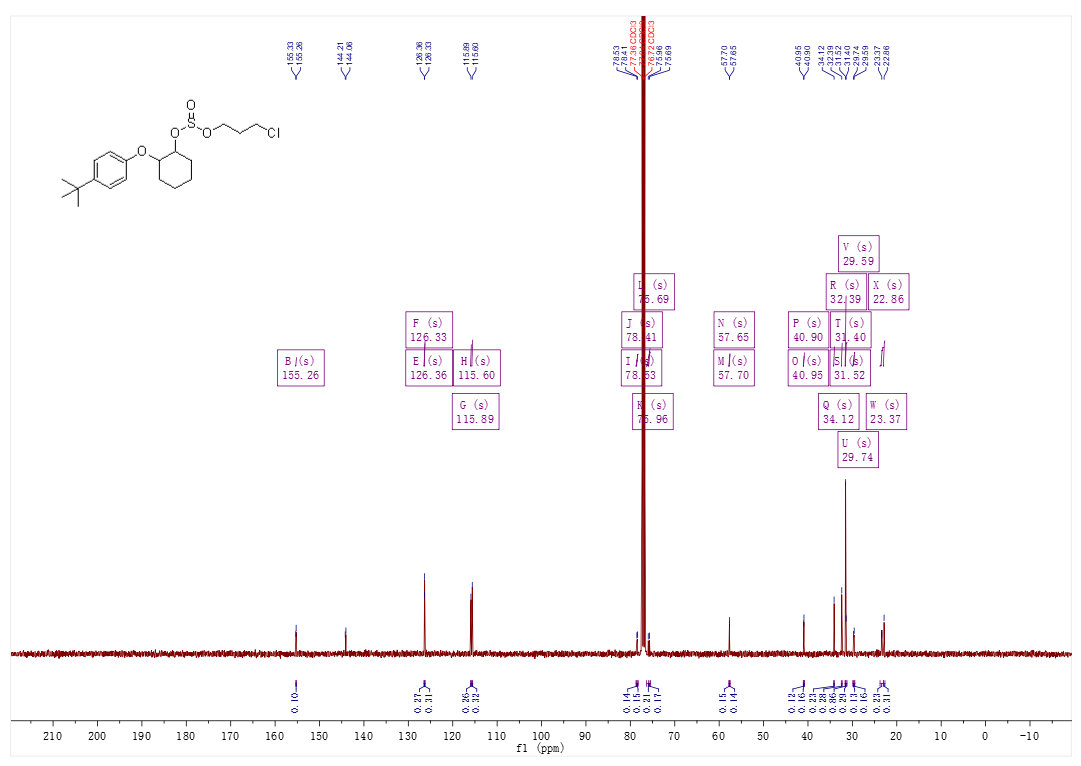


**Figure S67**. The ^13^C NMR spectrum of compound *2-(4-(tert-butyl)phenoxy)cyclohexyl (3-chloropropyl) sulfite* (**5.22**)


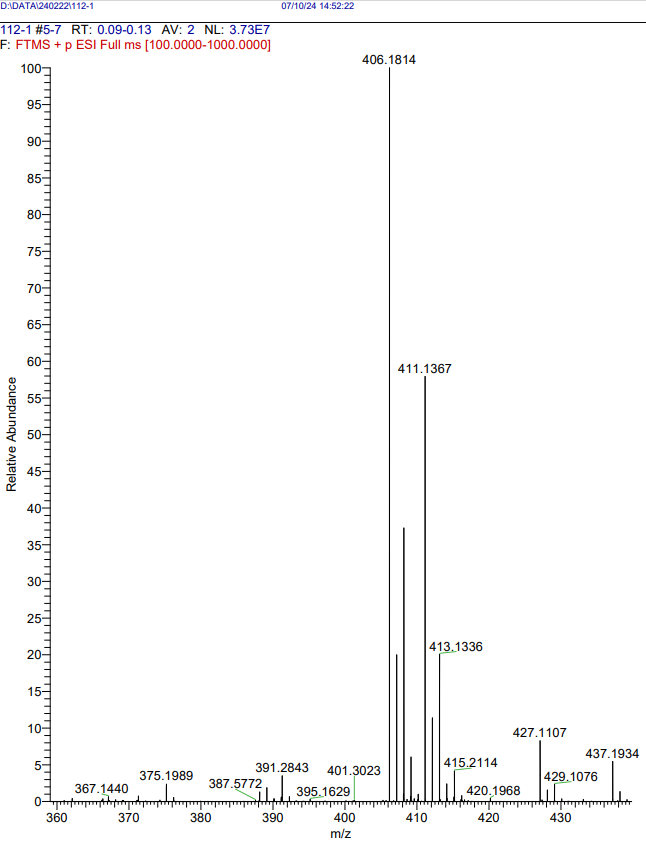


**Figure S68**. HRMS Spectrum of compound *2-(4-(tert-butyl)phenoxy)cyclohexyl (3-chloropropyl) sulfite* (**5.22**)
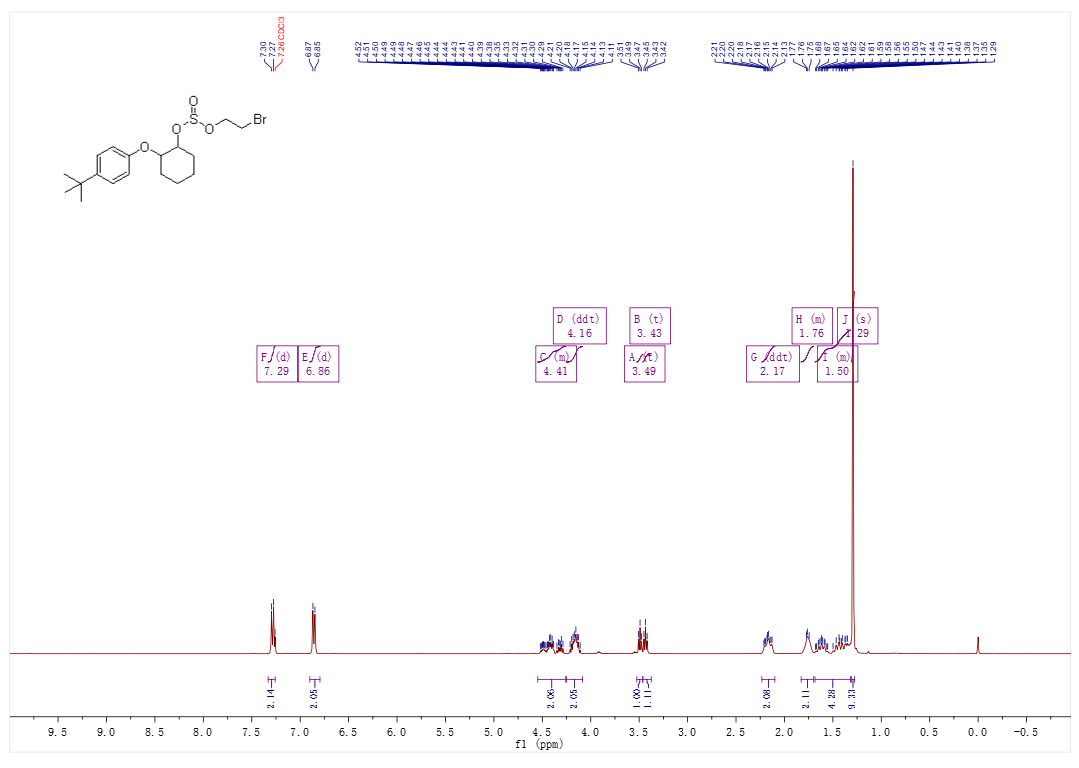


**Figure S69**. The ^1^H NMR spectrum of compound *2-bromoethyl (2-(4-(tert-butyl)phenoxy)cyclohexyl) sulfite* (**5.23**)


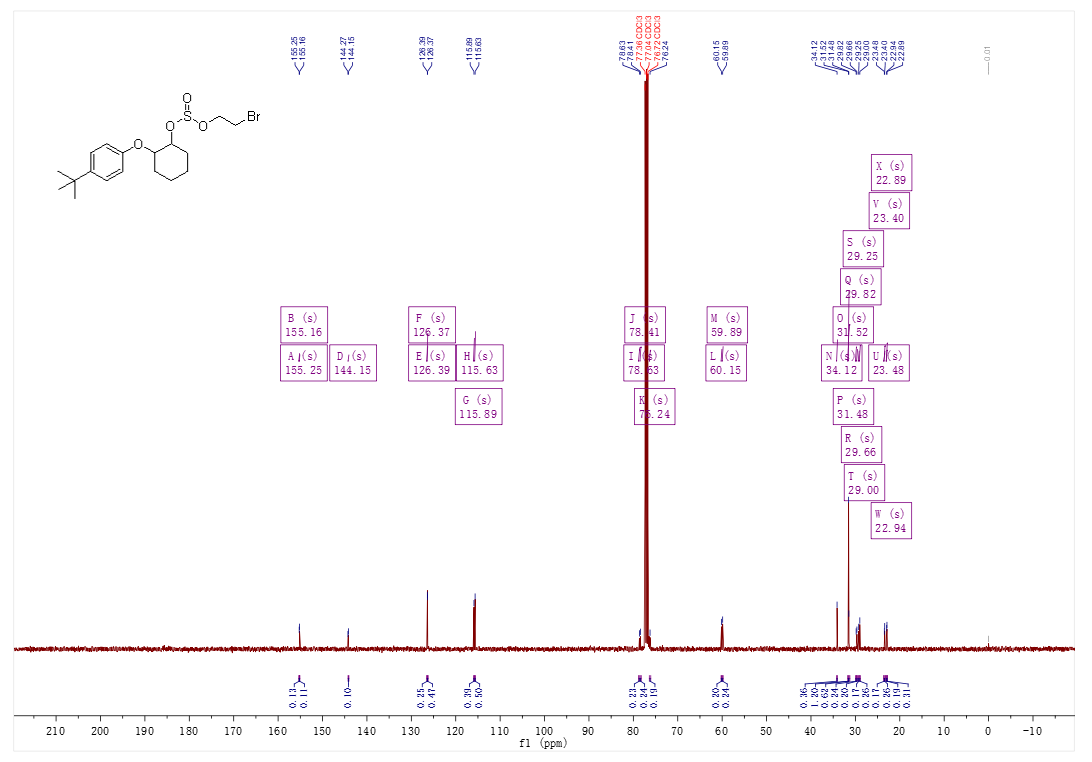


**Figure S70**. The ^13^C NMR spectrum of compound *2-bromoethyl (2-(4-(tert-butyl)phenoxy)cyclohexyl) sulfite* (**5.23**)


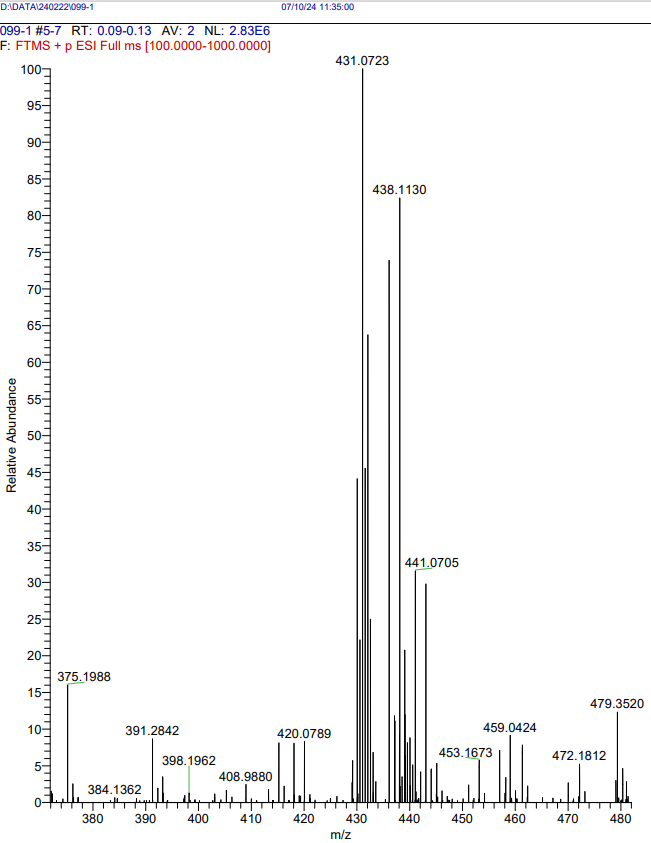


**Figure S71**. HRMS Spectrum of compound *2-bromoethyl (2-(4-(tert-butyl)phenoxy)cyclohexyl) sulfite* (**5.23**)
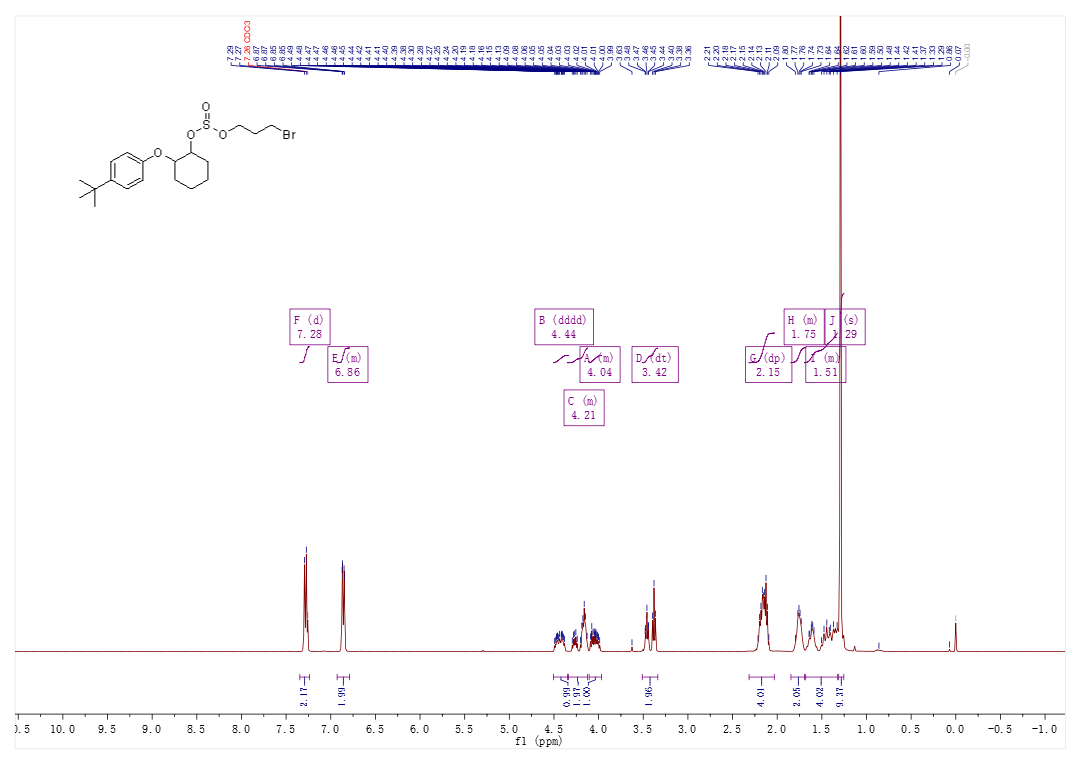


**Figure S72**. The ^1^H NMR spectrum of compound *3-bromopropyl (2-(4-(tert-butyl)phenoxy)cyclohexyl) sulfite* (**5.24**)


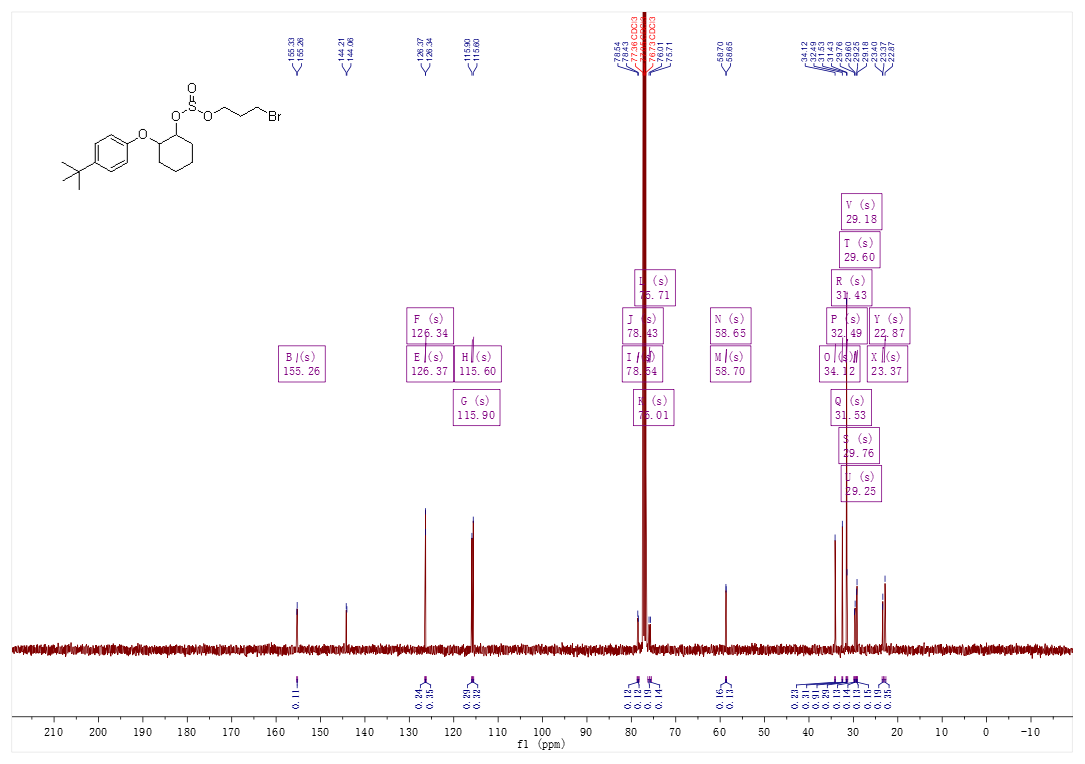


**Figure S73**. The ^13^C NMR spectrum of compound *3-bromopropyl (2-(4-(tert-butyl)phenoxy)cyclohexyl) sulfite* (**5.24**)


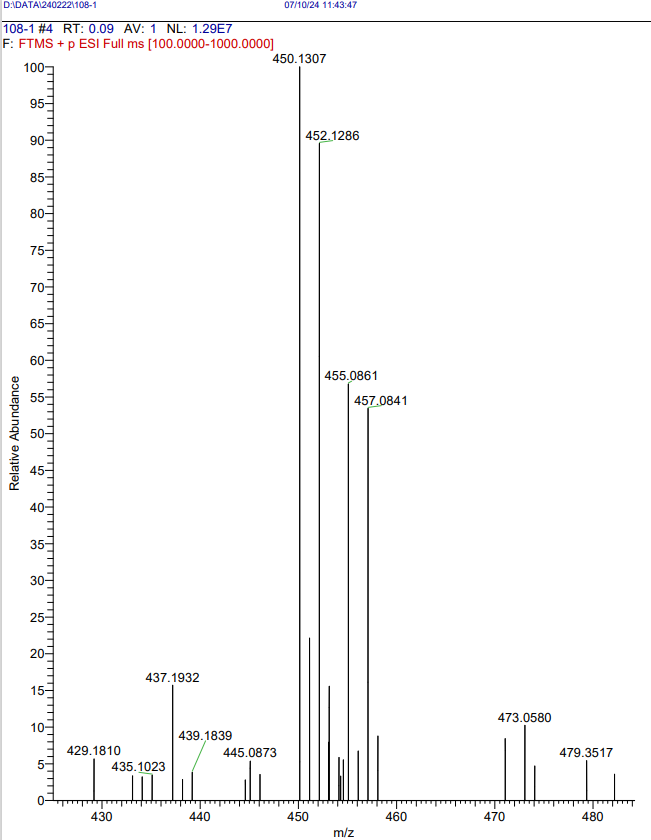


**Figure S74**. HRMS Spectrum of compound *3-bromopropyl (2-(4-(tert-butyl)phenoxy)cyclohexyl) sulfite* (**5.24**)
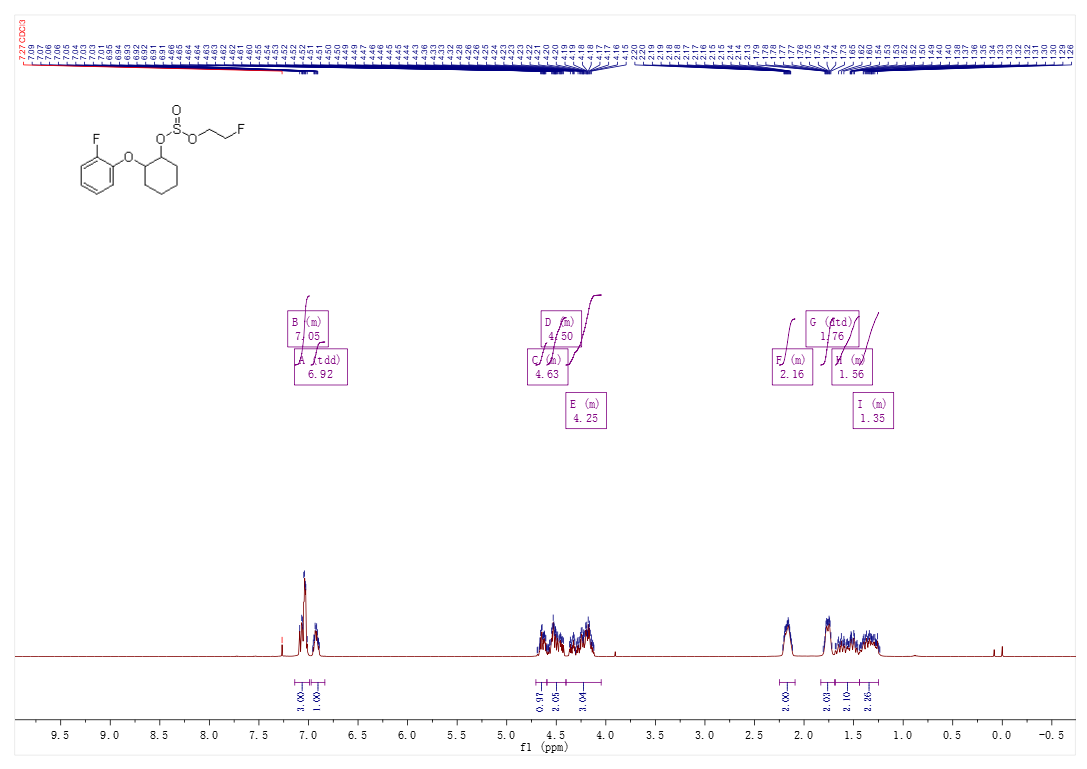


**Figure S75**. The ^1^H NMR spectrum of compound *2-fluoroethyl (2-(2-fluorophenoxy)cyclohexyl) sulfite* (**5.25**)


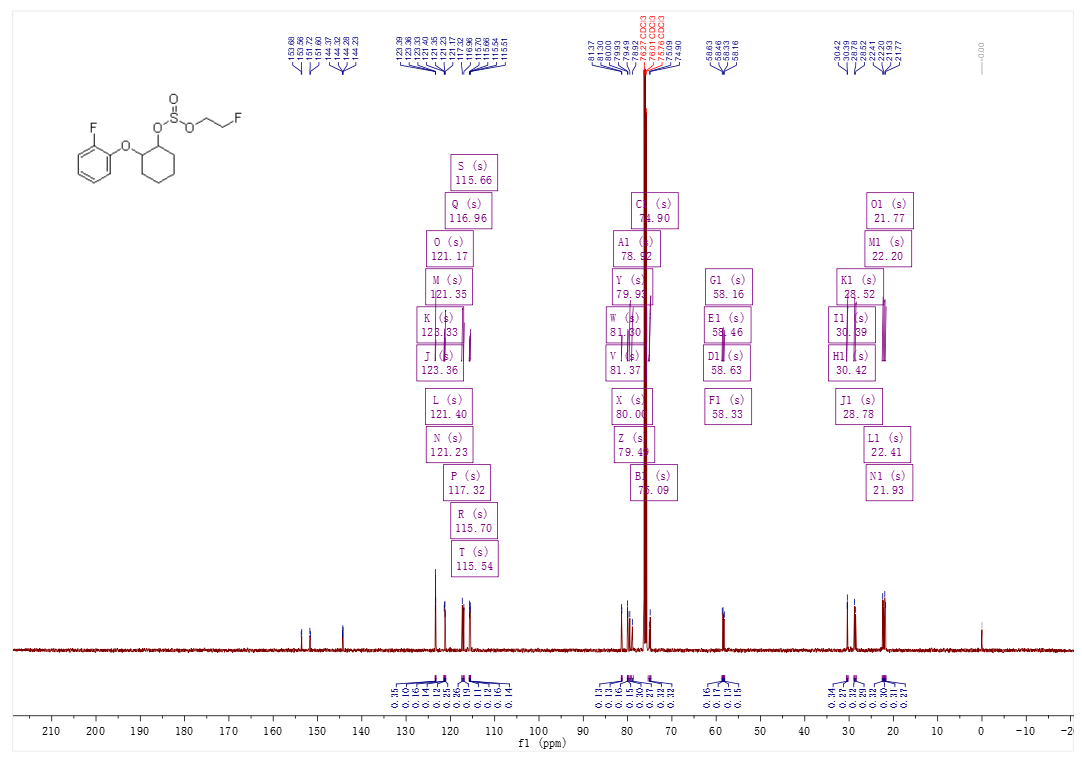


**Figure S76**. The ^13^C NMR spectrum of compound *2-fluoroethyl (2-(2-fluorophenoxy)cyclohexyl) sulfite* (**5.25**)


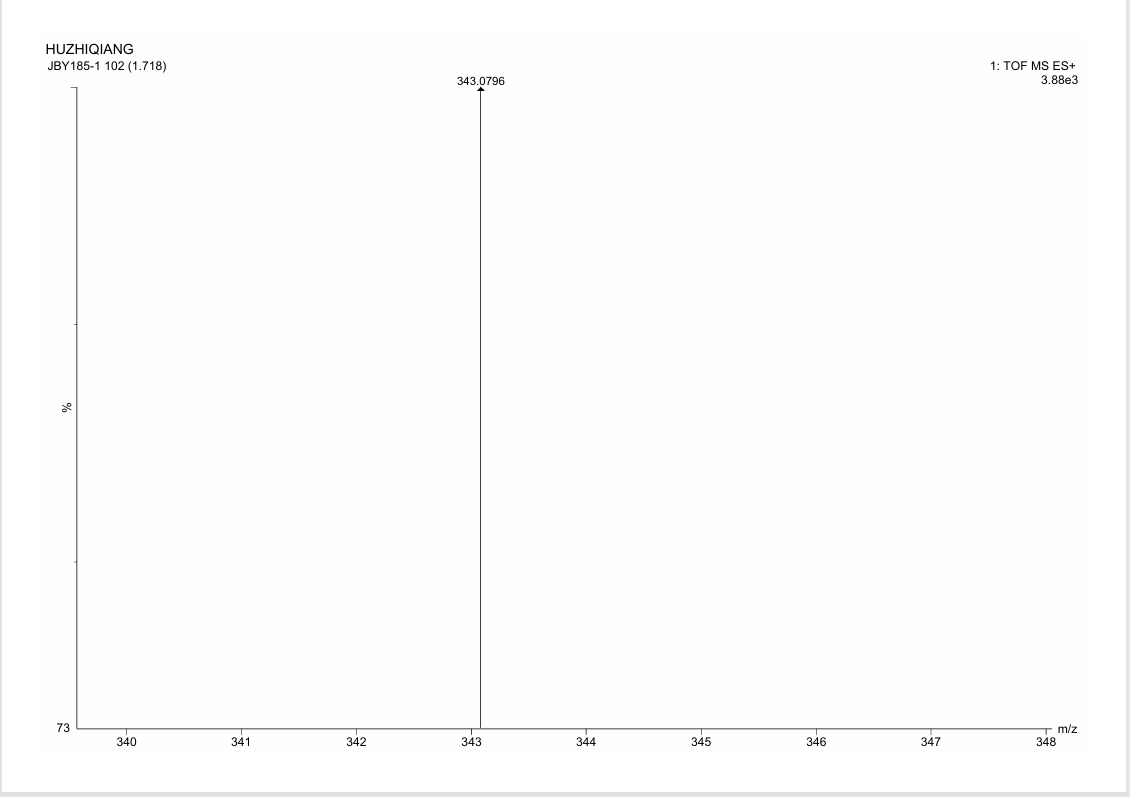


**Figure S77**. HRMS Spectrum of compound *2-fluoroethyl (2-(2-fluorophenoxy)cyclohexyl) sulfite* (**5.25**)


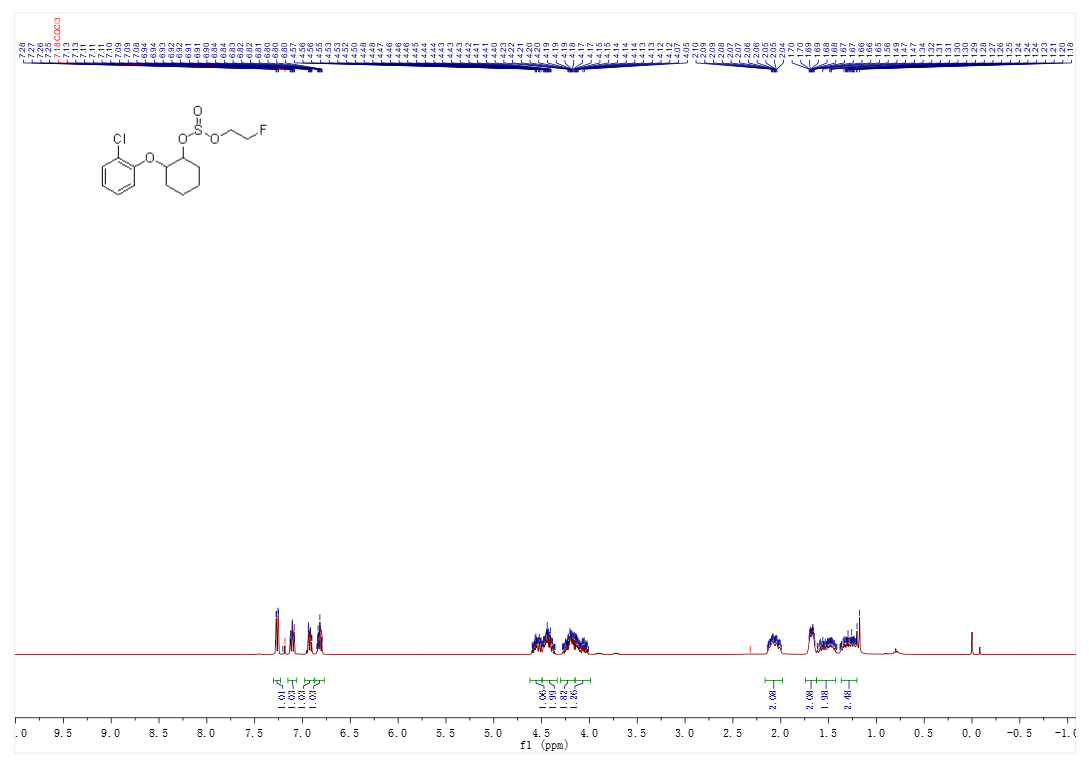


**Figure S78**. The ^1^H NMR spectrum of compound *2-(2-chlorophenoxy)cyclohexyl (2-fluoroethyl) sulfite* (**5.26**)


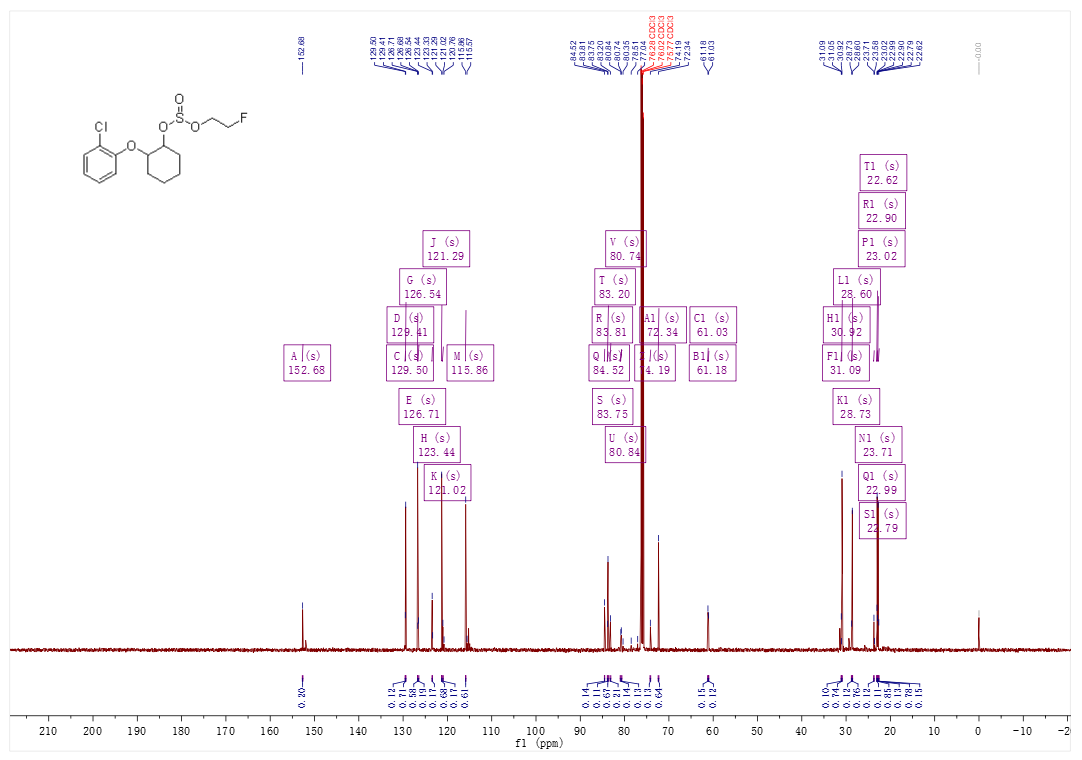


**Figure S79**. The ^13^C NMR spectrum of compound *2-(2-chlorophenoxy)cyclohexyl (2-fluoroethyl) sulfite* (**5.26**)


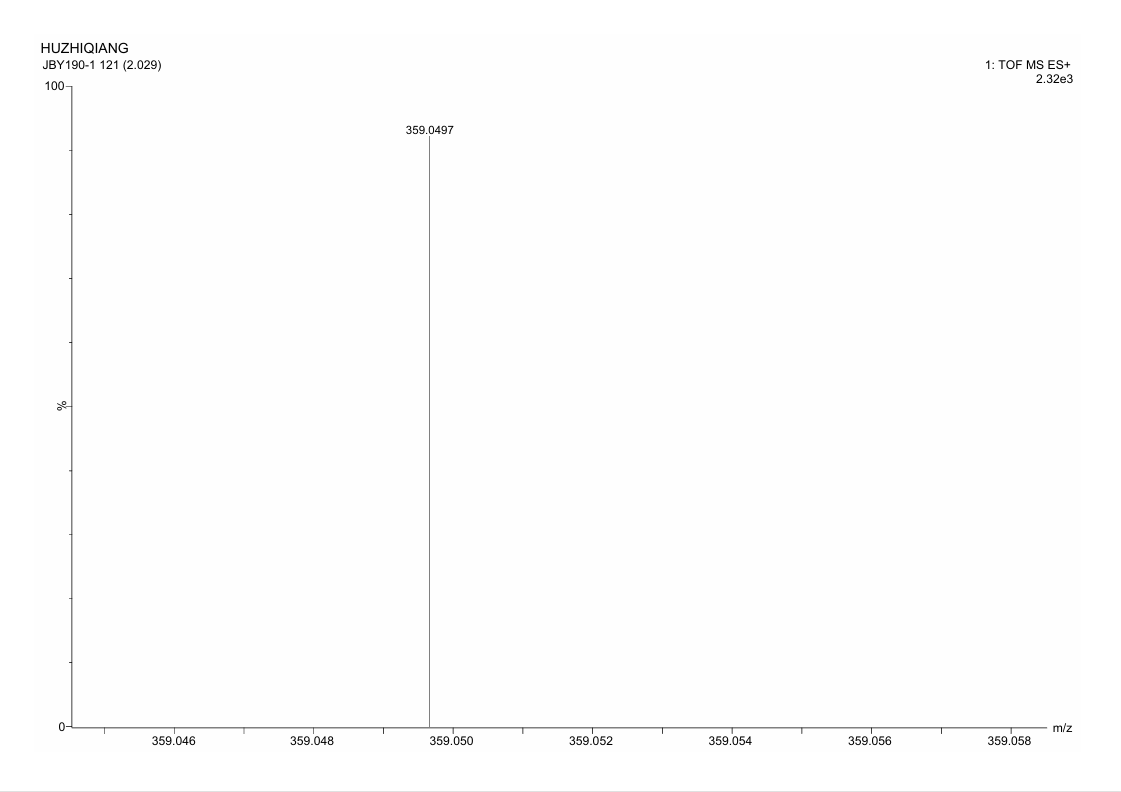


**Figure S80**. HRMS Spectrum of compound *2-(2-chlorophenoxy)cyclohexyl (2-fluoroethyl) sulfite* (**5.26**)


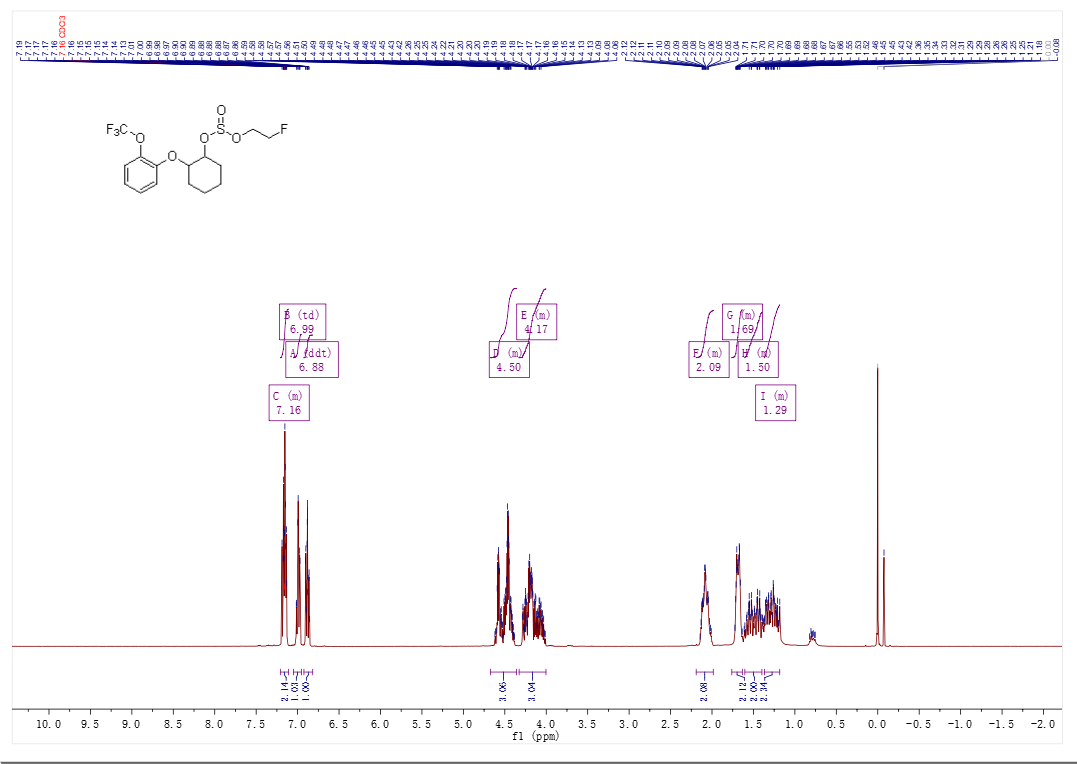


**Figure S81**. The ^1^H NMR spectrum of compound *2-fluoroethyl (2-(2-(trifluoromethoxy)phenoxy)cyclohexyl) sulfite* (**5.27**)


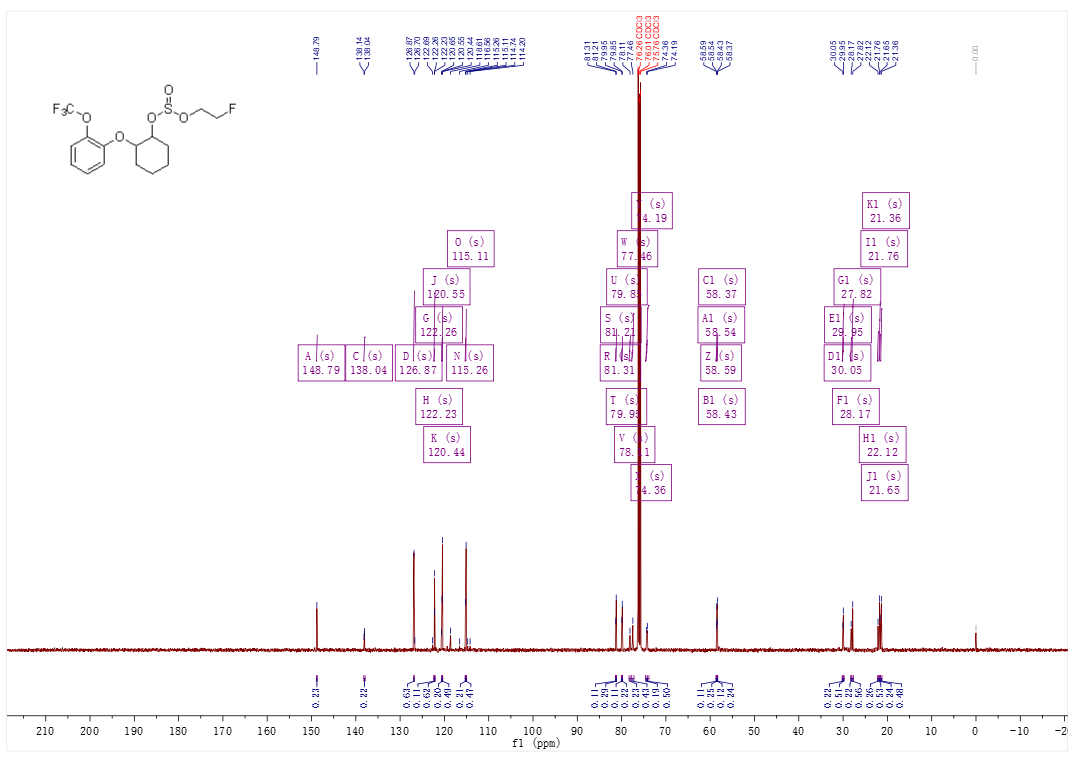


**Figure S82**. The ^13^C NMR spectrum of compound *2-fluoroethyl (2-(2-(trifluoromethoxy)phenoxy)cyclohexyl) sulfite* (**5.27**)


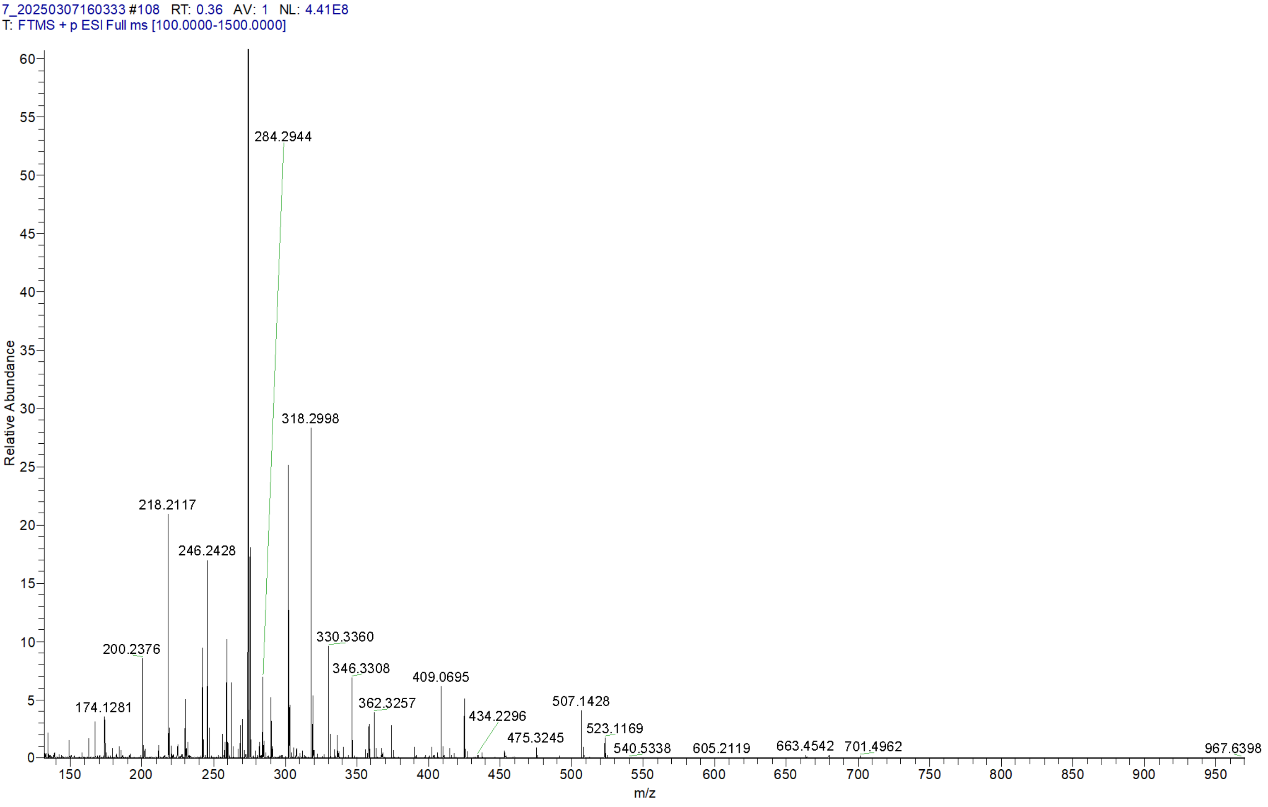


**Figure S83**. HRMS Spectrum of compound *2-fluoroethyl (2-(2-(trifluoromethoxy)phenoxy)cyclohexyl) sulfite* (**5.27**)


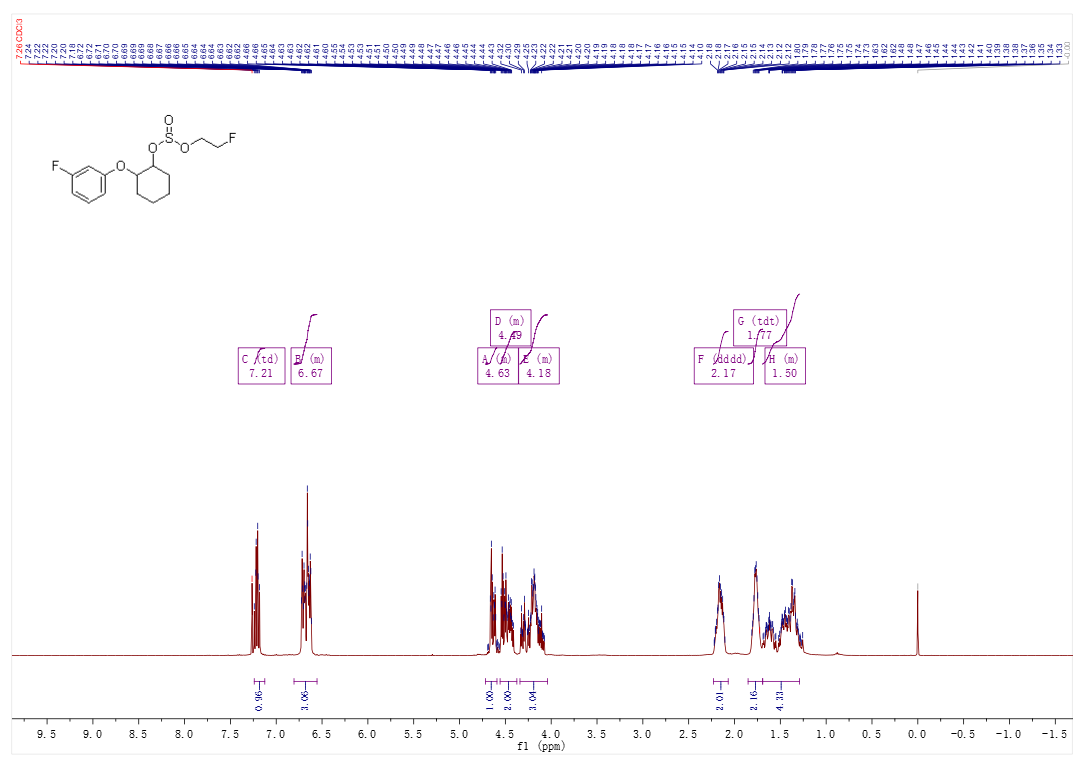


**Figure S84**. The ^1^H NMR spectrum of compound *2-fluoroethyl (2-(3-fluorophenoxy)cyclohexyl) sulfite* (**5.28**)


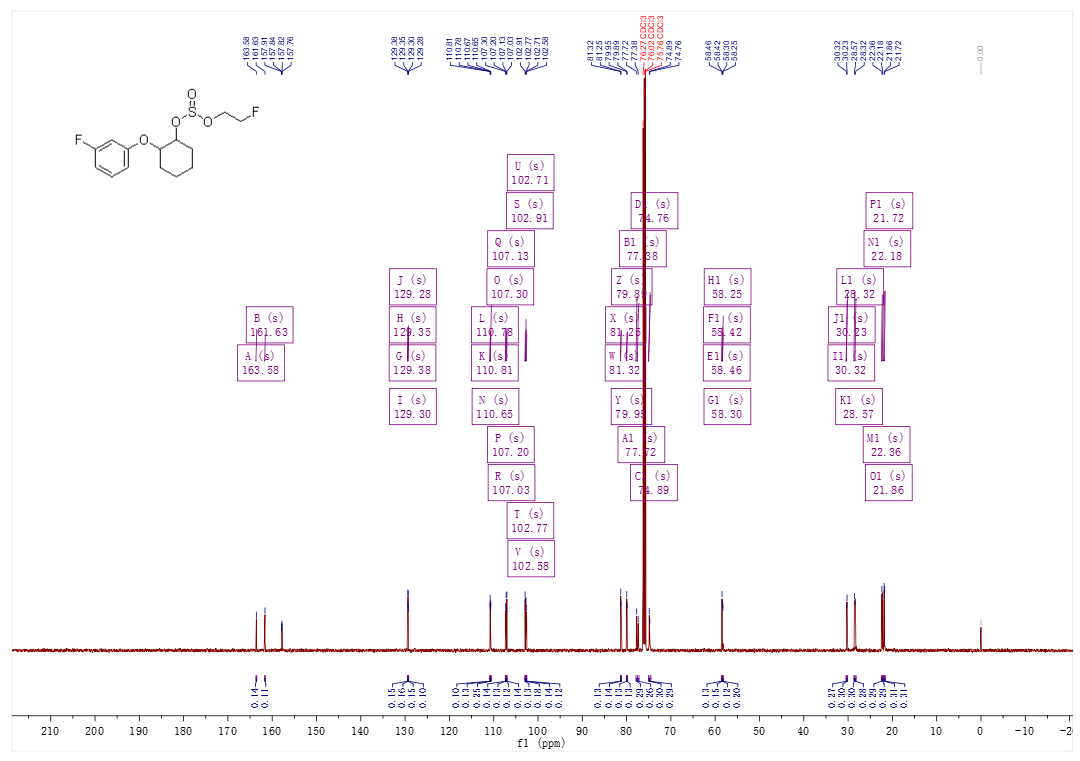


**Figure S85**. The ^13^C NMR spectrum of compound *2-fluoroethyl (2-(3-fluorophenoxy)cyclohexyl) sulfite* (**5.28**)


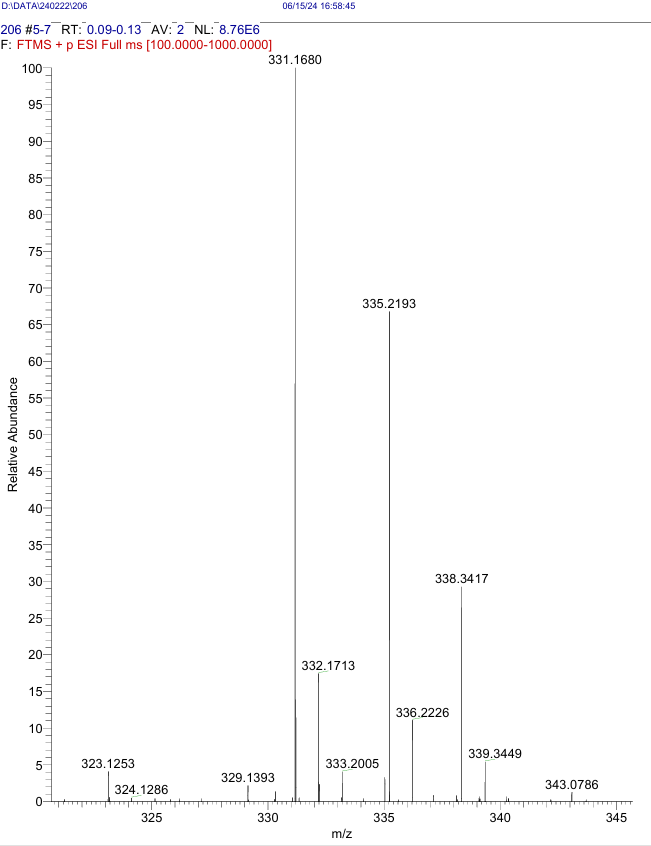


**Figure S86**. HRMS Spectrum of compound *2-fluoroethyl (2-(3-fluorophenoxy)cyclohexyl) sulfite* (**5.28**)


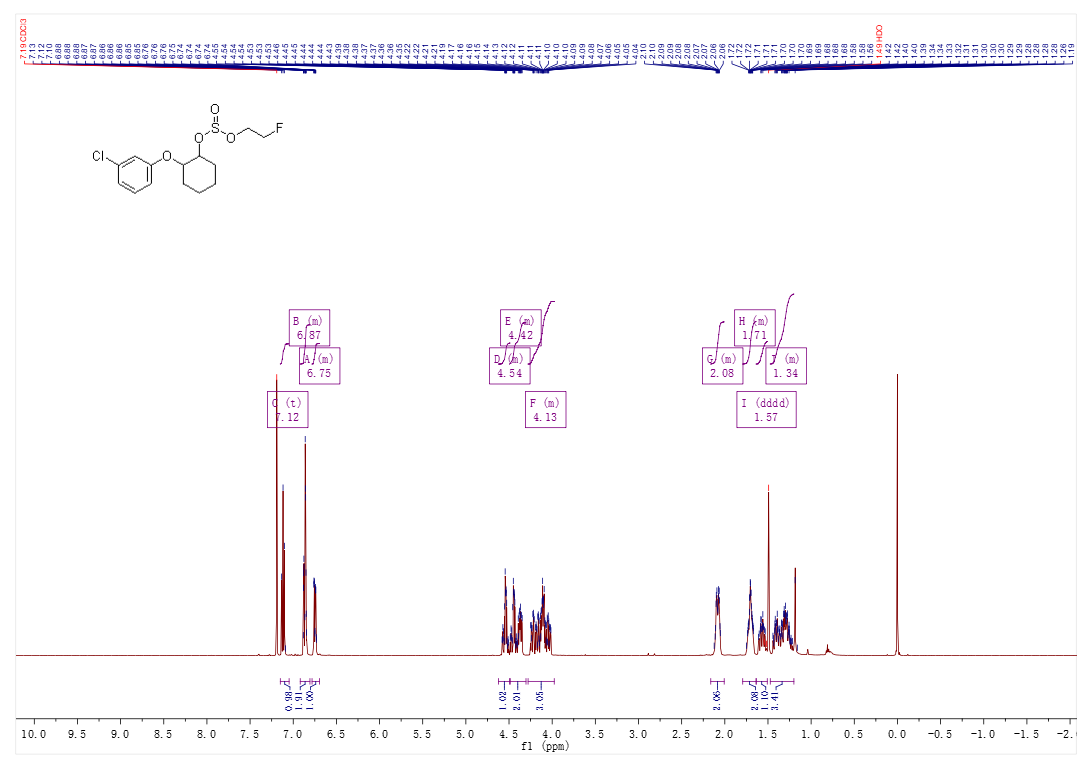


**Figure S87**. The ^1^H NMR spectrum of compound *2-(3-chlorophenoxy)cyclohexyl (2-fluoroethyl) sulfite* (**5.29**)


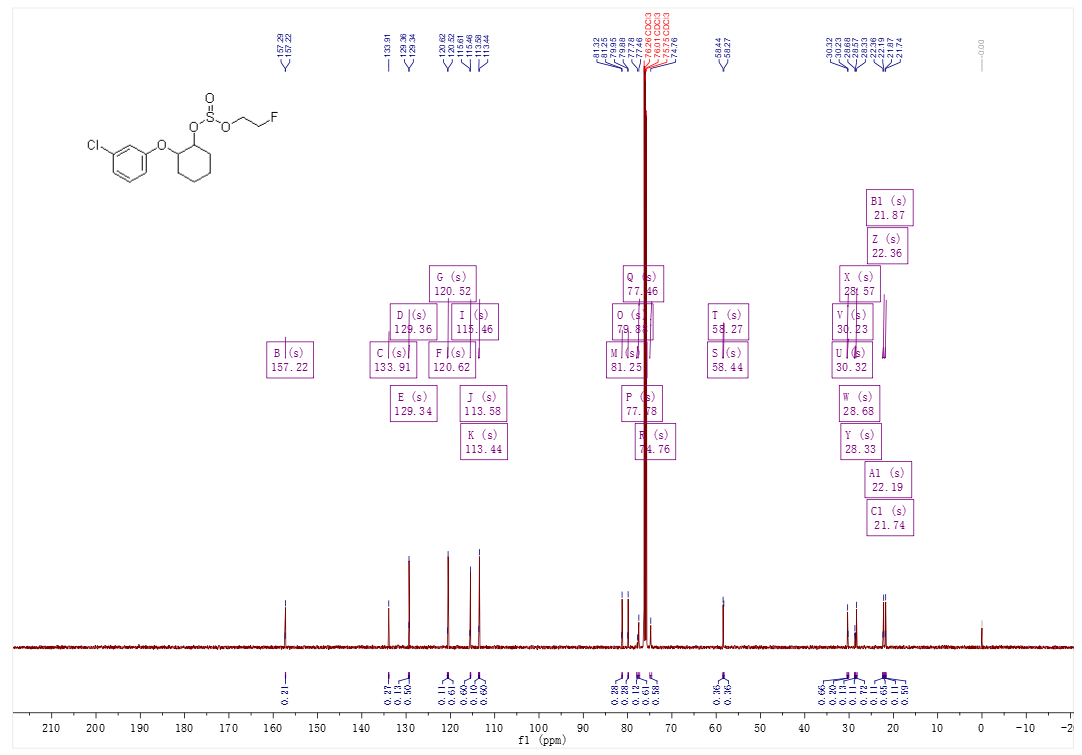


**Figure S88**. The ^13^C NMR spectrum of compound *2-(3-chlorophenoxy)cyclohexyl (2-fluoroethyl) sulfite* (**5.29**)


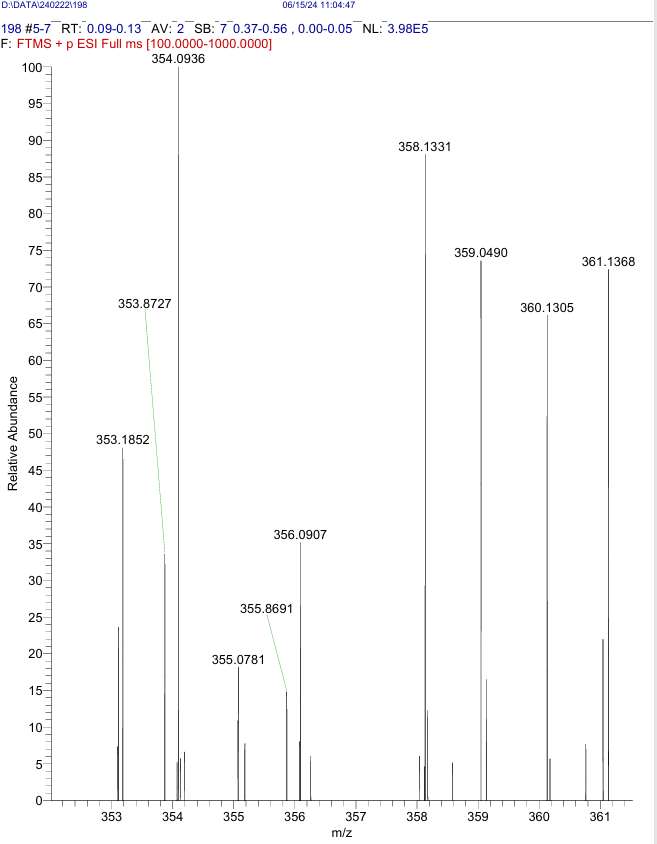


**Figure S89**. HRMS Spectrum of compound *2-(3-chlorophenoxy)cyclohexyl (2-fluoroethyl) sulfite* (**5.29**)


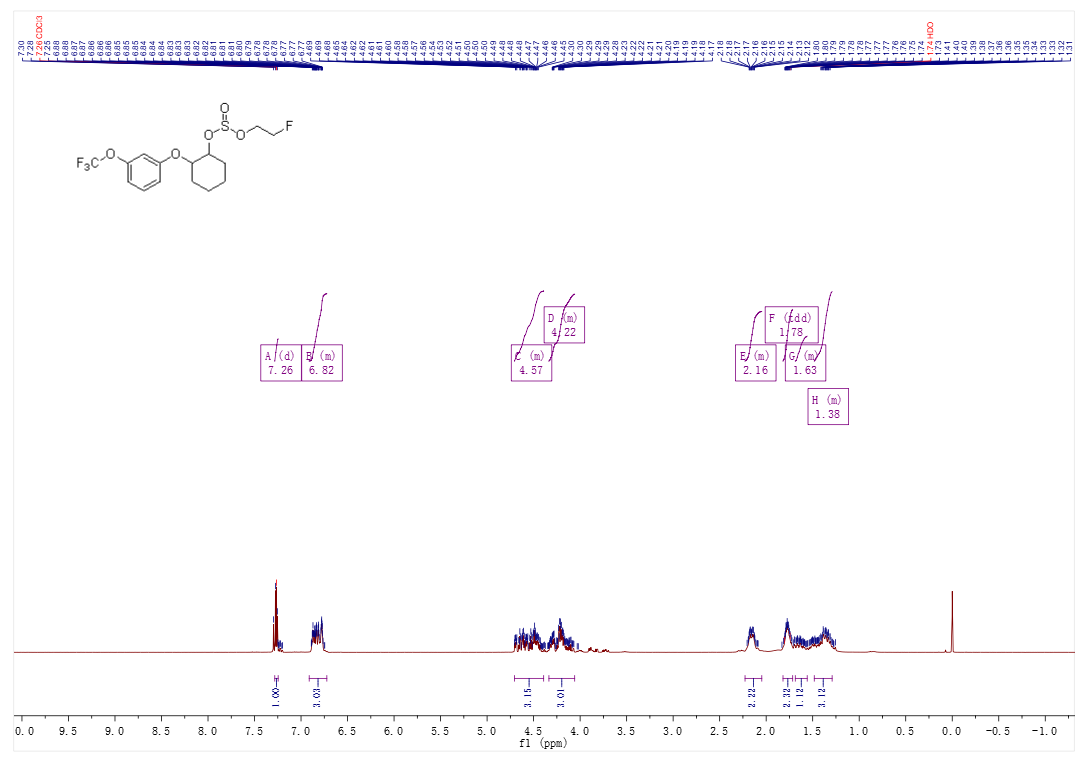


**Figure S90**. The ^1^H NMR spectrum of compound *2-fluoroethyl (2-(3-(trifluoromethoxy)phenoxy)cyclohexyl) sulfite* (**5.30**)


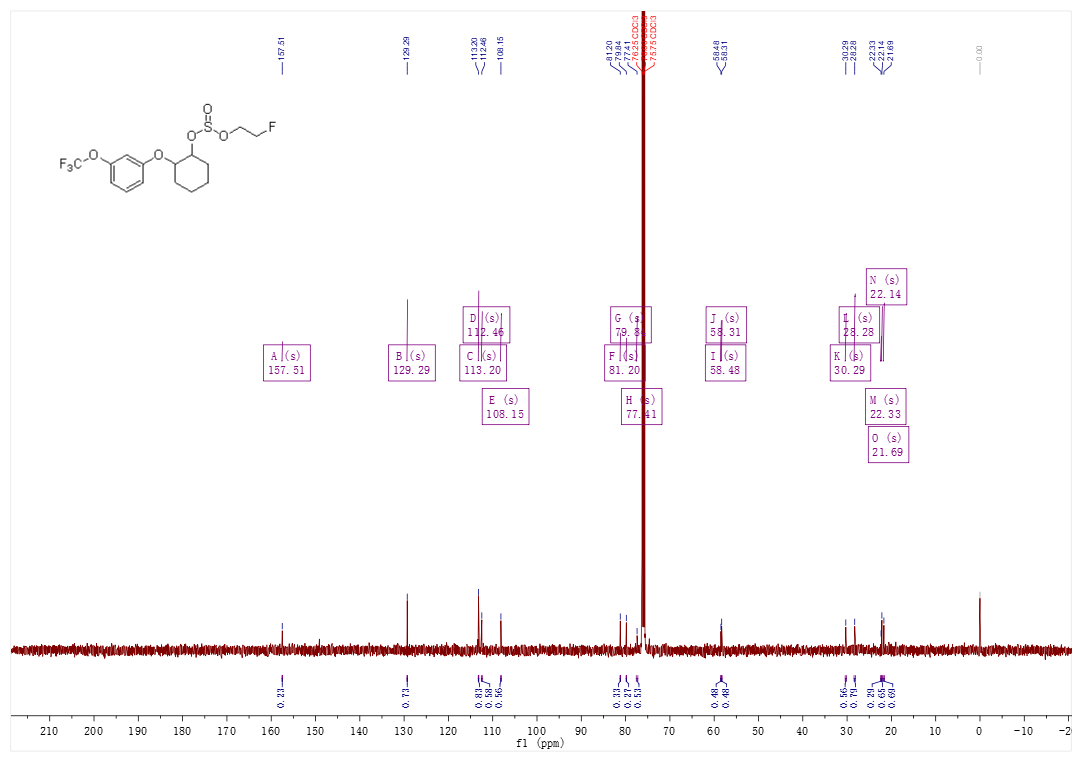


**Figure S91**. The ^13^C NMR spectrum of compound *2-fluoroethyl (2-(3-(trifluoromethoxy)phenoxy)cyclohexyl) sulfite* (**5.30**)


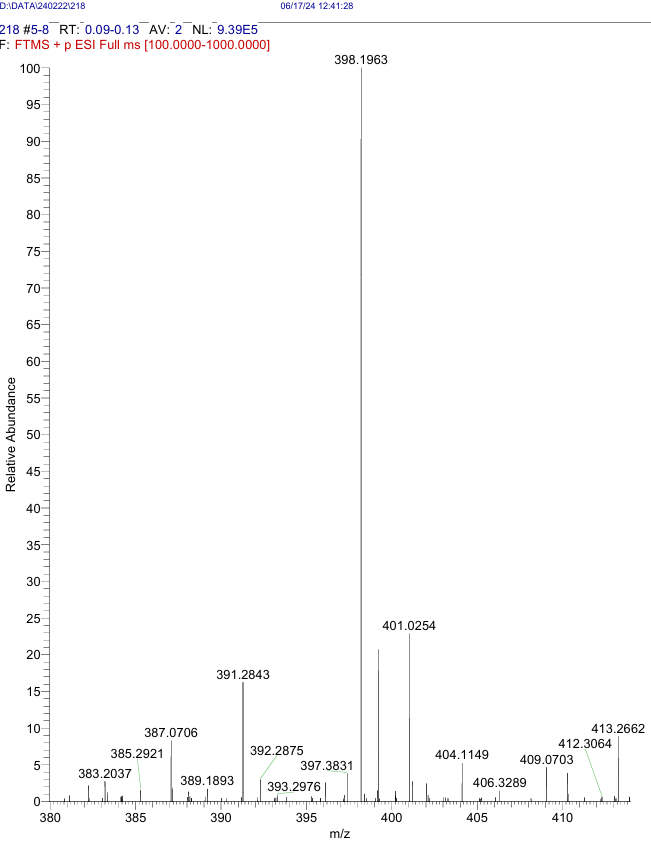


**Figure S92**. HRMS Spectrum of compound *2-fluoroethyl (2-(3-(trifluoromethoxy)phenoxy)cyclohexyl) sulfite* (**5.30**)


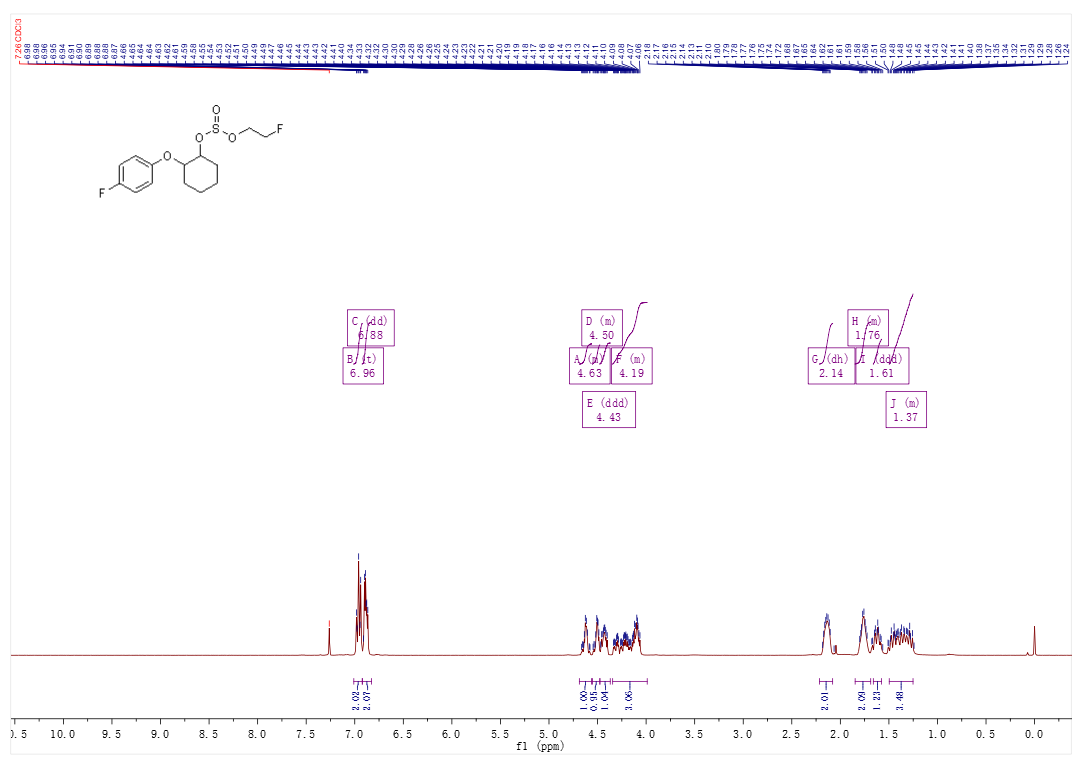


**Figure S93**. The ^1^H NMR spectrum of compound *2-fluoroethyl (2-(4-fluorophenoxy)cyclohexyl) sulfite* (**5.31**)


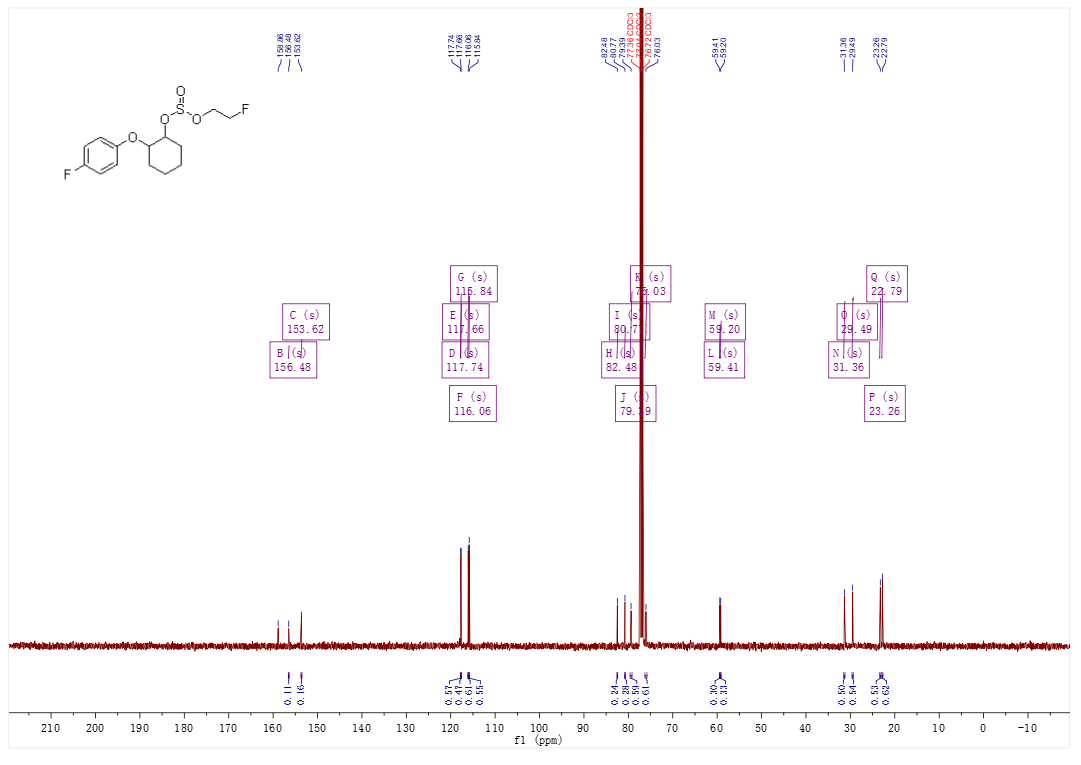


**Figure S94**. The ^13^C NMR spectrum of compound *2-fluoroethyl (2-(4-fluorophenoxy)cyclohexyl) sulfite* (**5.31**)


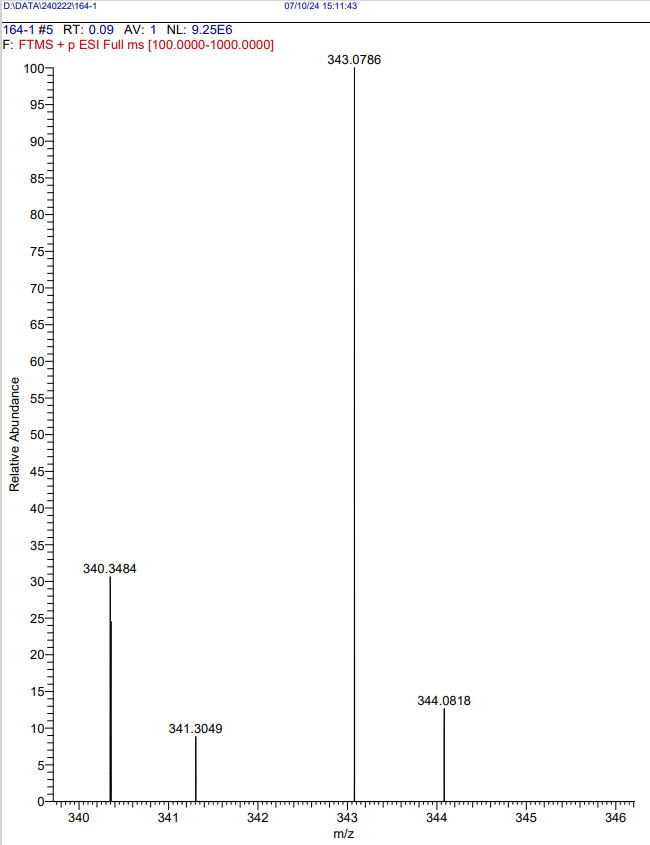


**Figure S95**. HRMS Spectrum of compound *2-fluoroethyl (2-(4-fluorophenoxy)cyclohexyl) sulfite* (**5.31**)
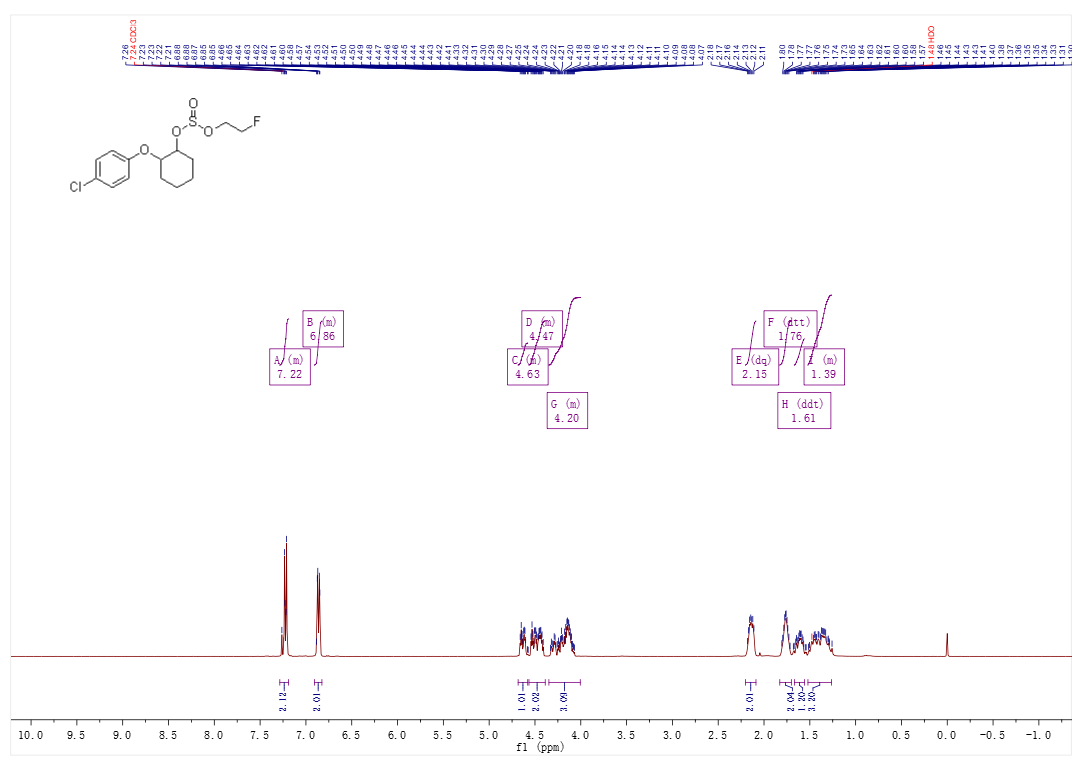


**Figure S96**. The ^1^H NMR spectrum of compound *2-(4-chlorophenoxy)cyclohexyl (2-fluoroethyl) sulfite* (**5.32**)


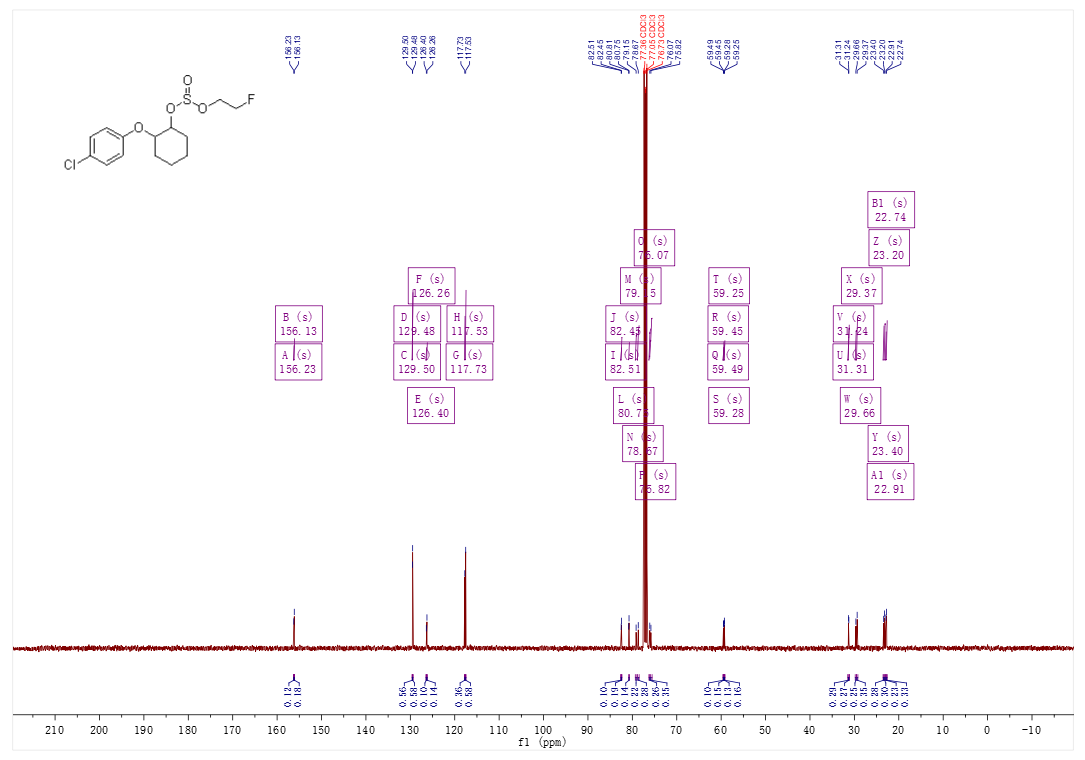


**Figure S97**. The ^13^C NMR spectrum of compound *2-(4-chlorophenoxy)cyclohexyl (2-fluoroethyl) sulfite* (**5.32**)


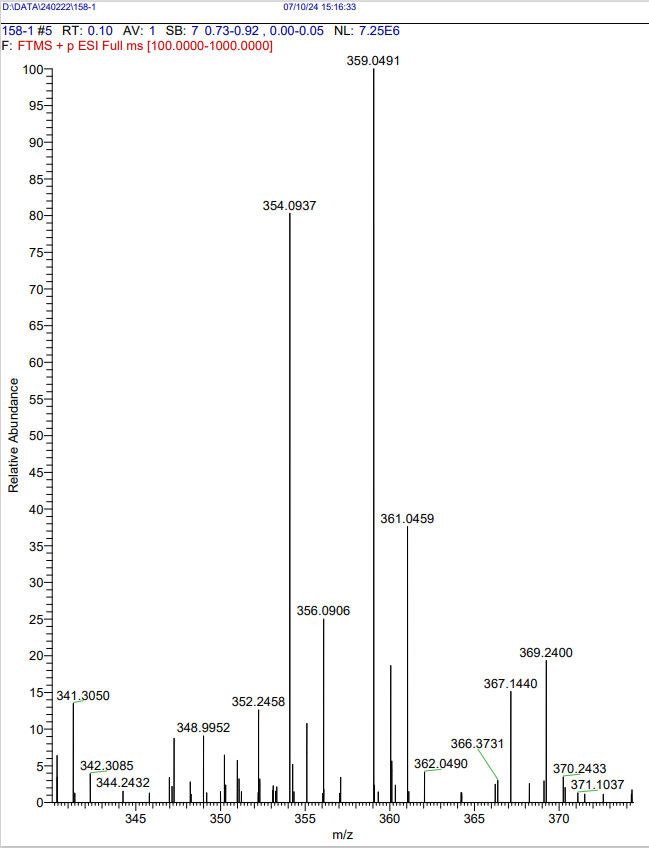


**Figure S98**. HRMS Spectrum of compound *2-(4-chlorophenoxy)cyclohexyl (2-fluoroethyl) sulfite* (**5.32**)


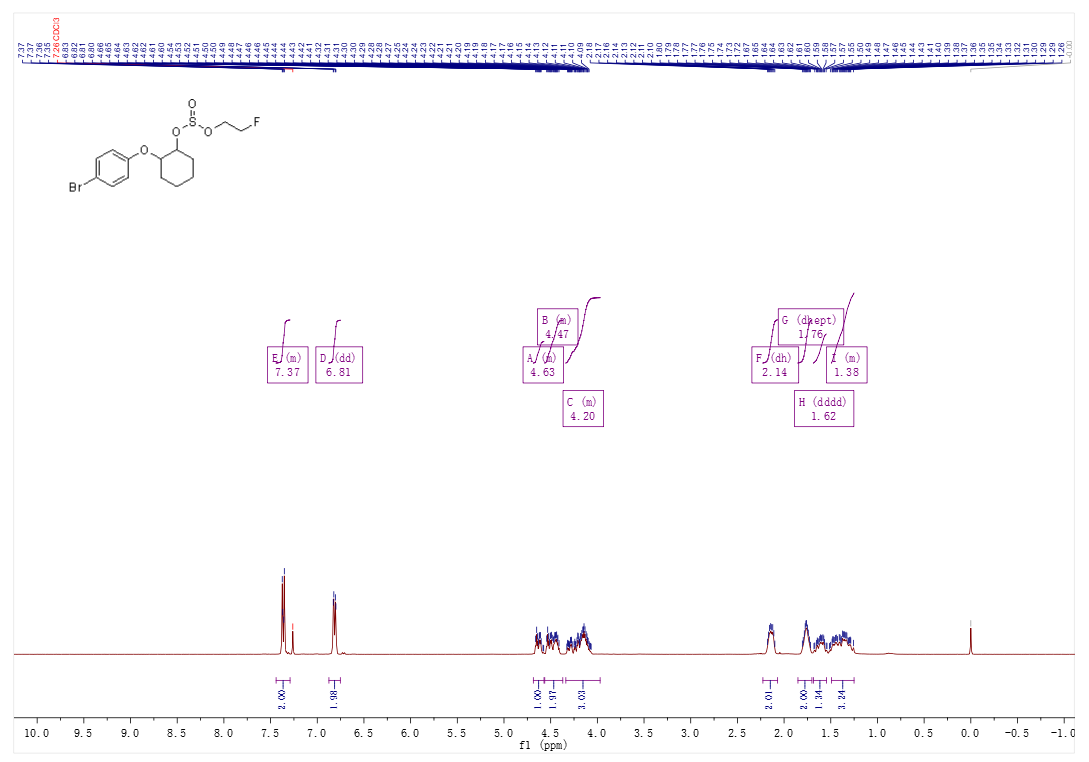


**Figure S99**. The ^1^H NMR spectrum of compound *2-(4-bromophenoxy)cyclohexyl (2-fluoroethyl) sulfite* (**5.33**)


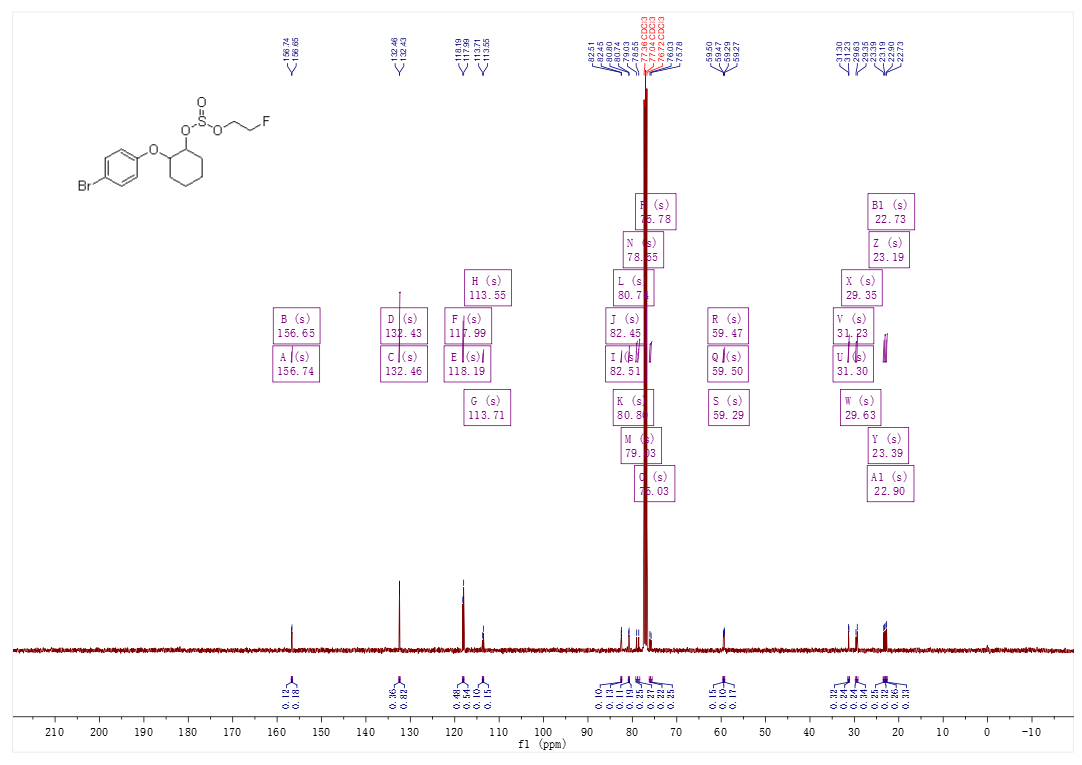


**Figure S100**. The ^13^C NMR spectrum of compound *2-(4-bromophenoxy)cyclohexyl (2-fluoroethyl) sulfite* (**5.33**)

**Figure S101**. HRMS Spectrum of compound *2-(4-bromophenoxy)cyclohexyl (2-fluoroethyl) sulfite* (**5.33**)

**Figure S102**. The ^1^H NMR spectrum of compound *2-fluoroethyl (2-(4-(trifluoromethoxy)phenoxy)cyclohexyl) sulfite* (**5.34**)

**Figure S103**. The ^13^C NMR spectrum of compound *2-fluoroethyl (2-(4-(trifluoromethoxy)phenoxy)cyclohexyl) sulfite* (**5.34**)

**Figure S104**. HRMS Spectrum of compound *2-fluoroethyl (2-(4-(trifluoromethoxy)phenoxy)cyclohexyl) sulfite* (**5.34**)

**Figure S105**. The ^1^H NMR spectrum of compound *2-fluoroethyl (2-(4-((trifluoromethyl)thio)phenoxy)cyclohexyl) sulfite* (**5.35**)

**Figure S106**. The ^13^C NMR spectrum of compound *2-fluoroethyl (2-(4-((trifluoromethyl)thio)phenoxy)cyclohexyl) sulfite* (**5.35**)

**Figure S107**. HRMS Spectrum of compound *2-fluoroethyl (2-(4-((trifluoromethyl)thio)phenoxy)cyclohexyl) sulfite* (**5.35**)

**Figure S108**. The ^1^H NMR spectrum of compound *2-fluoroethyl (2-(4-(2,2,2-trifluoroethoxy)phenoxy)cyclohexyl) sulfite* (**5.36**)

**Figure S109**. The ^13^C NMR spectrum of compound *2-fluoroethyl (2-(4-(2,2,2-trifluoroethoxy)phenoxy)cyclohexyl) sulfite* (**5.36**)

**Figure S110**. HRMS Spectrum of compound *2-fluoroethyl (2-(4-(2,2,2-trifluoroethoxy)phenoxy)cyclohexyl) sulfite* (**5.36**)
