# Supplementary material for: Design, synthesis, and biological activity of novel halogenated sulfite compounds
Source: PLoS One. 2025 Jul 2;20(7):e0327587. doi: 10.1371/journal.pone.0327587 (PMC12220988; doi:10.1371/journal.pone.0327587)
Supplement: S2 File — (DOCX) [file pone.0327587.s002.docx]

**S2: The Original photos of cowpea seedlings treated with compounds and CK**


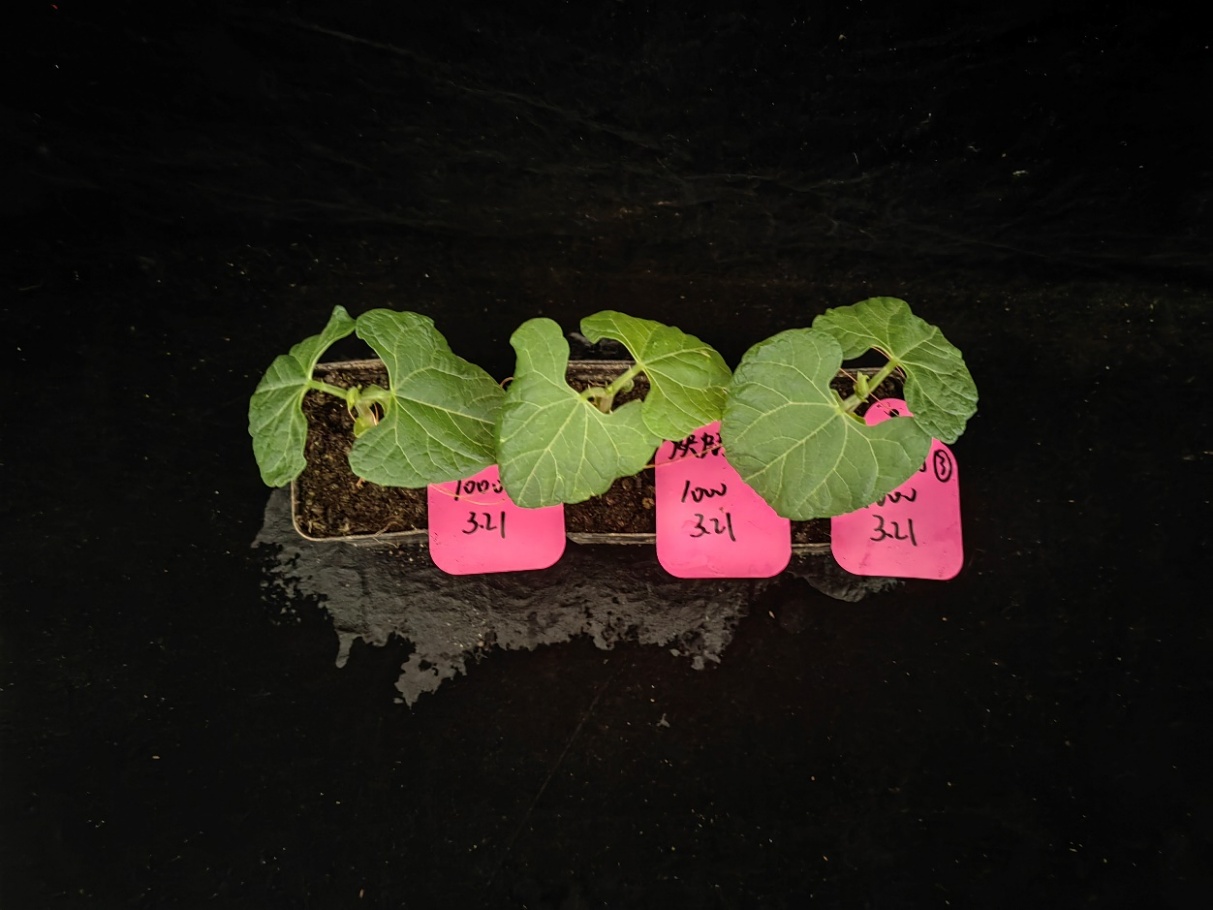


**Figure S111**. Safety of compound propargite on cowpea seedlings at mass concentrations of 1000 mg/L


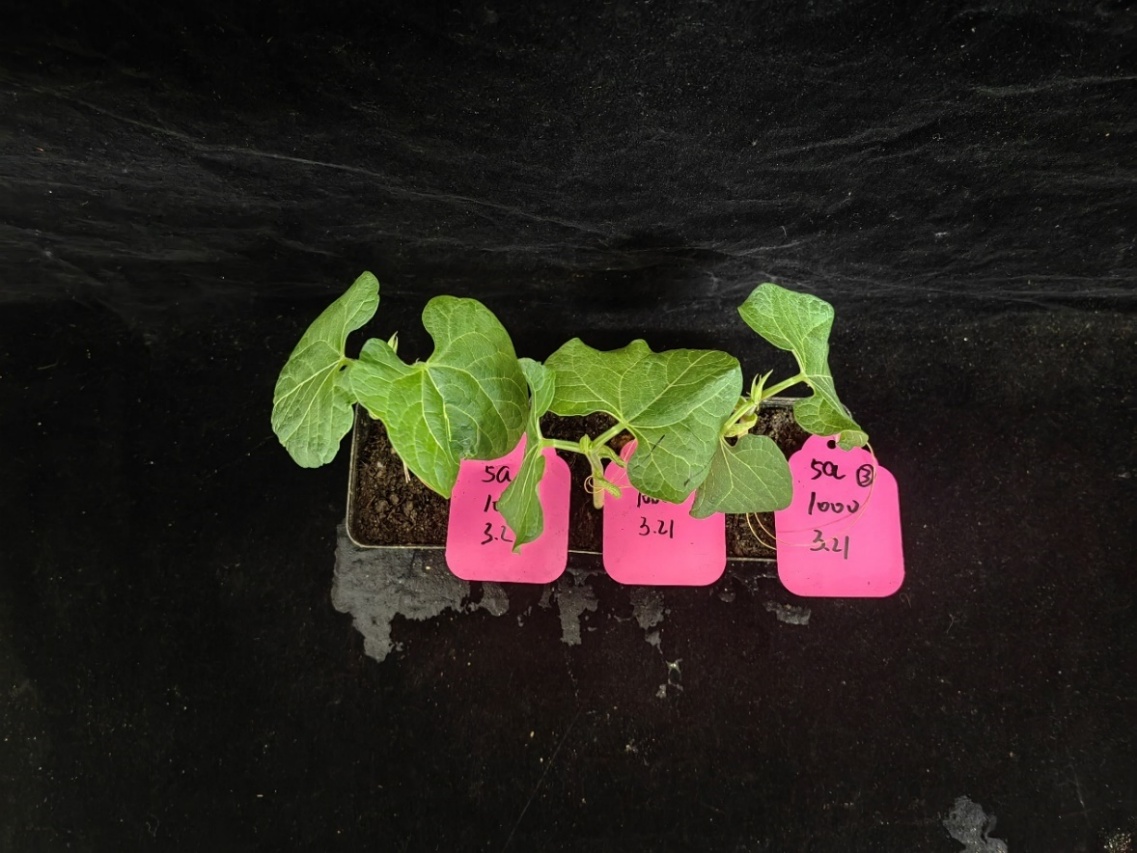


**Figure S112**. Safety of compound *2-(4-(tert-butyl)phenoxy)cyclohexyl (2-fluoroethyl) sulfite* (**5.16**) on cowpea seedlings at mass concentrations of 1000 mg/L


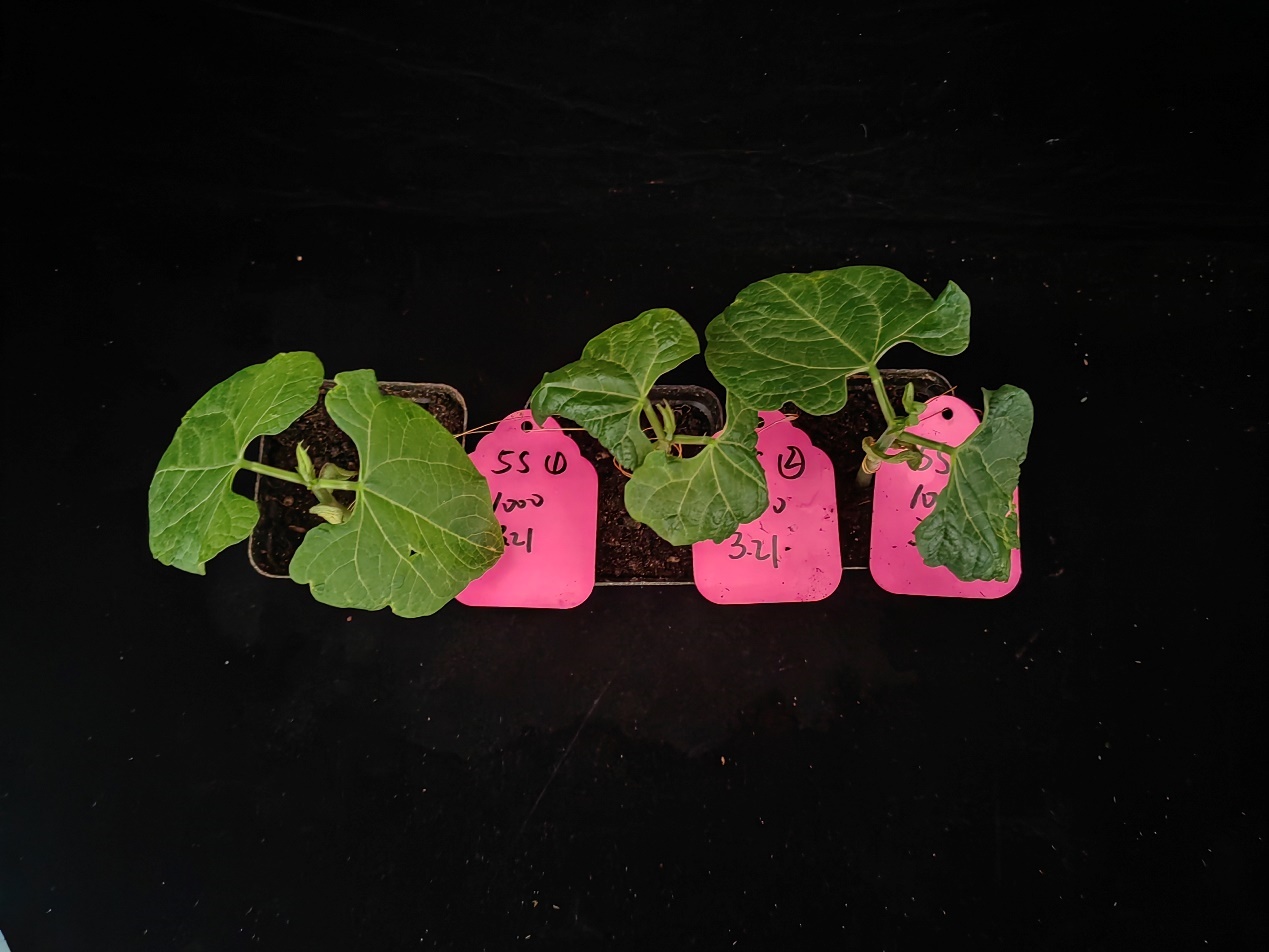


**Figure S113**. Safety of compound *2-(4-chlorophenoxy)cyclohexyl (2-fluoroethyl) sulfite* (**5.32**) on cowpea seedlings at mass concentrations of 1000 mg/L


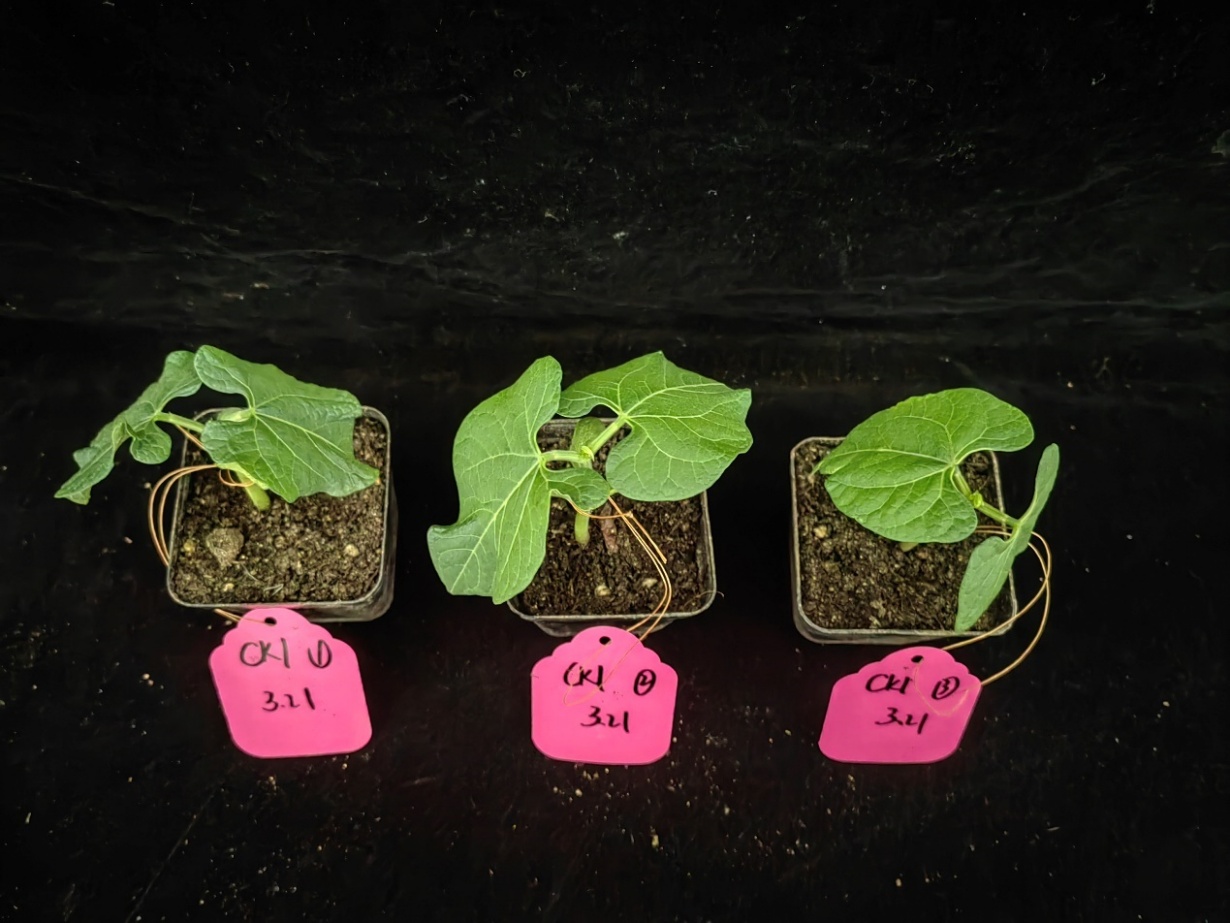


**Figure S114**. Safety of CK on cowpea seedlings


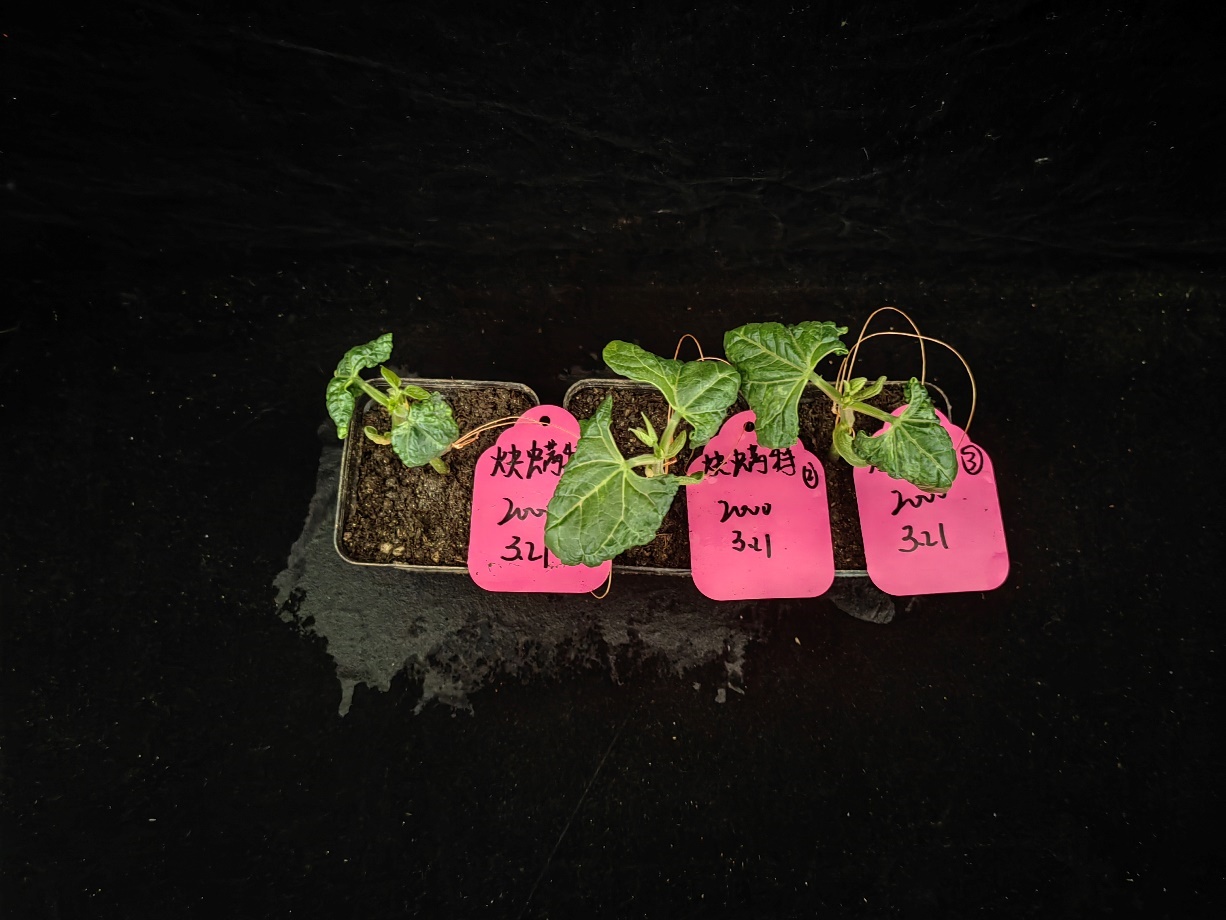


**Figure S115**. Safety of compound propargite on cowpea seedlings at mass concentrations of 2000 mg/L


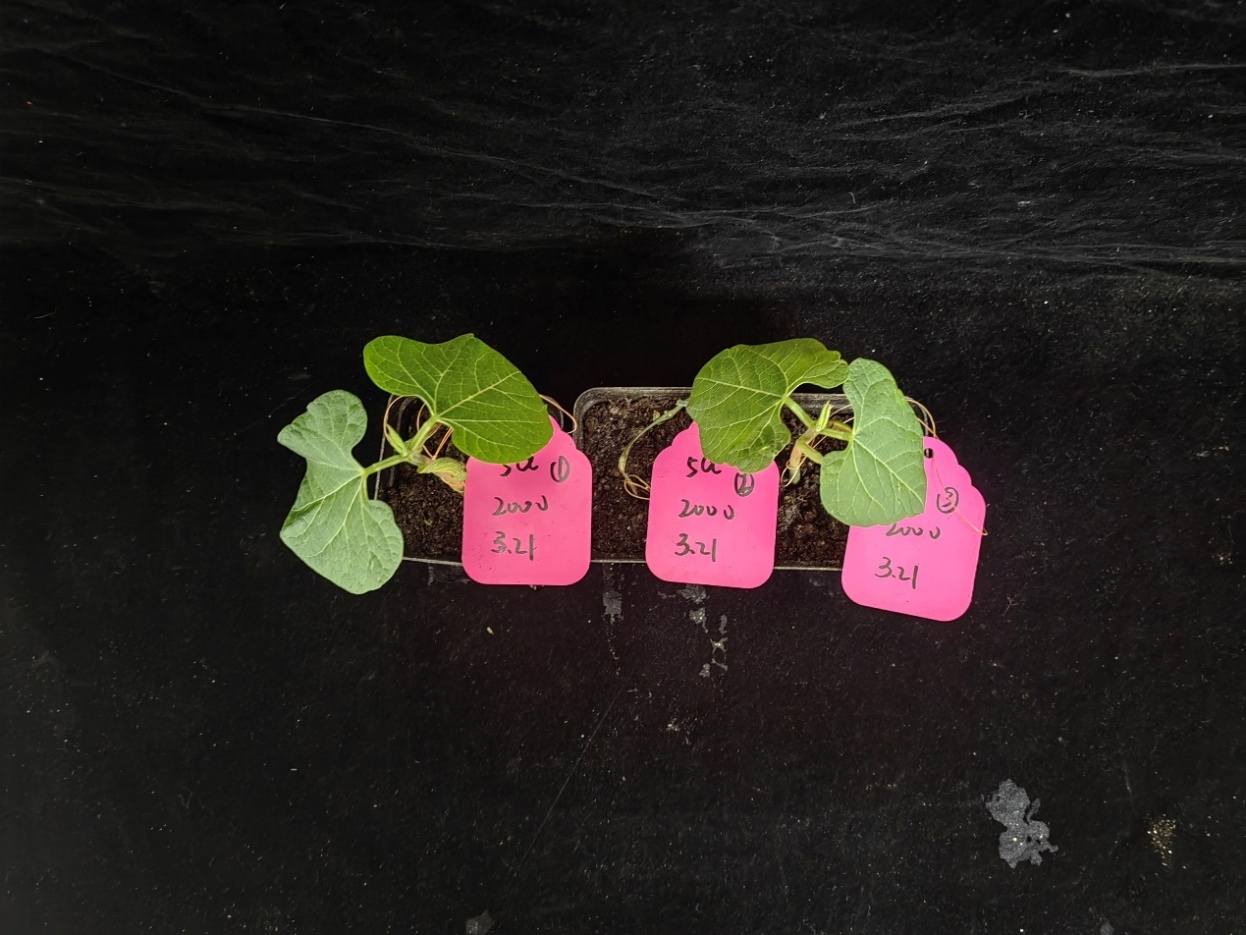


**Figure S116**. Safety of compound *2-(4-(tert-butyl)phenoxy)cyclohexyl (2-fluoroethyl) sulfite* (**5.16**) on cowpea seedlings at mass concentrations of 2000 mg/L


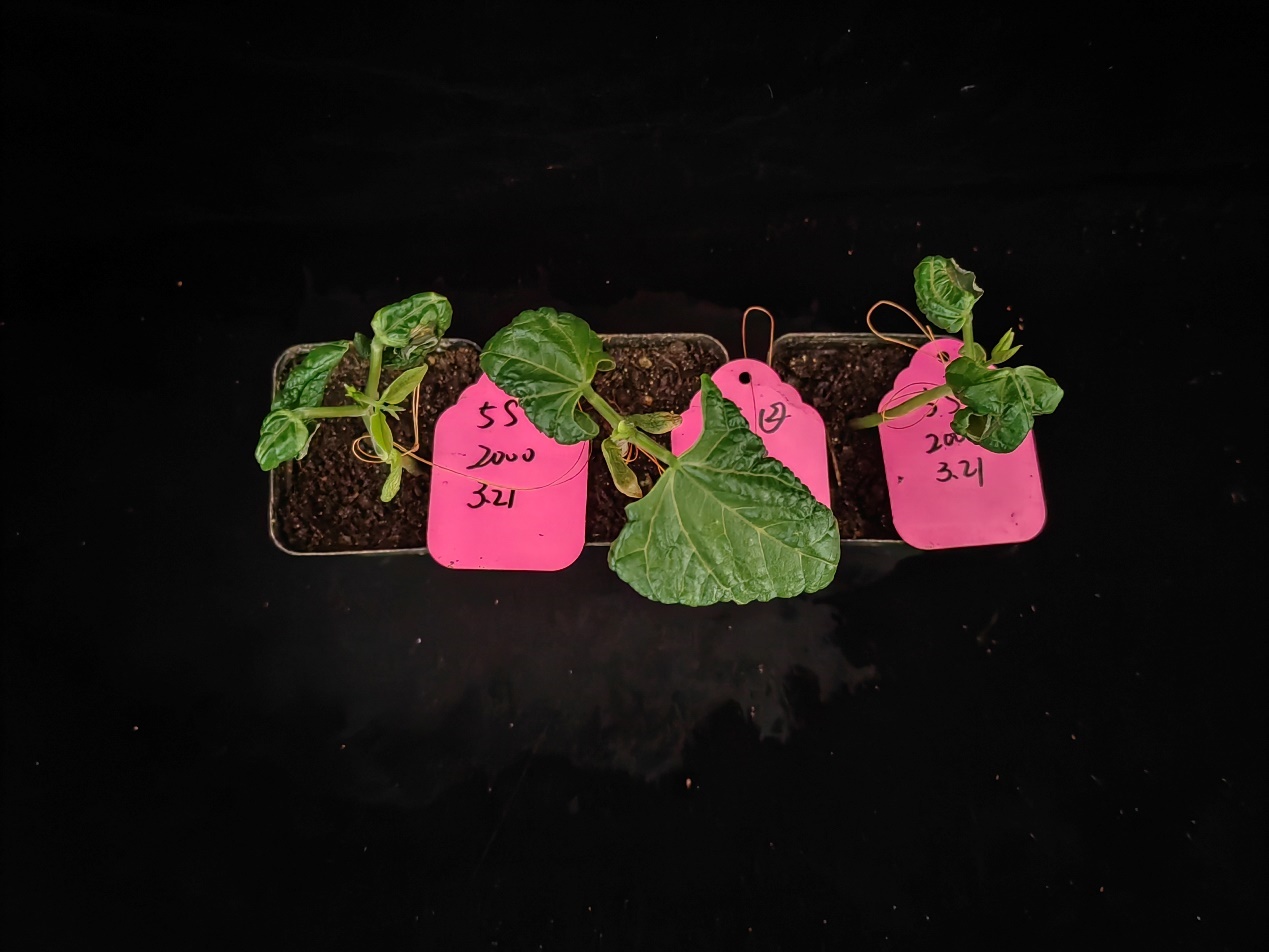


**Figure S117**. Safety of compound *2-(4-chlorophenoxy)cyclohexyl (2-fluoroethyl) sulfite* (**5.32**) on cowpea seedlings at mass concentrations of 2000 mg/L
