# Supplementary material for: Design, synthesis, and biological activity of novel halogenated sulfite compounds
Source: PLoS One. 2025 Jul 2;20(7):e0327587. doi: 10.1371/journal.pone.0327587 (PMC12220988; doi:10.1371/journal.pone.0327587)
Supplement: S3 File — (DOCX) [file pone.0327587.s003.docx]

**Structure file for each compound**

| Compound | Structure | Compound | Structure |
| --- | --- | --- | --- |
| **5.01** |  | **5.02** |  |
| **5.03** |  | **5.04** |  |
| **5.05** |  | **5.06** |  |
| **5.07** |  | **5.08** |  |
| **5.09** |  | **5.10** |  |
| **5.11** |  | **5.12** |  |
| **5.13** |  | **5.14** |  |
| **5.15** |  | **5.16** |  |

| **5.17** |  | **5.18** |  |
| --- | --- | --- | --- |
| **5.19** |  | **5.20** |  |
| **5.21** |  | **5.22** |  |
| **5.23** |  | **5.24** |  |

| **5.25** |  | **5.26** |  |
| --- | --- | --- | --- |
| **5.27** |  | **5.28** |  |
| **5.29** |  | **5.30** |  |
| **5.31** |  | **5.32** |  |
| **5.33** |  | **5.34** |  |
| **5.35** |  | **5.36** |  |
