# Supplementary figures and images for: Design, synthesis, and biological activity of novel halogenated sulfite compounds
Source: PLoS One. 2025 Jul 2;20(7):e0327587. doi: 10.1371/journal.pone.0327587 (PMC12220988; doi:10.1371/journal.pone.0327587)

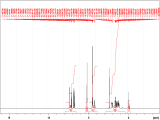

Supplement: S4 File — (ZIP) [file pone.0327587.s004.zip › The primary NMR data files-0524/2-(2-bromophenoxy)cyclohexyl prop-2-yn-1-yl sulfite (5.03)-HNMR/pdata/1/thumb.png]

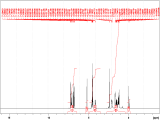

Supplement: S4 File — (ZIP) [file pone.0327587.s004.zip › The primary NMR data files-0524/2-(2-chlorophenoxy)cyclohexyl prop-2-yn-1-yl sulfite (5.02)-HNMR/pdata/1/thumb.png]
