# Supplementary material for: Design, synthesis, and biological activity of novel halogenated sulfite compounds
Source: PLoS One. 2025 Jul 2;20(7):e0327587. doi: 10.1371/journal.pone.0327587 (PMC12220988; doi:10.1371/journal.pone.0327587)
Supplement: S4 File — (ZIP) [file pone.0327587.s004.zip › The primary NMR data files-0524/2-(3-bromophenoxy)cyclohexyl prop-2-yn-1-yl sulfite (5.08)-CNMR/pdata/1/email_JBY209-1_2_1.pdf]

JBY209-1 13C

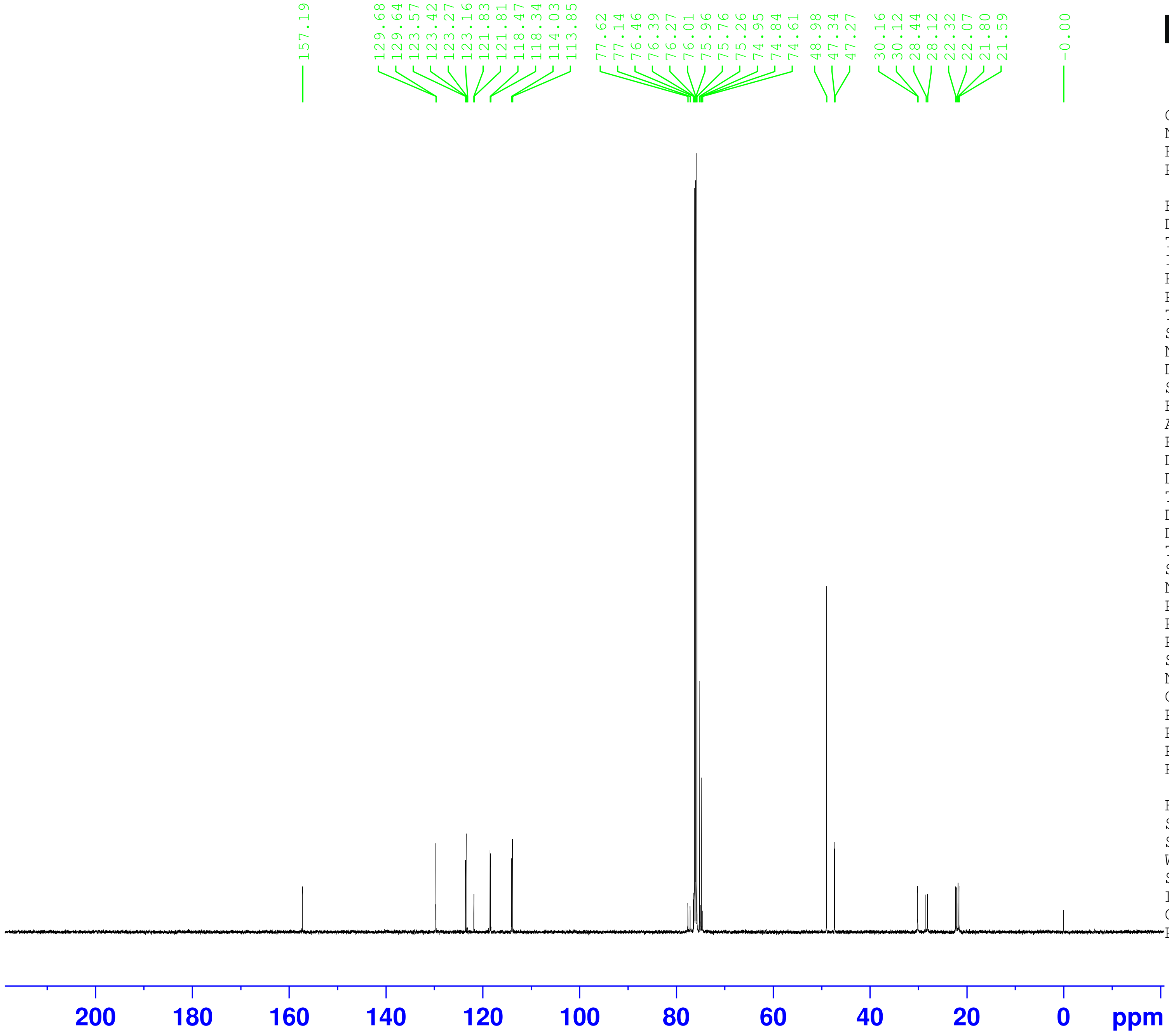

BRUKER

Current Data Parameters
NAME JBY209-1
EXPNO 2
PROCNO 1

F2 - Acquisition Parameters
Date_ 20240927
Time 0 h
INSTRUM Avance
PROBHD Z167419_0061 (
PULPROG zgpg30
TD 65536
SOLVENT CDCl3
NS 1024
DS 4
SWH 30120.482 Hz
FIDRES 0.919204 Hz
AQ 1.0878977 sec
RG 101
DW 16.600 usec
DE 6.50 usec
TE 298.0 K
D1 2.00000000 sec
D11 0.03000000 sec
TD0 1
SFO1 125.7779086 MHz
NUC1 13C
P0 3.00 usec
P1 9.00 usec
PLW1 97.08999634 W
SFO2 500.1620006 MHz
NUC2 1H
CPDPRG[2 waltz65
PCPD2 80.00 usec
PLW2 22.69700050 W
PLW12 0.22697000 W
PLW13 0.11417000 W

F2 - Processing parameters
SI 32768
SF 125.7654617 MHz
WDW EM
SSB 0
LB 1.00 Hz
GB 0
PC 1.40
