# Supplementary material for: Design, synthesis, and biological activity of novel halogenated sulfite compounds
Source: PLoS One. 2025 Jul 2;20(7):e0327587. doi: 10.1371/journal.pone.0327587 (PMC12220988; doi:10.1371/journal.pone.0327587)
Supplement: S4 File — (ZIP) [file pone.0327587.s004.zip › The primary NMR data files-0524/2-(3-bromophenoxy)cyclohexyl prop-2-yn-1-yl sulfite (5.08)-HNMR/pdata/1/email_JBY209-1_1_1.pdf]

JBY209-1 1H

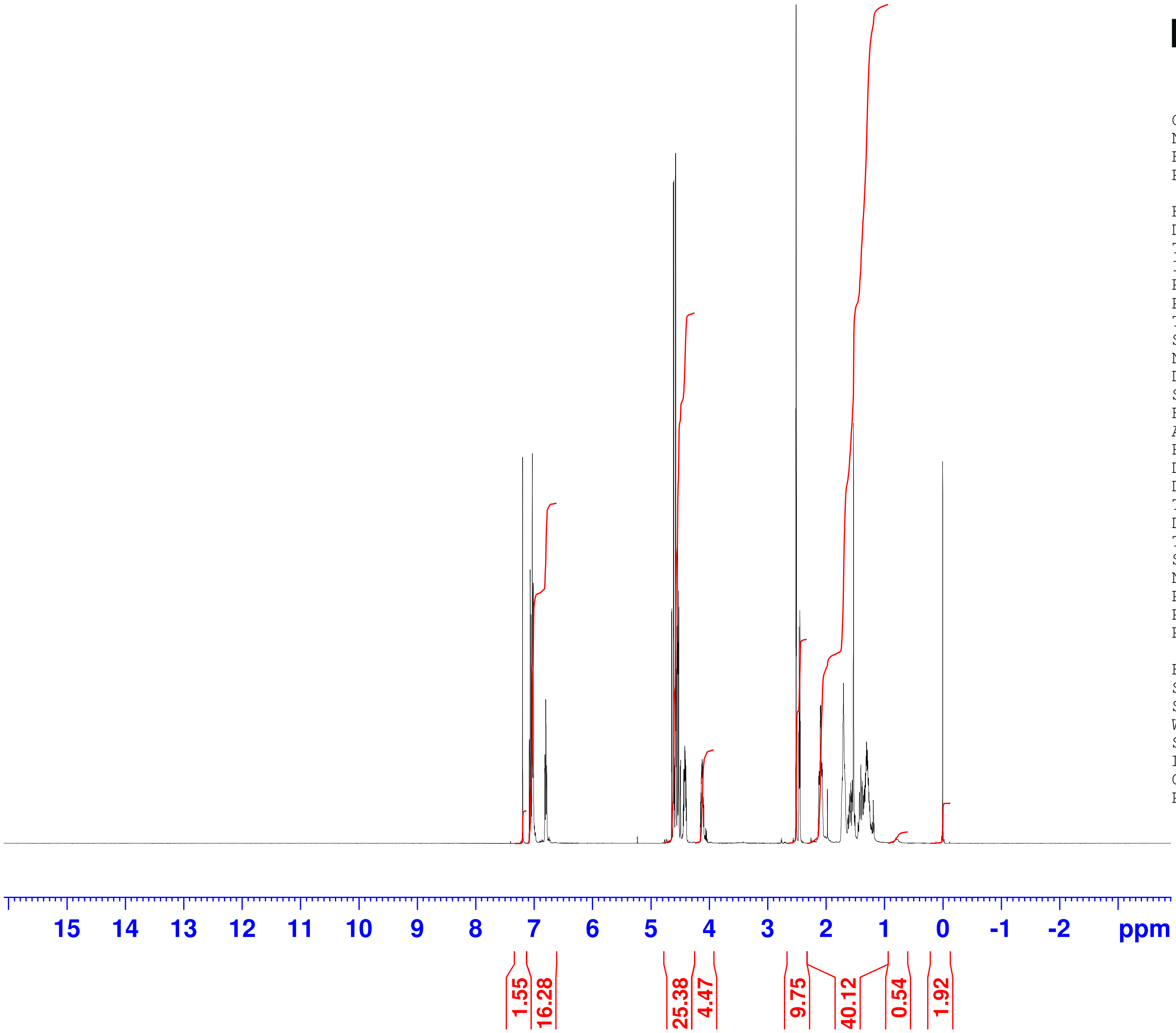

BRUKER

Current Data Parameters		
NAME	JBY209-1	
EXPNO	1	
PROCNO	1	
F2 - Acquisition Parameters		
Date_	20240926	
Time	23.05	h
INSTRUM	Avance	
PROBHD	Z167419_0061 (	
PULPROG	zg30	
TD	65536	
SOLVENT	CDCl3	
NS	16	
DS	2	
SWH	10000.000	Hz
FIDRES	0.305176	Hz
AQ	3.2767999	sec
RG	101	
DW	50.000	usec
DE	11.14	usec
TE	298.0	K
D1	1.00000000	sec
TD0	1	
SFO1	500.1630885	MHz
NUC1	1H	
P0	2.67	usec
P1	8.00	usec
PLW1	22.69700050	W
F2 - Processing parameters		
SI	65536	
SF	500.1600448	MHz
WDW	EM	
SSB	0	
LB	0.30	Hz
GB	0	
PC	1.00	
