# Supplementary material for: Design, synthesis, and biological activity of novel halogenated sulfite compounds
Source: PLoS One. 2025 Jul 2;20(7):e0327587. doi: 10.1371/journal.pone.0327587 (PMC12220988; doi:10.1371/journal.pone.0327587)
Supplement: S4 File — (ZIP) [file pone.0327587.s004.zip › The primary NMR data files-0524/2-fluoroethyl (2-(2-(trifluoromethoxy)phenoxy)cyclohexyl) sulfite (5.27)-HNMR/pdata/1/email_JBY212-1_1_1.pdf]

JBY212-1 1H 240919

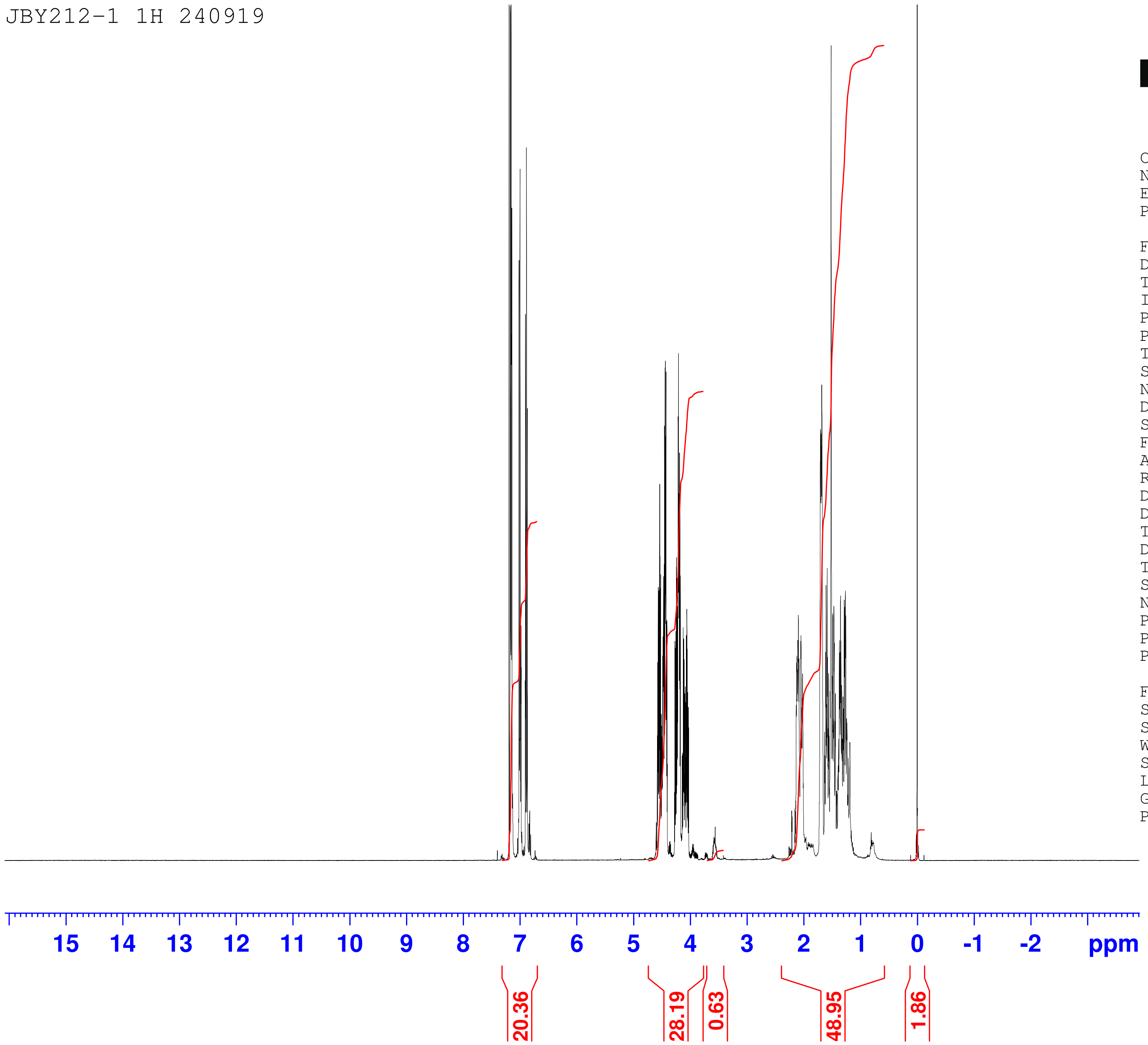

Current Data Parameters

NAME	JBY212-1
EXPNO	1
PROCNO	1

F2 - Acquisition Parameters

Date_	20240919
Time	21.54 h
INSTRUM	Avance
PROBHD	Z167419_0061 (
PULPROG	zg30
TD	65536
SOLVENT	CDCl3
NS	16
DS	2
SWH	10000.000 Hz
FIDRES	0.305176 Hz
AQ	3.2767999 sec
RG	101
DW	50.000 usec
DE	11.14 usec
TE	298.0 K
D1	1.00000000 sec
TD0	1
SFO1	500.1630885 MHz
NUC1	1H
P0	2.67 usec
P1	8.00 usec
PLW1	22.69700050 W

F2 - Processing parameters

SI	65536
SF	500.1600471 MHz
WDW	EM
SSB	0
LB	0.30 Hz
GB	0
PC	1.00
