# Supplementary material for: Design, synthesis, and biological activity of novel halogenated sulfite compounds
Source: PLoS One. 2025 Jul 2;20(7):e0327587. doi: 10.1371/journal.pone.0327587 (PMC12220988; doi:10.1371/journal.pone.0327587)
Supplement: S4 File — (ZIP) [file pone.0327587.s004.zip › The primary NMR data files-0524/2-fluoroethyl (2-(2-fluorophenoxy)cyclohexyl) sulfite (5.25)-CNMR/pdata/1/email_JBY185-1_2_1.pdf]

JBY185-1 C

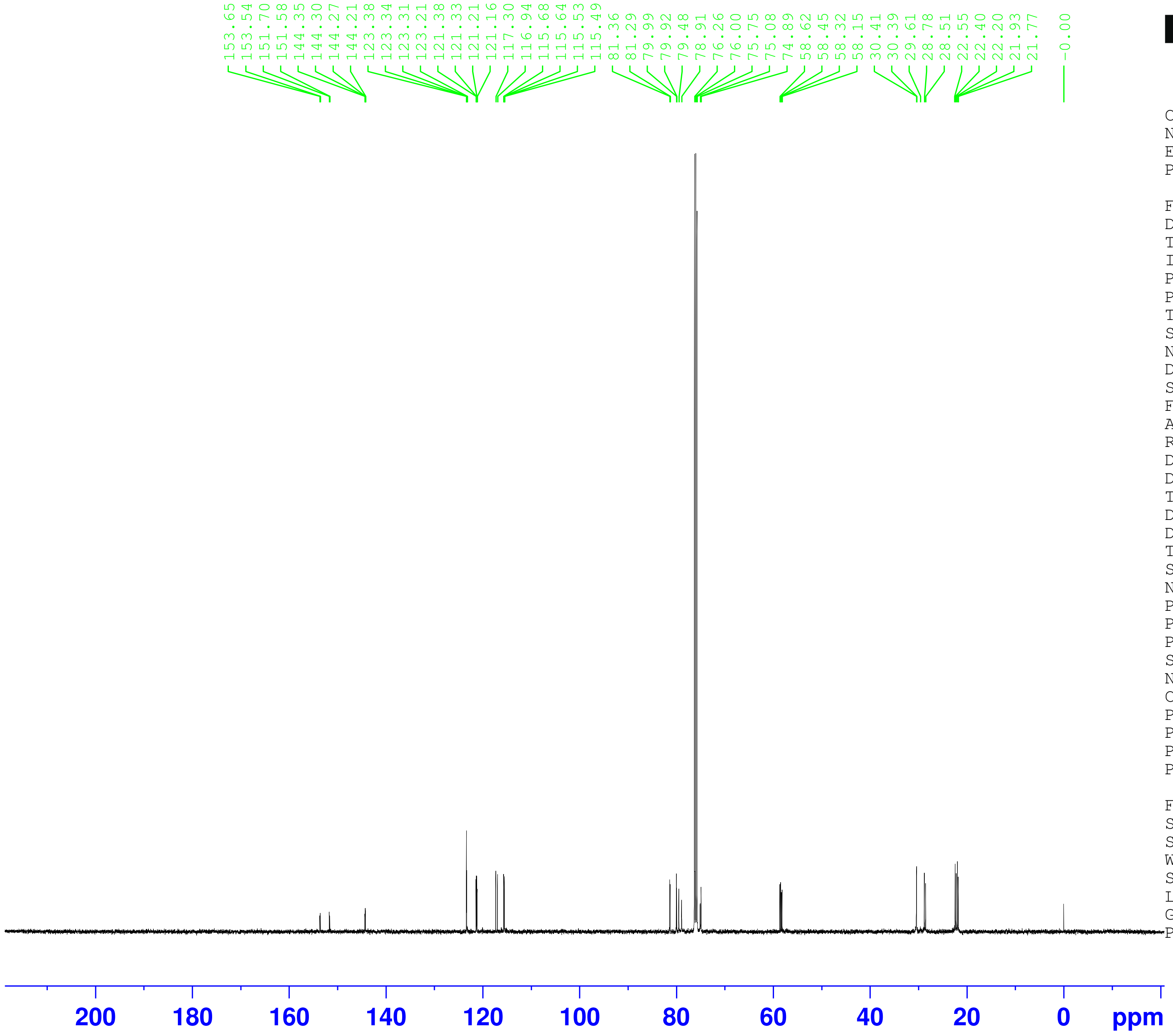

BRUKER

Current Data Parameters

NAME	JBY185-1
EXPNO	2
PROCNO	1

F2 - Acquisition Parameters

Date_	20240928
Time	6.45 h
INSTRUM	Avance
PROBHD	Z167419_0061 (
PULPROG	zgpg30
TD	65536
SOLVENT	CDCl3
NS	1024
DS	4
SWH	30120.482 Hz
FIDRES	0.919204 Hz
AQ	1.0878977 sec
RG	101
DW	16.600 usec
DE	6.50 usec
TE	298.0 K
D1	2.00000000 sec
D11	0.03000000 sec
TD0	1
SFO1	125.7779086 MHz
NUC1	13C
P0	3.00 usec
P1	9.00 usec
PLW1	97.08999634 W
SFO2	500.1620006 MHz
NUC2	1H
CPDPRG[2	waltz65
PCPD2	80.00 usec
PLW2	22.69700050 W
PLW12	0.22697000 W
PLW13	0.11417000 W

F2 - Processing parameters

SI	32768
SF	125.7654617 MHz
WDW	EM
SSB	0
LB	1.00 Hz
GB	0
PC	1.40
