# Supplementary material for: Design, synthesis, and biological activity of novel halogenated sulfite compounds
Source: PLoS One. 2025 Jul 2;20(7):e0327587. doi: 10.1371/journal.pone.0327587 (PMC12220988; doi:10.1371/journal.pone.0327587)
Supplement: S4 File — (ZIP) [file pone.0327587.s004.zip › The primary NMR data files-0524/2-fluoroethyl (2-(3-fluorophenoxy)cyclohexyl) sulfite (5.28)-HNMR/pdata/1/email_JBY206-1_1_1.pdf]

JBY206-1 1H

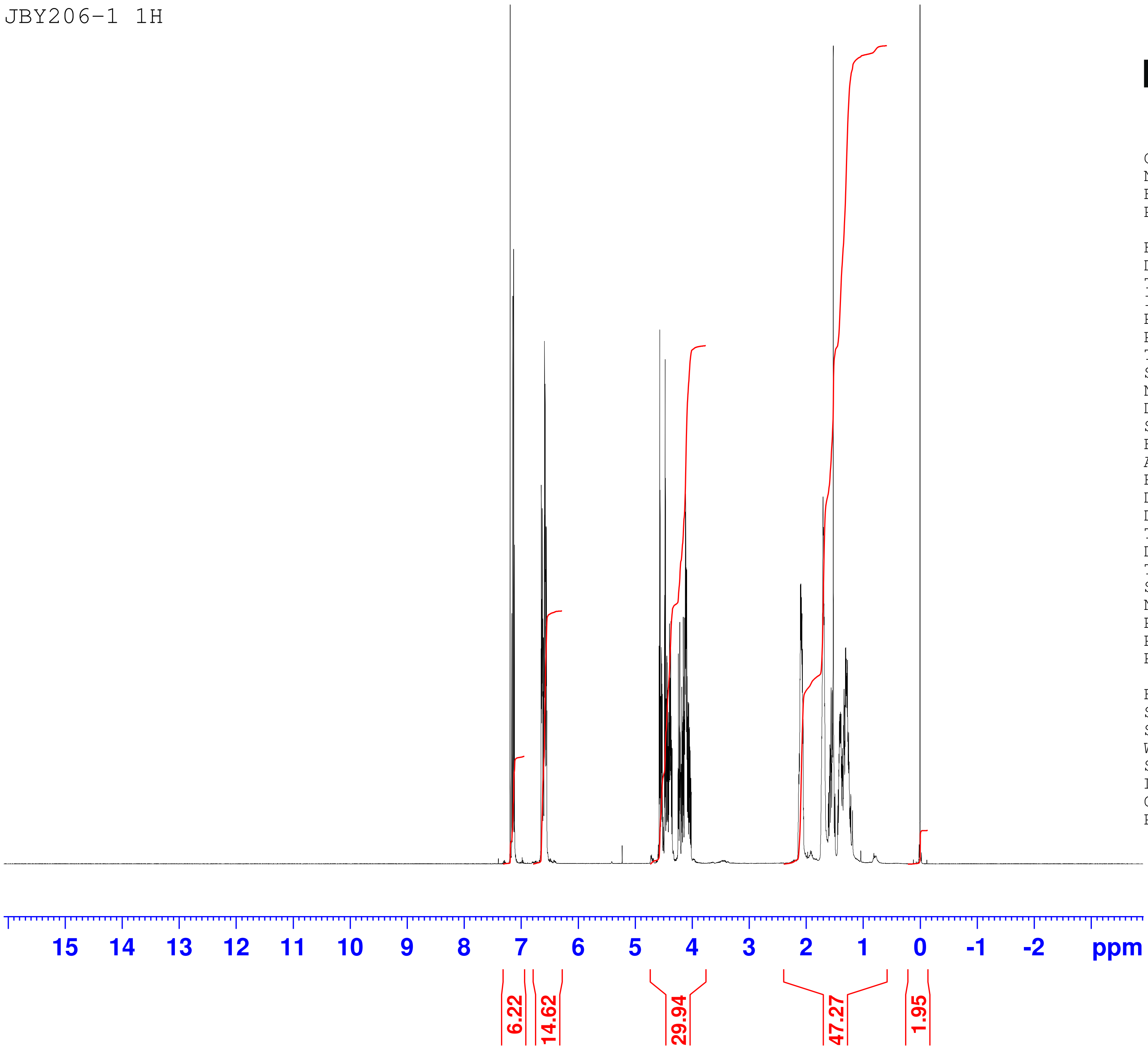

Current Data Parameters
NAME JBY206-1
EXPNO 1
PROCNO 1

F2 - Acquisition Parameters
Date_ 20240927
Time 2.02 h
INSTRUM Avance
PROBHD Z167419_0061 (
PULPROG zg30
TD 65536
SOLVENT CDCl3
NS 16
DS 2
SWH 10000.000 Hz
FIDRES 0.305176 Hz
AQ 3.2767999 sec
RG 101
DW 50.000 usec
DE 11.14 usec
TE 298.0 K
D1 1.00000000 sec
TD0 1
SFO1 500.1630885 MHz
NUC1 1H
P0 2.67 usec
P1 8.00 usec
PLW1 22.69700050 W

F2 - Processing parameters
SI 65536
SF 500.1600467 MHz
WDW EM
SSB 0
LB 0.30 Hz
GB 0
PC 1.00
