# Supplementary material for: Design, synthesis, and biological activity of novel halogenated sulfite compounds
Source: PLoS One. 2025 Jul 2;20(7):e0327587. doi: 10.1371/journal.pone.0327587 (PMC12220988; doi:10.1371/journal.pone.0327587)
Supplement: S4 File — (ZIP) [file pone.0327587.s004.zip › The primary NMR data files-0524/prop-2-yn-1-yl (2-(2-(trifluoromethoxy)phenoxy)cyclohexyl) sulfite (5.04)-CNMR/pdata/1/email_JBY221-1_2_1.pdf]

JBY221-1 13C

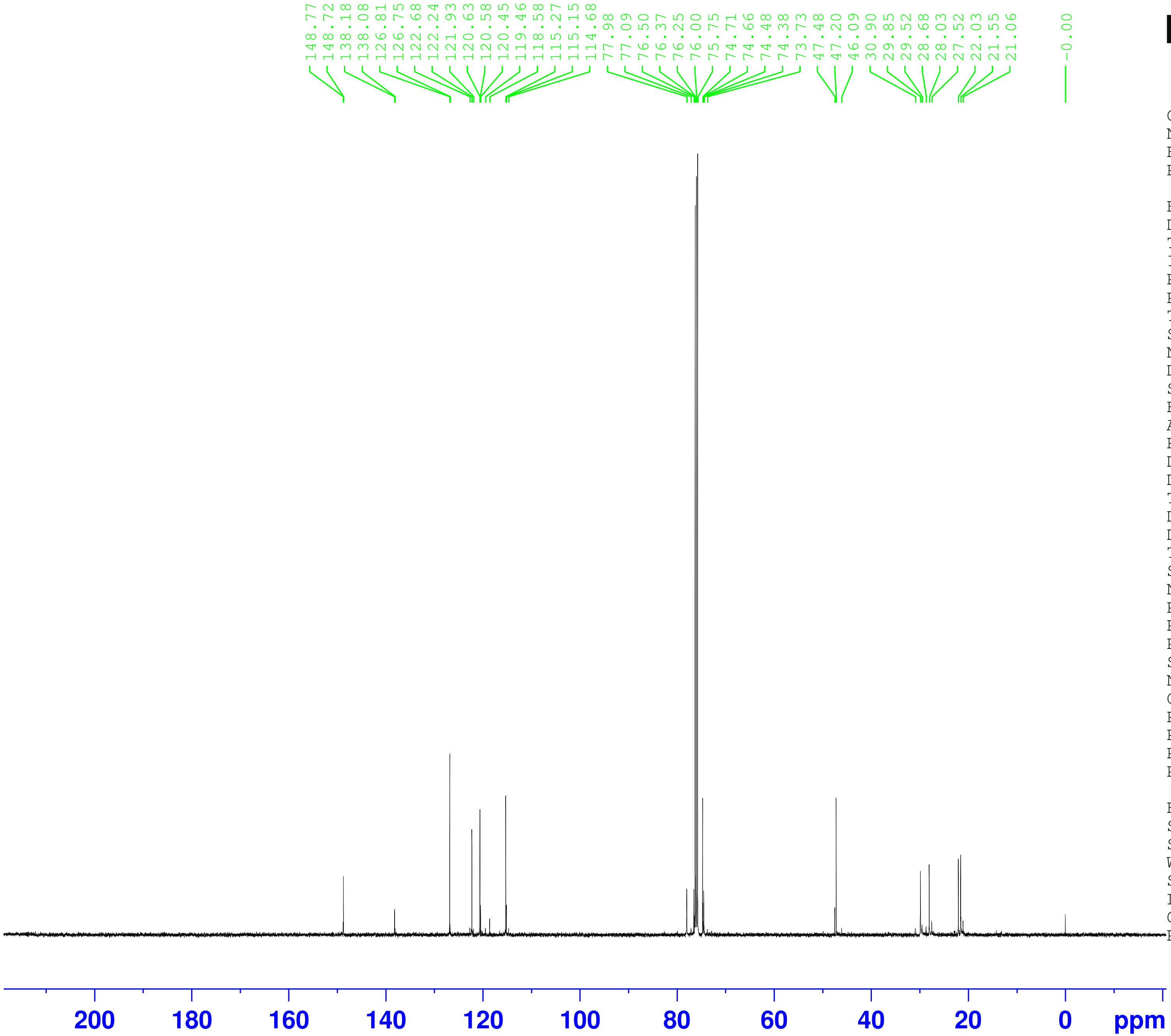

BRUKER

Current Data Parameters

NAME	JBY221-1	
EXPNO	2	
PROCNO	1	

F2 - Acquisition Parameters

Date_	20240927	
Time	3.57	h
INSTRUM	Avance	
PROBHD	Z167419_0061 (	
PULPROG	zgpg30	
TD	65536	
SOLVENT	CDCl3	
NS	1024	
DS	4	
SWH	30120.482	Hz
FIDRES	0.919204	Hz
AQ	1.0878977	sec
RG	101	
DW	16.600	usec
DE	6.50	usec
TE	298.0	K
D1	2.00000000	sec
D11	0.03000000	sec
TD0	1	
SFO1	125.7779086	MHz
NUC1	13C	
P0	3.00	usec
P1	9.00	usec
PLW1	97.08999634	W
SFO2	500.1620006	MHz
NUC2	1H	
CPDPRG[2	waltz65	
PCPD2	80.00	usec
PLW2	22.69700050	W
PLW12	0.22697000	W
PLW13	0.11417000	W

F2 - Processing parameters

SI	32768	
SF	125.7654615	MHz
WDW	EM	
SSB	0	
LB	1.00	Hz
GB	0	
PC	1.40	
