# Supplementary material for: Design, synthesis, and biological activity of novel halogenated sulfite compounds
Source: PLoS One. 2025 Jul 2;20(7):e0327587. doi: 10.1371/journal.pone.0327587 (PMC12220988; doi:10.1371/journal.pone.0327587)
Supplement: Attachment — Submitted filename: Review Report.docx [file pone.0327587.s005.docx]

**Review Report**

- The manuscript details the design, synthesis, and assessment of 36 halogenated sulfite derivatives, drawing inspiration from the structures of propargite and flupentiofenox—an innovative and pertinent strategy in the development of acaricides and aphicides.
- The research illustrates distinct structure–activity relationships (SARs), indicating how particular substitutions (e.g., Cl, CF₃O, 2-fluoroethyl) augment bioactivity, supported by robust chemical rationale and justification.
- Compounds 5.16 and 5.32 are prominently identified as lead candidates. Compound 5.32 exhibits remarkable and uncommon dual acaricidal and aphicidal properties within this class of compounds.
- The analytical characterization is sufficient and substantiates the structural assertions.
- The biological screening is well-structured; however, additional information regarding assay conditions (replicates, controls, statistical analysis) would enhance reproducibility.
- The assessment of crop safety for cowpea seedlings is pertinent; however, it is advisable to include additional discussion on broader phytotoxicity and environmental safety.
- The conclusion is robust but would improve with a concise discussion of the mechanism of action, prospective research, and potential applications.
- The language is generally clear; however, a brief editing session is advisable to enhance the flow and expression in the abstract and conclusion.

Minor Revisions — The manuscript is scientifically robust and offers significant insights; several improvements in clarity, depth, and contextual framing will enhance the final version.
