# Supplementary material for: Design, synthesis, and biological activity of novel halogenated sulfite compounds
Source: PLoS One. 2025 Jul 2;20(7):e0327587. doi: 10.1371/journal.pone.0327587 (PMC12220988; doi:10.1371/journal.pone.0327587)
Supplement: Attachment — Submitted filename: Response to Reviewers.docx [file pone.0327587.s007.docx]

Dear Editor,

Thank you for your attention and the reviewer’s evaluation and comments on our manuscript entitled “Design, synthesis, and biological activity of novel halogenated sulfite compounds” (PONE-D-25-15070). We have revised the manuscript according to your kind advice and reviewer’s detailed suggestions. Please find the responses to your suggestions and the reviewer’s comments. We sincerely hope this manuscript will be finally acceptable to be published in the PLOS ONE.

Thank you very much for all your help and looking forward to hearing from you soon!

Yours sincerely,

Bin Li

*1. Please ensure that your manuscript meets PLOS ONE's style requirements, including those for file naming.*

**Response**:

We have revised the style of the manuscript to meets PLOS ONE's style requirements, including those for file naming.

*2. We note that this submission includes NMR spectroscopy data. We would recommend that you include the following information in your methods section or as Supporting Information files:*

*1) The make/source of the NMR instrument used in your study, as well as the magnetic field strength.*

**Response**: The make/source of the NMR instrument and the magnetic field strength has been introduced in the “Chemical synthesis” section of materials and methods part.

*For each individual experiment, please also list: the nucleus being measured;*

**Response**: The nucleus being measured has been introduced in the “The general procedure for the synthesis of target compounds 5.01–5.36” section of materials and methods part.

*The sample concentration;*

**Response**: We have added the sample concentration in the “The general procedure for the synthesis of target compounds 5.01–5.36” section of materials and methods part.

*The solvent in which the sample is dissolved if solvent signal suppression was used;*

**Response**: The solvent in which the sample is dissolved has been introduced in the “The general procedure for the synthesis of target compounds 5.01–5.36” section of materials and methods part.

*The reference standard and the temperature.*

**Response**: We have added the reference standard and the temperature in the “Chemical synthesis” section of materials and methods part.

*2) A list of the chemical shifts for all compounds characterised by NMR spectroscopy, specifying, where relevant: the chemical shift (δ), the multiplicity and the coupling constants (in Hz), for the appropriate nuclei used for assignment.*

**Response**: The chemical shifts, the multiplicity and the coupling constants (in Hz) for all compounds characterised by NMR spectroscopy has been listed in the “The general procedure for the synthesis of target compounds 5.01–5.36” section of materials and methods part.

We have added the appropriate nuclei used for assignment in the same section.

*3)The full integrated NMR spectrum, clearly labelled with the compound name and chemical structure.*

**Response**: The full integrated NMR spectrum has been listed in the Supporting Information files as shown in **S1** and labelled with the compound name. The chemical structures were also listed in the Supporting Information files as shown in **S3**.

*We also strongly encourage authors to provide primary NMR data files, in particular for new compounds which have not been characterised in the existing literature. Authors should provide the acquisition data, FID files and processing parameters for each experiment, clearly labelled with the compound name and identifier, as well as a structure file for each provided dataset.*

**Response**: We have provided the primary NMR data files including the acquisition data, FID files and processing parameters, as shown in **S4**.

The compound name and identifier were labelled and a structure file was also provided as shown in **S3**.

*3. When completing the data availability statement of the submission form, you indicated that you will make your data available on acceptance. We strongly recommend all authors decide on a data sharing plan before acceptance, as the process can be lengthy and hold up publication timelines. Please note that, though access restrictions are acceptable now,* *your entire data will need to be made freely accessible if your manuscript is accepted for publication. This policy applies to all data except where public deposition would breach compliance with the protocol approved by your research ethics board. If you are unable to adhere to our open data policy, please kindly revise your statement to explain your reasoning and we will seek the editor's input on an exemption. Please be assured that, once you have provided your new statement, the assessment of your exemption will not hold up the peer review process.*

**Response**: We all authors decide on a data sharing plan and the entire data will be made freely accessible.

*4. Please review your reference list to ensure that it is complete and correct. If you have cited papers that have been retracted, please include the rationale for doing so in the manuscript text, or remove these references and replace them with relevant current references. Any changes to the reference list should be mentioned in the rebuttal letter that accompanies your revised manuscript. If you need to cite a retracted article, indicate the article’s retracted status in the References list and also include a citation and full reference for the retraction notice.*
**Response**: We reviewed our reference list again and ensure that it is complete and correct. There were no changes to the references in the revised manuscript.

*5. Review Comments to the Author*

*Reviewer #1: Review Report
• The manuscript details the design, synthesis, and assessment of 36 halogenated sulfite derivatives, drawing inspiration from the structures of propargite and flupentiofenox—an innovative and pertinent strategy in the development of acaricides and aphicides.
• The research illustrates distinct structure–activity relationships (SARs), indicating how particular substitutions (e.g., Cl, CF₃O, 2-fluoroethyl) augment bioactivity, supported by robust chemical rationale and justification.
• Compounds 5.16 and 5.32 are prominently identified as lead candidates. Compound 5.32 exhibits remarkable and uncommon dual acaricidal and aphicidal properties within this class of compounds.
• The analytical characterization is sufficient and substantiates the structural assertions.
• The biological screening is well-structured; however, additional information regarding assay conditions (replicates, controls, statistical analysis) would enhance reproducibility.*

**Response**: Thank you for your comments. We have provided additional information on the assay conditions such as statistical analysis methods in “Biological activity and safety testing on cowpea seedlings” sections.

*• The assessment of crop safety for cowpea seedlings is pertinent; however, it is advisable to include additional discussion on broader* *phytotoxicity and environmental safety.*

**Response**: The additional discussion on plant toxicity have been added. When a better compound is discovered, the broader and environmental safety will be further researched. Thank you very much for your advice.
*• The conclusion is robust but would improve with a concise discussion of the mechanism of action, prospective research, and potential applications.*

**Response**: Thank you for your suggestion. The lead optimization is underway. If we discover a better compound, the mechanism of action, further research results, and potential applications will be added in the conclusion section.
*• The language is generally clear; however,* *a brief editing session is advisable to enhance the flow and expression in the abstract and conclusion.*

**Response**: Thank you for your suggestion. The abstract and conclusion sections have been modified.

*Reviewer #2: Comments:
In this work the authors were designed, synthesized thirty-six halogenated propargite analogues and characterized using 1H NMR, 13C NMR spectroscopy, and HRMS. Among these thirty-six compounds two compounds exhibiting good insecticidal activity. Therefore, through SAR design authors discovered two interesting lead compounds (compounds 5.16 and 5.32) for insecticides. I recommend to publish in the PLOS ONE after minor revision.
Other comments:
1. I have not seen chemical structure at Figure 1. Design of target compounds: introduction of halogens*

**Response**: The revisied Figure 1 has been uploaded successfully this time.

*2. Scheme 1. Try to mention on the arrow “a” “b” and “c”*

**Response:** Thank you for pointing out these mistakes. The missing letters “a” “b” and “c” on the arrow are added.
*3.* *You write one number after digit in the 13C data*

**Response**: we have checked and make sure of that we all write two number after digit in the ^13^C data.
